# Biomarker evidence for the pathophysiological mechanisms of insomnia: a systematic review and meta-analysis

**DOI:** 10.3389/fimmu.2026.1889366

**Published:** 2026-08-19

**Authors:** Ying Wang, Pei-Chao Wang, Jia-Fu Ji, Xu Feng, Zi-Heng Wang, Tian-Shu Ma, Dong-Liang Diao, Lin Yi

**Affiliations:** 1 School of Acupuncture-Moxibustion and Tuina, Changchun University of Chinese Medicine, Changchun, China; 2 Affiliated Hospital of Shandong University of Traditional Chinese Medicine, Jinan, China; 3 Innovation and Practice Center, Changchun University of Chinese Medicine, Changchun, China

**Keywords:** biomarkers, inflammation, insomnia, meta-analysis, systematic review

## Abstract

**Background:**

Insomnia is increasingly recognized as a systemic disorder involving persistent hyperarousal, neuroendocrine dysregulation, immune activation, and impaired stress adaptation. However, circulating biomarkers reflecting these interconnected biological processes have not been comprehensively synthesized.

**Methods:**

We conducted a systematic review and meta-analysis of observational studies comparing circulating biomarkers between adults with insomnia and healthy controls. Standardized mean differences (SMDs) with 95% confidence intervals (CIs) were pooled using random- or fixed-effects models as appropriate. Subgroup analyses, sensitivity analyses, and Grading of Recommendations Assessment, Development and Evaluation (GRADE) assessments were performed to evaluate the robustness and certainty of the evidence.

**Results:**

Thirty-two studies involving 4578 individuals with insomnia and 28382 healthy controls were included. Cortisol levels were significantly elevated (SMD = 3.65, 95% CI: 2.35 to 4.95). Inflammatory biomarkers, including interleukin-6 (IL-6), tumor necrosis factor-α (TNF-α), C-reactive protein (CRP), interleukin-1β (IL-1β), interleukin-2 (IL-2), and interferon-γ (IFN-γ), were significantly increased, whereas interleukin-4 (IL-4) and interleukin-10 (IL-10) showed no significant differences. Neuropeptide Y (NPY) was significantly reduced (SMD = −0.85, 95% CI: −1.27 to −0.43), while corticotropin-releasing hormone (CRH) and brain-derived neurotrophic factor (BDNF) showed no significant pooled differences. TNF-α, IL-1β, IL-6, CRP, and NPY showed generally consistent results in sensitivity analyses.

**Conclusions:**

Insomnia is associated with alterations in circulating biomarkers reflecting hypothalamic-pituitary-adrenal (HPA) axis activity, low-grade systemic inflammation, and potential reductions in stress-buffering capacity. This multidomain biomarker pattern provides insights into the biological basis of insomnia and highlights potential candidate biomarkers for future biological stratification.

**Systematic review registration:**

https://www.crd.york.ac.uk/prospero/, identifier CRD420261396725.

## 1 Introduction

Insomnia is one of the most common sleep disorders and is characterized by persistent difficulties in initiating or maintaining sleep, accompanied by impaired daytime functioning (1, 2). Affecting a substantial proportion of the adult population worldwide, insomnia is associated with considerable personal, societal, and economic burden and has been linked to an increased risk of psychiatric, cardiovascular, metabolic, and neurodegenerative disorders (3). Sleep is a fundamental biological process essential for maintaining homeostasis, and both its quantity and quality are important for the normal functioning of multiple physiological systems (4, 5). The regulation of sleep depends on a highly coordinated, multilevel biological network involving molecular circadian mechanisms, neurotransmitter signaling, neuroendocrine pathways, immune responses, and neural circuits (4, 6, 7). Disruption of this regulatory network may contribute to systemic alterations that extend beyond subjective sleep complaints (4, 5).

Increasing evidence suggests that these alterations may be reflected in peripheral circulation (8–10). Circulating biomarkers provide a practical and minimally invasive approach for investigating systemic biological changes associated with insomnia. Over the past two decades, numerous observational studies have examined peripheral biomarkers in individuals with insomnia (8, 9, 11, 12). However, the reported findings have been inconsistent, with substantial variation in both the magnitude and direction of observed associations (8, 9, 11). Differences in study design, participant characteristics, biological specimens, and analytical methods may contribute to this heterogeneity, making it difficult to define a consistent biological profile of insomnia.

Despite growing interest in this area, the systemic biological signature of insomnia remains incompletely characterized. Previous reviews have primarily focused on specific pathways or individual biomarkers, which may not fully capture the complexity of biological alterations associated with insomnia (13, 14). Because these pathways are highly interconnected, integrating evidence across multiple biomarker domains may help identify more reproducible and biologically meaningful patterns. Therefore, we conducted a systematic review and meta-analysis to compare circulating biomarkers between individuals with insomnia and healthy controls. By synthesizing evidence across diverse biological domains, this study aimed to characterize the systemic alterations associated with insomnia and to provide a more comprehensive understanding of its underlying pathophysiology.

## 2 Materials and methods

### 2.1 Protocol registration

This systematic review and meta-analysis was conducted in accordance with the Preferred Reporting Items for Systematic Reviews and Meta-Analyses (PRISMA) guidelines (15). The protocol was registered in the International Prospective Register of Systematic Reviews (PROSPERO; CRD420261396725) and is available in **Supplementary Material 1**. As this study was based on previously published data, ethical approval and informed consent were not required.

### 2.2 Search strategy

Eight electronic databases including Web of Science, Cochrane Library, PubMed, EMBASE, China National Knowledge Infrastructure (CNKI), VIP Database for Chinese Technical Periodicals (VIP), Wanfang Data, and Chinese Biomedical Literature Database (CBM), without language restrictions, were searched from inception to March 16, 2026. A combination of Medical Subject Headings (MeSH) and free-text terms was used. The main search terms included: (“insomnia*” OR sleeplessness OR “early awakening”) AND (neurotransmit* OR hormone* OR cytokine* OR “oxidative stress*” OR neurotrophic* OR metabolic* OR marker* OR index OR parameter* OR biomarker*). Reference lists of included studies and relevant reviews were manually screened to identify additional eligible studies. Detailed search strategies for each database are provided in **Supplementary Tables S1–S5**.

### 2.3 Eligibility criteria

#### 2.3.1 Inclusion criteria

This review included studies that compared circulating biomarker levels between patients with insomnia and healthy controls. Inclusion criteria were defined according to the ‘Population, Exposure, Comparator, Outcome, and Study design’ (PECOS) framework as follows (1): Population (P): adult patients diagnosed with insomnia according to established diagnostic criteria (e.g., DSM, ICSD, ICD) or validated sleep assessment tools, and healthy control participants (2); Exposure (E): any available circulating biomarker, including neurotransmitters, hormones, cytokines, oxidative stress markers, neurotrophic factors, and metabolic parameters (3); Comparator (C): biomarker levels in healthy controls, preferably matched for age and sex (4); Outcome (O): differences in the levels of included biomarkers between insomnia patients and healthy controls. The outcomes had to be reported as mean and standard deviation (SD) for both groups, or sufficient data had to be provided to allow estimation; and (5) Study design (S): observational studies, including cross-sectional, case-control, and cohort studies.

#### 2.3.2 Exclusion criteria

Studies were excluded if they met any of the following criteria (1): case reports, review articles, meta-analyses, editorials, letters, and conference abstracts (2); studies focusing on populations not relevant to primary insomnia (pediatric participants, pregnant or lactating women, or patients with significant comorbidities) (3); animal, cellular, or genetic studies, or studies investigating non-circulating biomarkers (4); studies without healthy control groups, lacking biomarker units, or with insufficient data to calculate mean and standard deviation (5); duplicate publications from the same cohort; or (6) studies in which biomarkers were investigated in fewer than three independent cohorts.

### 2.4 Study selection and data extraction

Data extraction was performed independently by two reviewers using a standardized electronic form. Any discrepancies were resolved through discussion or consultation with a third reviewer. The following information was collected: author, year, country, sample type, assay method, study design, diagnostic criteria, duration of insomnia, number of patients and controls, age, sex, and all biomarkers assessed in each study. For each included biomarker, values were recorded as mean (with SD or standard error), median (interquartile range), or median (minimum–maximum) for both insomnia and control groups. When results were presented only graphically, data were extracted using Engauge Digitizer (version 12.1). For studies with incomplete reporting, corresponding authors were contacted via email to obtain the necessary information.

### 2.5 Assessment of literature quality

The methodological quality of all included studies was independently assessed by two reviewers, with disagreements resolved through discussion or consultation with a third reviewer. Cross-sectional studies were evaluated using the 11-item checklist recommended by the Agency for Healthcare Research and Quality (AHRQ) and categorized as high (8–11), moderate (4–7), or low quality (0–3) (16). Cohort and case-control studies were appraised using the Newcastle-Ottawa Scale (NOS), which assesses selection, comparability, and outcome/exposure, with scores of 7–9, 4–6, and 0–3 indicating high, moderate, and low quality, respectively (17). The certainty of evidence for each outcome was further assessed using the GRADE framework (18).

### 2.6 Data synthesis and statistical analysis

Statistical analyses were performed using Stata software (version 18.0; StataCorp, College Station, TX, USA). Continuous outcomes were pooled as SMDs with 95% CIs due to methodological variability across studies. According to Cohen’s criteria, SMD values of 0.2, 0.5, and ≥ 0.8 are considered to represent small, moderate, and large effect sizes, respectively (19). Means and SDs were extracted or estimated when necessary according to the Cochrane Handbook recommendations. Between-study heterogeneity was assessed using Cochran’s Q test and the I² statistic, with I² >50% or p <0.10 indicating substantial heterogeneity (18, 20). A DerSimonian and Laird random-effects model was applied for all analyses (21). Subgroup analyses were conducted based on country, sample type, assay method, and study design. Sensitivity analyses were performed by sequentially excluding individual studies. Publication bias was assessed using funnel plots when ≥ 10 studies were available, with Egger’s regression test applied when appropriate (22). Galbraith plots were used to identify potential outliers. The overall effect size was assessed using a Z-test, with p < 0.05 considered statistically significant. Data and code are available in **Tables 1**, **2** and **Supplementary Material 2**.

**Table 1:** Characteristics and quality assessment of included studies.

Study	Diagnostic method	Duration of insomnia	Biomarkers	IG		CG		Age (year)		Literature quality assessment	
				Male/Female	N	Male/Female	N	IG	CG	AHRQ or NOS score	Quality level
Li 2011 (23)	DSM-IV	NA	CRH, IL-1β	9/21	30	10/20	30	42.2 ± 10.5	38.9 ± 12.3	7	High quality
Liao et al., 2012 (24)	DSM-IV	1–240 m	NPY, SP	20/22	42	16/22	38	37 ± 11	35 ± 5	7	High quality
Huang 2013 (25)	DSM-IV	NA	TNF-α	9/21	30	10/20	30	42.4 ± 10.5	37.8 ± 11.2	7	High quality
Wang et al., 2014 (26)	CCMD-2-R	NA	IL-6	58/33	91	50/41	91	37 ± 12.8	38 ± 9.8	7	High quality
Huang et al., 2015 (27)	DSM-IV	62.5 ± 62.0 m	NPY	20/22	42	16/22	38	37.64 ± 10.6	35.74 ± 10.4	7	High quality
Zhang 2015 (28)	ICD-10/DSM-IV	≥ 6 m	BDNF	54/117	171	13/26	39	49.6 ± 11.7	50.7 ± 8.4	7	High quality
Wang et al., 2015 (29)	DSM-IV	NA	IL-2, IL-6	16/22	38	20/22	42	43.76 mean	42.04 mean	7	High quality
Wang 2015 (30)	DSM-IV	> 6 m	BDNF	7/19	26	25/47	72	49.69 ± 11.67	48.68 ± 11.85	7	High quality
Chen et al., 2016 (11)	ICSD-3	≥ 6 m	CRH, ACTH, Cortisol, TRH, TSH, Total T3, Total T4, GnRH	6/15	21	7/13	20	41.8 ± 10.4	38.1 ± 10.4	7	High quality
Yang et al., 2016 (31)	ICD-10	NA	NPY, SP	13/17	30	16/14	30	20–49	20–49	7	High quality
Liu et al., 2019 (32)	PSQI>7	NA	hsCRP, IL-6	31/9	40	37/27	64	63.6 ± 9.23	63.0 ± 9.62	6	Moderate quality
Li 2019 (33)	ICSD-3	>3 m	s100β, GFAP, GDNF, BDNF	15/34	49	11/21	32	47.16 ± 12.05	46.66 ± 8.75	7	High quality
Masyutina et al., 2021 (34)	ICSD-3	5 m to 3.5 y	Cortisol, Melatonin, IL-6	32/23	55	34/16	50	31.6 ± 7.4	33.2 ± 6.6	9	High quality
Ren et al., 2021 (35)	ICSD-3	NA	Adenosine, ADA, IL-1β, IL-2, IL-4, IL-6, IL-10, IL-12, TNF-α, IFN-γ	18/37	55	18/37	55	46.0 ± 12.5	46.2 ± 10.9	7	High quality
Xia et al., 2021 (36)	ICSD-3	NA	TNF-α, RANTES, SAA, GM-CSF	27/38	65	19/20	39	41.4 ± 12.1	42.2 ± 14.3	7	High quality
Liu 2021 (37)	Chinese guideline for the diagnosis and treatment of adult insomnia (2017)	NA	T3, T4, TSH, ALT, AST, Cr, UA, LDL, CRP, D-D, Hcy, BDNF	25/43	68	42/42	84	52.84 ± 11.57	52.76 ± 9.44	7	High quality
Kong 2021 (38)	ICSD-3	≥ 3 m	CRH, GFAP	23/37	60	15/15	30	51.5 ± 10.1	49.6 ± 12.8	7	High quality
Qin et al., 2021 (39)	Chinese guideline for the diagnosis and treatment of adult insomnia (2017)	NA	miR-147, IL-6, CRP, TGF-β1, IL-17	41/45	86	37/47	84	71.3 ± 6.17	70.96 ± 7.35	7	High quality
Fan et al., 2021 (40)	PSQI>7	5.45 ± 4.87 y	Irisin, BDNF	30/38	68	13/15	28	52.12 ± 10.68	51.93 ± 9.05	7	High quality
He et al., 2022 (41)	ICSD-3	NA	IL-1RA, IL-4, IL-5, IL-10, IL-13, IL-28A, TGF-β1, GM-CSF, IFN-γ, RANTES	20/43	63	22/20	42	41.3 ± 12.3	41.4 ± 14.1	7	High quality
Lu et al., 2022 (42)	Chinese guideline for the diagnosis and treatment of adult insomnia (2017)	≥ 3 m	Cortisol, BNDF	17/32	49	19/31	50	47.17 ± 11.65	46.58 ± 11.36	7	High quality
Yin et al., 2023 (43)	NA	NA	CRP	279/2694	2973	13887/13147	27034	48.64 ± 18.04	47.65 ± 18.78	8	High quality
He et al., 2023 (44)	DSM-5	8 ± 2.5 m	CRH, Cortisol	20/26	46	22/28	50	34.22 ± 5.28	35.18 ± 7.25	7	High quality
Jiang et al., 2023 (45)	ICSD-3	10.99 ± 4.26 y	CRH, ACTH, Cortisol	42/46	88	37/43	80	76.30 ± 5.30	76.21 ± 6.16	7	High quality
Yu et al., 2024 (46)	ICSD-3	>3 m	Cortisol, CRH, Copeptin	19/42	61	10/17	27	46.1 ± 8.2	46.9 ± 8.2	7	High quality
Zhang et al., 2024a (9)	ICSD-3	NA	Cortisol, CRH, Copeptin	14/19	33	13/21	34	50.1 ± 11.0	48.7 ± 11.2	7	High quality
Zhang et al., 2024b (47)	NA	NA	ROS, IL-2, IL-4, IL-10, IFN-γ	22/23	45	11/9	20	61.62 ± 10.06	59.95 ± 10.52	7	High quality
Fang 2024 (48)	Chinese guideline for the diagnosis and treatment of adult insomnia (2017)	8.5 ± 4.7 m	IL-1β, IL-6, IL-8, TNF-α, IFN-γ	20/20	40	18/21	39	37.8 ± 12.03	39.56 ± 14.23	7	High quality
Bai 2024 (49)	ICSD-3	NA	IL-1β, IL-6, LPS, LBP, Zonulin, s100β	4/18	22	8/14	22	46.82 ± 9.43	41.50 ± 8.25	7	High quality
Afsari et al., 2025 (50)	ICSD-3	NA	1, 3-β-D-glucan, IL-1β	4/16	20	8/12	20	47.9 ± 9.5	43.89 ± 12.39	7	High quality
Wang et al., 2025 (51)	ICSD-3	≥ 3 m	IL-1β, IL-6, CRP, TNF-α, Cortisol, IL-4, IL-10, BDNF	12/27	39	14/23	37	38.05 ± 12.839	38.27 ± 13.478	9	High quality
Zhang et al., 2026 (8)	ICSD-3	25 (15, 75) m	CRP, IL-1β, IL-6, IL-8, IL-10, TNF-α, IFN-γ	15/17	32	14/17	31	35 ± 8	34 ± 10	9	High quality

IG, insomnia group; CG, control group; N, number; AHRQ, Agency for Healthcare Research and Quality; NOS, Newcastle-Ottawa Scale; DSM-IV, Diagnostic and Statistical Manual of Mental Disorders, Fourth Edition; NA, not available; CRH, Corticotropin-Releasing Hormone; IL-1β, interleukin-1β; m, month; NPY, Neuropeptide Y; SP, Substance P; TNF-α, Tumor Necrosis Factor-alpha; CCMD-2-R, Chinese Classification of Mental Disorders, Second Edition, Revised; IL-6, Interleukin-6; ICD-10, International Classification of Diseases, 10th Revision; BDNF, Brain-Derived Neurotrophic Factor; IL-2, Interleukin-2; ACTH, Adrenocorticotropic Hormone; TRH, Thyrotropin-Releasing Hormone; TSH, Thyroid-Stimulating Hormone; T3, Triiodothyronine; T4, Thyroxine; GnRH, Gonadotropin-Releasing Hormone; PSQI, Pittsburgh Sleep Quality Index; hsCRP, high-sensitivity C-reactive protein; ICSD-3, International Classification of Sleep Disorders, Third Edition; S100β, S100 calcium-binding protein B; GFAP, Glial Fibrillary Acidic Protein; GDNF, Glial Cell Line-Derived Neurotrophic Factor; y, years; ADA, Adenosine Deaminase; IL-4, Interleukin-4; IL-10, Interleukin-10; IL-12, Interleukin-12; IFN-γ, Interferon-gamma; RANTES, Regulated upon Activation, Normal T cell Expressed and Secreted; SAA, Serum Amyloid A; GM-CSF, Granulocyte-Macrophage Colony-Stimulating Factor; ALT, Alanine Aminotransferase; AST, Aspartate Aminotransferase; Cr, Creatinine; UA, Uric Acid; LDL, Low-Density Lipoprotein; CRP, C-reactive protein; D-D, D-dimer; Hcy, Homocysteine; miR-147, microRNA-147; TGF-β1, Transforming Growth Factor-beta 1; IL-17, Interleukin-17; IL-1RA, Interleukin-1 Receptor Antagonist; IL-5, Interleukin-5; IL-13, Interleukin-13; IL-28A, Interleukin-28A; CLIA, Chemiluminescence Immunoassay; DSM-5, Diagnostic and Statistical Manual of Mental Disorders, Fifth Edition; ROS, Reactive Oxygen Species; IL-8, Interleukin-8; LPS, Lipopolysaccharide; LBP, Lipopolysaccharide-Binding Protein.

**Table 2:** Summary of circulating biomarker findings in included studies.

Study	Country	Sample type	Assay method	Study design	Findings	
					IG	CG
Li 2011 (23)	China	Serum	ELISA	Case-control study	CRH: 126.7 (103.4, 180.6) ng/L IL-1β: 32.4 (31.1, 33.4) ng/L	CRH: 69.5 (48.7, 76.7) ng/L IL-1β: 26.9 (24.7, 28.5) ng/L
Liao et al., 2012 (24)	China	Serum	ELISA	Case-control study	NPY: 5.6 ± 3.9 μg/mL	NPY: 8.7 ± 5.0 μg/mL
Huang 2013 (25)	China	Serum	ELISA	Case-control study	TNF-α: 40.9 ± 2.9 pg/mL	TNF-α: 33.0 ± 2.5 pg/mL
Wang et al., 2014 (26)	China	Serum	ELISA	Case-control study	IL-6: 2.5 ± 1.2 pg/mL	IL-6: 0.8 ± 0.2 pg/mL
Huang et al., 2015 (27)	China	Plasma	ELISA	Case-control study	NPY: 5.11 ± 2.87 ng/mL	NPY: 7.01 ± 3.44 ng/mL
Zhang 2015 (28)	China	Serum	ELISA	Case-control study	BDNF: 1537 ± 130 pg/mL	BDNF: 1119 ± 264 pg/mL
Wang et al., 2015 (29)	China	Serum	ELISA	Case-control study	IL-2: 97.78 ± 30.61 ng/mL IL-6: 6.05 ± 2.01 pg/mL	IL-2: 77.82 ± 35.85 ng/mL IL-6: 4.45 ± 2.18 pg/mL
Wang 2015 (30)	China	Serum	ELISA	Case-control study	BDNF: 10.09 (8.06) µg/L	BDNF: 13.10 ± 2.85 µg/L
Chen et al., 2016 (11)	China	Serum	ELISA	Case-control study	CRH: 123.7 (102.8, 176.5) ng/L Cortisol: 230.8 ± 13.4 µg/L	CRH: 64.5 ± 32.9 ng/L Cortisol: 125.4 ± 12.9 µg/L
Yang et al., 2016 (31)	China	Serum	ELISA	Case-control study	NPY: 5.72 ± 2.26 µg/mL	NPY: 8.81 ± 2.35 µg/mL
Liu et al., 2019 (32)	China	Serum	NA	Case-control study	IL-6: 4.10 (5.22) pg/mL	IL-6: 2.26 (2.57) pg/mL
Li 2019 (33)	China	Serum	ELISA	Case-control study	BDNF: 19.63 ± 6.52 ng/mL	BDNF: 46.49 ± 13.33 ng/mL
Masyutina et al., 2021 (34)	Russia	Serum	ELISA	Cross-sectional study	Cortisol: 581.5 ± 110.6 nmol/L IL-6: 5.6 ± 0.9 pg/mL	Cortisol: 323.5 ± 108.1 nmol/L IL-6: 2.8 ± 0.8 pg/mL
Ren et al., 2021 (35)	China	Plasma	Luminex multiplex immunoassay	Case-control study	IL-1β: 1.3 (1.0, 1.7) pg/mL IL-2: 3.0 ± 0.5 pg/mL IL-4: 0.9 (0.5, 1.3) pg/mL IL-6: 4.0 ± 1.0 pg/mL IL-10: 0.8 ± 0.3 pg/mL TNF-α: 2.7 ± 0.7 pg/mL IFN-γ: 17.4 (12.1, 28.5) pg/mL	IL-1β: 1.0 (0.8, 1.4) pg/mL IL-2: 2.7 ± 0.4 pg/mL IL-4: 0.7 (0.6, 1.1) pg/mL IL-6: 3.5 ± 0.8 pg/mL IL-10: 0.7 ± 0.3 pg/mL TNF-α: 2.4 ± 0.5 pg/mL IFN-γ: 13.9 (10.9, 25.5) pg/mL
Xia et al., 2021 (36)	China	Serum	RayBiotech Chip Detection Test	Case-control study	TNF-α: 427.41 ± 203.60 pg/mL	TNF-α: 322.51 ± 117.74 pg/mL
Liu 2021 (37)	China	Serum	ELISA	Case-control study	BDNF: 1536.97 ± 291.28 ng/L	BDNF: 1705.07 ± 331.86 ng/L
		Peripheral venous blood	NA		CRP: 3.23 ± 7.57 mg/L	CRP: 2.98 ± 7.34 mg/L
Kong 2021 (38)	China	Serum	ELISA	Case-control study	CRH: 0.19 (0.17, 0.22) µg/mL	CRH: 0.07 (0.06, 0.11) µg/mL
Qin et al., 2021 (39)	China	Serum	ELISA	Case-control study	IL-6: 43.55 ± 6.27 pg/mL CRP: 3.27 ± 1.13 mg/L	IL-6: 21.38 ± 5.51 pg/mL CRP: 2.36 ± 0.78 mg/L
Fan et al., 2021 (40)	China	Serum	ELISA	Case-control study	BDNF: 5.01 ± 2.01 µg/L	BDNF: 8.72 ± 3.03 µg/L
He et al., 2022 (41)	China	Serum	ELISA	Case-control study	IL-4: 20.08 (5.44, 38.73) pg/mL IL-10: 8.38 (3.53, 18.26) pg/mL IFN-γ: 382.04 (257.50, 563.65) pg/mL	IL-4: 37.78 (21.61, 59.90) pg/mL IL-10: 8.73 (3.49, 14.32) pg/mL IFN-γ: 380.28 (266.21, 504.80) pg/mL
Lu et al., 2022 (42)	China	Serum	ELISA	Case-control study	BNDF: 19.96 ± 6.21 ng/mL	BNDF: 48.29 ± 12.65 ng/mL
			CLIA		Cortisol: 451.78 ± 82.65 µg/L	Cortisol: 221.19 ± 71.38 µg/L
Yin et al., 2023 (43)	China	Peripheral venous blood	Latex-enhanced nephelometry	Cross-sectional study	CRP: 0.23 (0.09, 0.52) mg/dL	CRP: 0.18 (0.07, 0.43) mg/dL
He et al., 2023 (44)	China	Plasma	ELISA	Case-control study	CRH: 130.25 ± 10.11 ng/L	CRH: 64.28 ± 9.54 ng/L
			CLIA		Cortisol: 280.65 ± 14.56 µg/L	Cortisol: 100.23 ± 10.11 µg/L
Jiang et al., 2023 (45)	China	Serum	ELISA	Case-control study	CRH: 396.27 ± 43.42 pg/mL Cortisol: 435.74 ± 52.11 ng/mL	CRH: 378.68 ± 40.28 pg/mL Cortisol: 398.05 ± 45.79 ng/mL
Yu et al., 2024 (46)	China	Serum	ELISA	Case-control study	Cortisol: 434.3 ± 23.8 ng/mL CRH: 338.7 (287.0, 380.2) pg/mL	Cortisol: 371.5 ± 13.9 ng/mL CRH: 155.4 (118.8, 177.0) pg/mL
Zhang et al., 2024a (9)	China	Serum	ELISA	Case-control study	Cortisol: 438.7 (406.0, 458.5) ng/mL CRH: 410.1 ± 86.3 pg/mL	Cortisol: 406.8 (388.2, 472.0) ng/mL CRH: 388.7 ± 200.8 pg/mL
Zhang et al., 2024b (47)	China	Serum	ELISA	Case-control study	IL-2: 315.79 ± 73.97 pg/mL IL-4: 19.97 ± 4.15 pg/mL IL-10: 362.95 ± 183.17 pg/mL IFN-γ: 275.14 ± 80.91 pg/mL	IL-2: 307.24 ± 76.68 pg/mL IL-4: 10.45 ± 4.25 pg/mL IL-10: 343.04 ± 71.35 pg/mL IFN-γ: 277.47 ± 92.89 pg/mL
Fang 2024 (48)	China	Serum	ELISA	Case-control study	IL-1β: 1.78 ± 0.89 pg/mL IL-6: 125.38 ± 93.88 pg/mL TNF-α: 12.41 ± 9.75 pg/mL IFN-γ: 4.86 ± 4.53 pg/mL	IL-1β: 1.97 ± 1.44 pg/mL IL-6: 4.07 ± 3.11 pg/mL TNF-α: 9.25 ± 9.07 pg/mL IFN-γ: 3.89 ± 1.87 pg/mL
Bai 2024 (49)	China	Serum	ELISA	Case-control study	IL-1β: 1.62 (1.02, 3.83) ng/mL IL-6: 3.02 (2.23, 3.91) ng/mL	IL-1β: 0.77 (0.41, 0.98) ng/mL IL-6: 1.73 (1.10, 2.21) ng/mL
Afsari et al., 2025 (50)	Iran	Serum	ELISA	Case-control study	IL-1β: 58.29 ± 32.05 pg/mL	IL-1β: 37.18 ± 17.77 pg/mL
Wang et al., 2025 (51)	China	Serum	ELISA	Cross-sectional study	IL-1β: 19.08 ± 2.401 pg/mL IL-6: 9.04 ± 1.061 pg/mL CRP: 1.99 ± 0.271 pg/mL TNF-α: 10.96 ± 1.875 pg/mL Cortisol: 66.50 ± 7.778 nmol/L IL-4: 7.74 ± 0.962 pg/mL IL-10: 94.30 ± 16.326 pg/mL BDNF: 7.02 ± 1.663 pg/mL	IL-1β: 16.16 ± 2.956 pg/mL IL-6: 8.48 ± 1.376 pg/mL CRP: 1.81 ± 0.357 pg/mL TNF-α: 9.70 ± 1.985 pg/mL Cortisol: 59.41 ± 13.235 nmol/L IL-4: 8.67 ± 1.280 pg/mL IL-10: 118.77 ± 28.747 pg/mL BDNF: 8.62 ± 1.741 pg/mL
Zhang et al., 2026 (8)	China	Serum	ELISA	Cross-sectional study	CRP: 0.7 (0.5, 1.3) mg/L IL-1β: 0.6 (0.4, 0.9) pg/mL IL-6: 1.5 (0.6, 3.4) pg/mL IL-10: 2.5 (0.2, 9.2) pg/mL TNF-α: 33.4 (24.0, 67.5) pg/mL IFN-γ: 177.3 (71.6, 354.4) pg/mL	CRP: 0.5 (0.5, 1.2) mg/L IL-1β: 0.2 (0.1, 0.6) pg/mL IL-6: 0.9 (0.2, 2.6) pg/mL IL-10: 4.7 (1.6, 9.1) pg/mL TNF-α: 24.5 (11.5, 55.8) pg/mL IFN-γ: 84.1 (51.3, 178.1) pg/mL

IG, insomnia group; CG, control group; ELISA, Enzyme-Linked Immunosorbent Assay; CRH, Corticotropin-Releasing Hormone; IL-1β, interleukin-1β; NPY, Neuropeptide Y; TNF-α, Tumor Necrosis Factor-alpha; IL-6, Interleukin-6; BDNF, Brain-Derived Neurotrophic Factor; IL-2, Interleukin-2; ACTH, Adrenocorticotropic Hormone; IL-4, Interleukin-4; IL-10, Interleukin-10; IFN-γ, Interferon-gamma; CRP, C-reactive protein.

## 3 Results

### 3.1 Study selection

**Figure 1:**
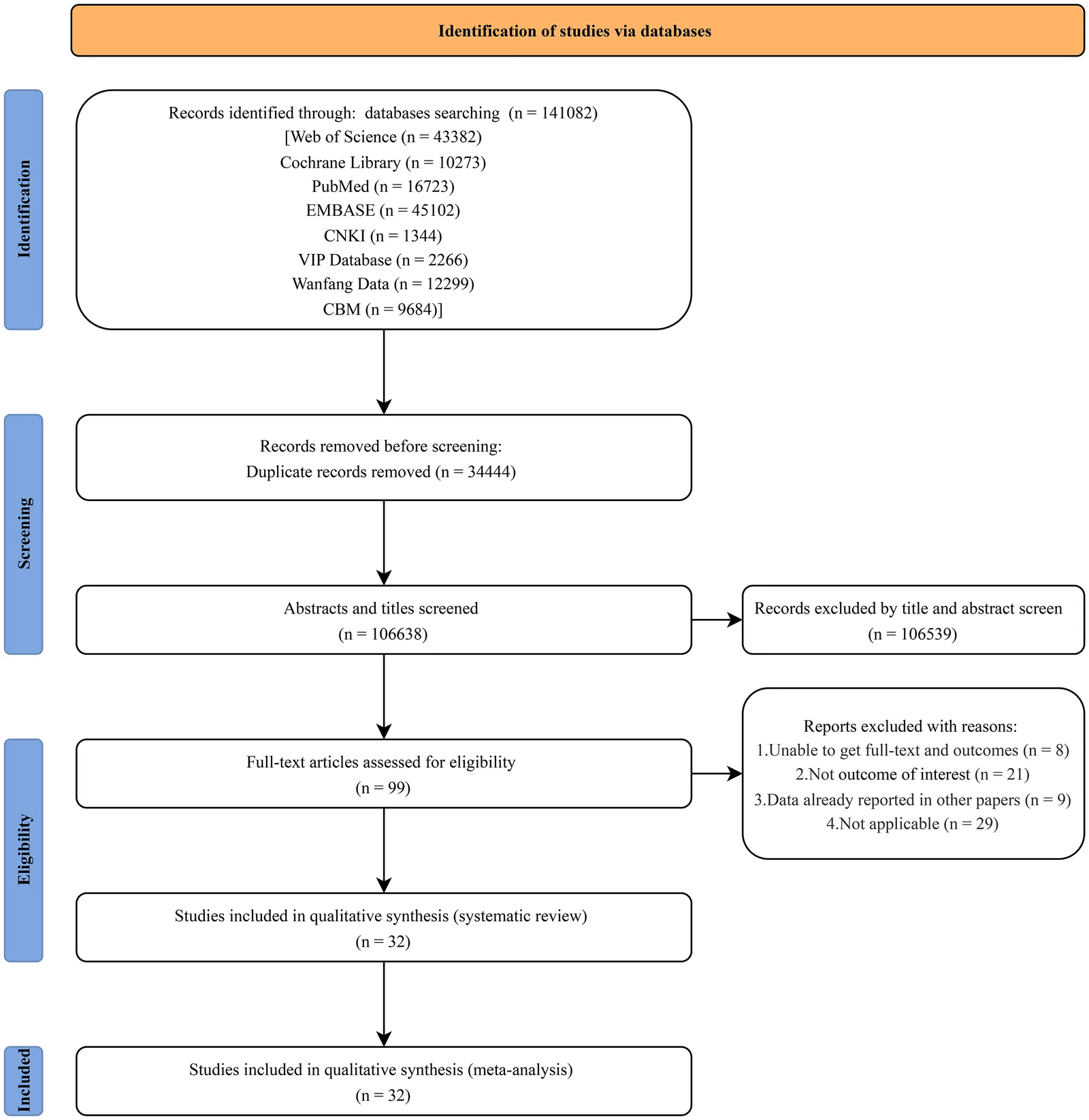
PRISMA flow diagram of study selection for the systematic review and meta-analysis.

A total of 141, 082 records were identified through database searches. After removing 34, 444 duplicates, 106, 638 records were screened based on titles and abstracts, resulting in the exclusion of 106, 539 studies. Full texts of 99 articles were assessed for eligibility. Among these, some studies were excluded due to inaccessibility, lack of relevant outcomes, duplicate data, or failure to meet the inclusion criteria. To meet the minimum requirement of including at least three independent insomnia cohorts and three healthy control cohorts per biomarker, 32 studies were ultimately included in the meta-analysis. The systematic review process is summarized in **Figure 1**.

### 3.2 Study characteristics and results of the methodological quality assessment

A total of 32 studies involving 4578 insomnia patients and 28382 healthy controls were included in the meta-analysis (8, 9, 11, 23–51). Most studies were conducted in China (n = 30), with the remaining two conducted in Iran and Russia. The majority of studies employed a case-control design (n = 28), while four were cross-sectional. Insomnia duration ranged from 1 month to over 10 years, and diagnostic criteria included the Diagnostic and Statistical Manual of Mental Disorders (4th and 5th editions; DSM-IV and DSM-5), the International Classification of Diseases (10th Revision; ICD-10), the International Classification of Sleep Disorders (3rd Edition; ICSD-3), the Chinese Classification of Mental Disorders (2nd Edition, Revised; CCMD-2-R), and the Chinese guideline for the diagnosis and treatment of adult insomnia (2017). The mean age of participants varied between 31.6 and 76.3 years, and most studies included balanced sex distributions. Biological samples were predominantly serum (n = 28), with the remainder using plasma (n = 3) or peripheral venous blood (n = 2). Assay methods were mainly Enzyme-Linked Immunosorbent Assay (ELISA; n = 28), although some studies used Chemiluminescence Immunoassay (CLIA; n = 2), Luminex multiplex immunoassay (n = 1), RayBiotech chip detection (n = 1), or latex-enhanced nephelometry (n = 1). The following biomarkers were evaluated: cortisol (n = 8), CRH (n = 7), TNF-α (n = 6), IL-1β (n = 7), IL-2 (n = 3), IL-4 (n = 4), IL-6 (n = 10), interleukin-10 (IL-10; n = 5), IFN-γ (n = 5), CRP (n = 5), BDNF (n = 7) and NPY (n = 3). Further details are provided in **Tables 1**, **2**.

### 3.3 Meta-analysis

#### 3.3.1 Hormones

A total of 10 studies reported hormonal biomarkers, with sample sizes ranging from 21 to 88 in the insomnia group and from 20 to 80 in the control group (11, 23, 34, 38, 42, 44–47, 51). Pooled analysis showed a significant increase in cortisol levels compared with healthy controls (SMD = 3.65, 95% CI: 2.35 to 4.95, p < 0.001; I² = 97.6%). In contrast, no significant difference was observed for CRH (SMD = 0.78, 95% CI: −0.94 to 2.50, p = 0.375; I² = 98.4%).

**Figure 2:**
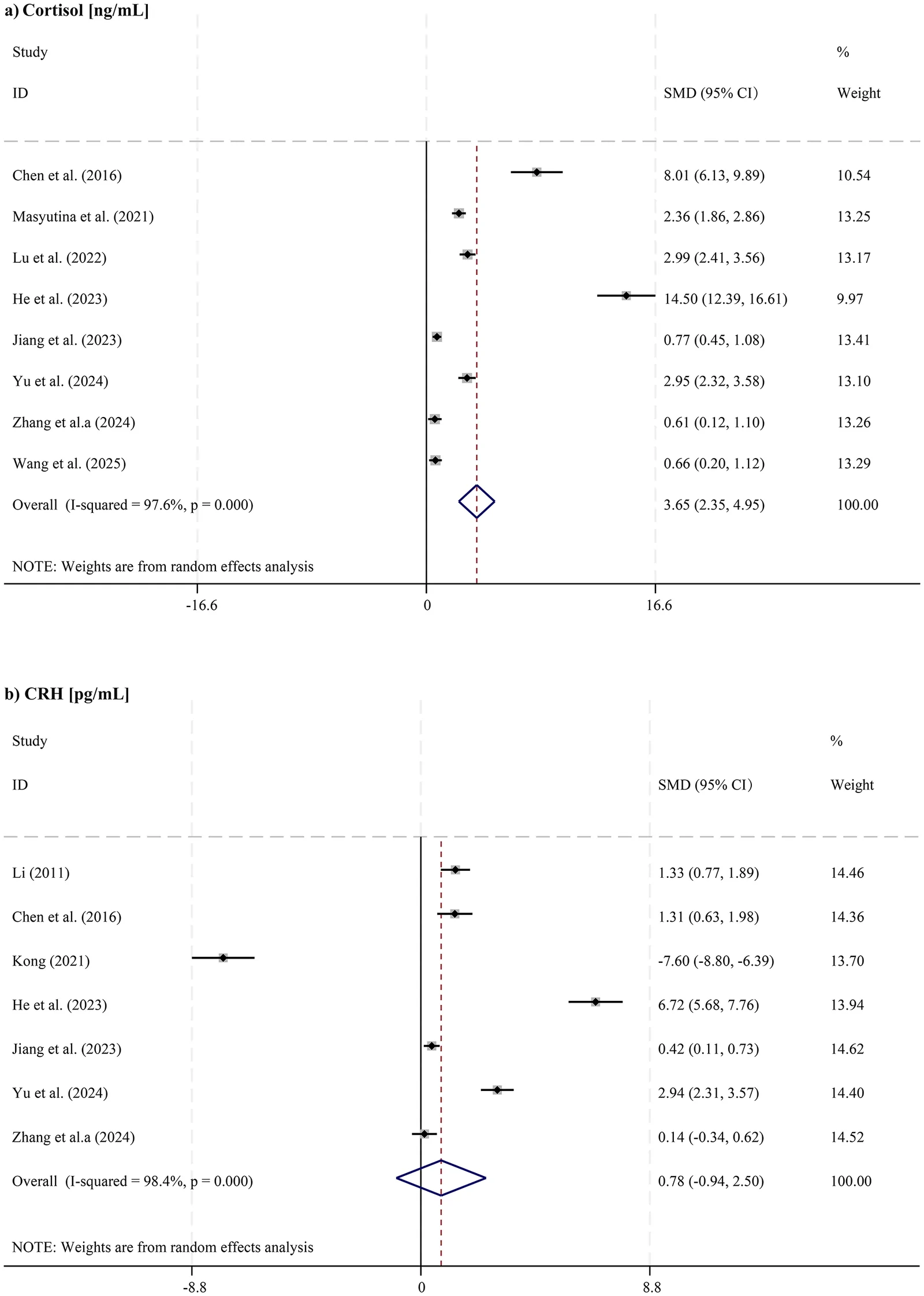
Forest plots of **(a)** cortisol and **(b)** CRH. SMD, standardized mean difference; CI, confidence interval.

Subgroup analyses for cortisol showed consistent increases across country, study design, sample type, and assay method, although heterogeneity remained high in most subgroups. For CRH, subgroup analysis according to sample type showed no significant difference for serum samples (SMD = −0.16, 95% CI: −1.72 to 1.40). Only one study reported significantly increased plasma CRH levels in patients with insomnia compared with healthy controls (SMD = 6.72, 95% CI: 5.68 to 7.76). The Galbraith plots identified a single cortisol study lying beyond the 95% CI (44). Although all CRH studies remained within the 95% CI, He et al. (44) and Kong et al. (38) exhibited relatively large deviations from the regression line. Further details are provided in **Tables 1**, **2**; **Figure 2**; **Supplementary Figures S1–S7**.

#### 3.3.2 Inflammatory factors

Levels of 8 inflammatory biomarkers were evaluated in insomnia patients and healthy controls across 18 studies, with the majority of studies evaluating more than one biomarker (8, 23, 25, 26, 29, 32, 34–37, 39, 41, 43, 47–51). The number of subjects included in the analysis varied, ranging from 20 (IL-1β) to 2973 (CRP) for patients with insomnia, and from 20 (IL-1β) to 27034 (CRP) for healthy controls. Compared with healthy controls, levels of the following markers were significantly increased in patients with insomnia: IL-6 (SMD = 1.47, 95% CI: 0.76 to 2.18, p < 0.001), IL-1β (SMD = 0.89, 95% CI: 0.36 to 1.41, p = 0.001), TNF-α (SMD = 0.82, 95% CI: 0.27 to 1.37, p = 0.003), IL-2 (SMD = 0.50, 95% CI: 0.18 to 0.81, p = 0.002), CRP (SMD = 0.40, 95% CI: 0.08 to 0.72, p = 0.013), and IFN-γ (SMD = 0.22, 95% CI: 0.02 to 0.42, p = 0.029). No significant differences were observed for concentrations of IL-4 (SMD = 0.27, 95% CI: −0.86 to 1.40, p = 0.635) and IL-10 (SMD = –0.19, 95% CI: –0.66 to 0.28, p = 0.432). Heterogeneity was high in IL-4 (I² = 95.9%), IL-6 (I² = 95.7%), TNF-α (I² = 88.0%), IL-1β (I² = 86.1%) and CRP (I² = 84.3%), IL-10 (I² = 81.8%), whereas low in IL-2 (I² = 31.3%) and IFN-γ (I² = 2.4%).

Subgroup analyses showed that the associations for TNF-α, IL-1β, IL-6, and CRP were generally consistent across most subgroups. For IL-2 and IFN-γ, pooled estimates were not statistically significant in subgroup analyses based on serum samples and ELISA-based assays. The results for IL-4 and IL-10 varied across subgroups. Only IL-10 showed a statistically significant difference in cross-sectional studies (SMD = −0.71, 95% CI: −1.39 to −0.03). Galbraith plots showed no major outliers for most inflammatory biomarkers, except for one study in TNF-α (25). Moderate dispersion was observed for IL-1β, IL-4, IL-6, and IL-10, whereas IL-2, IFN-γ, and CRP showed relatively stable distributions. Further details are provided in **Tables 1**, **2**; **Figures 3**–**6**; **Supplementary Figures S8–S40**.

**Figure 3:**
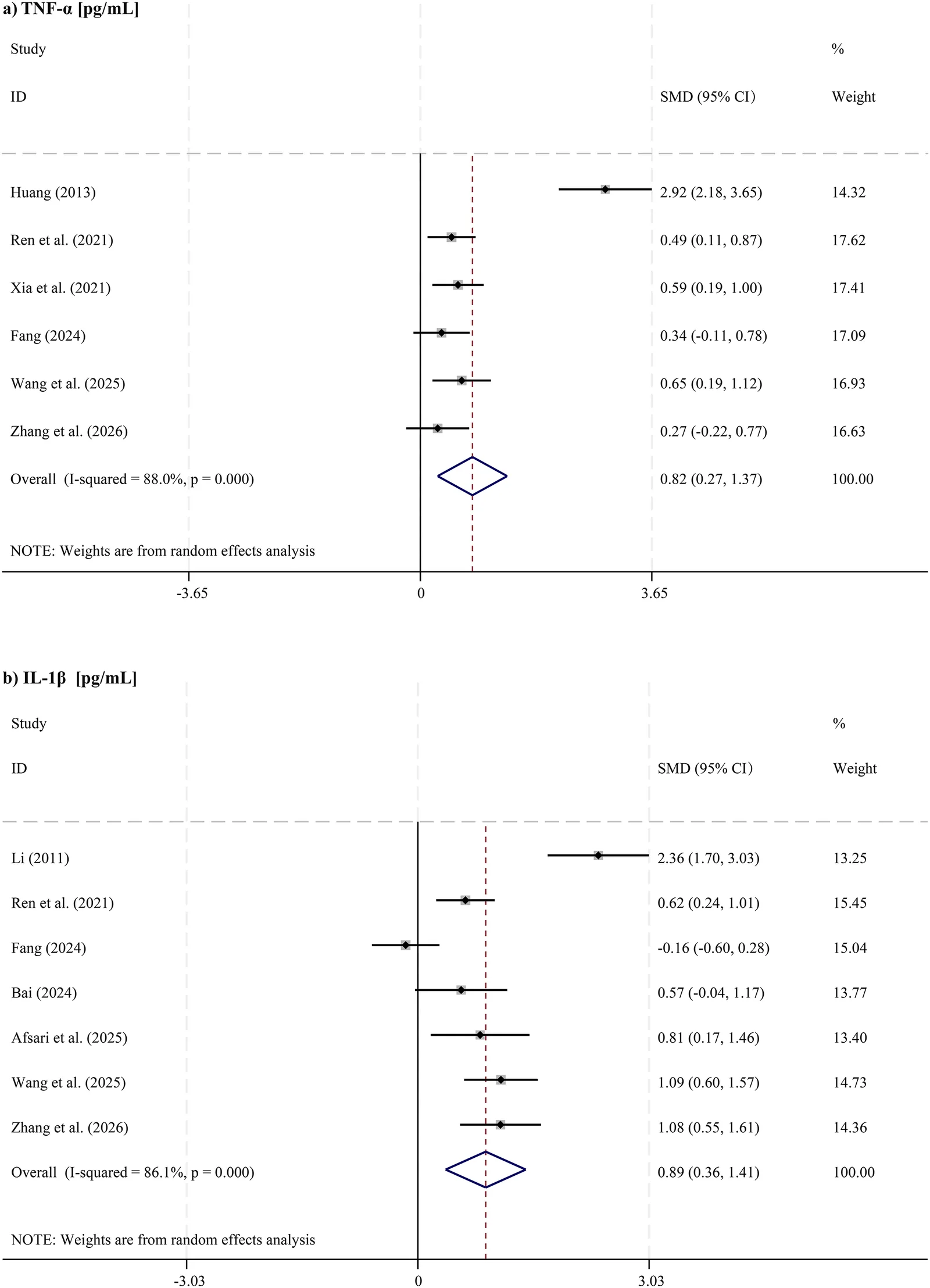
Forest plots of **(a)** TNF-α and **(b)** IL-1β. SMD, standardized mean difference; CI, confidence interval.

**Figure 4:**
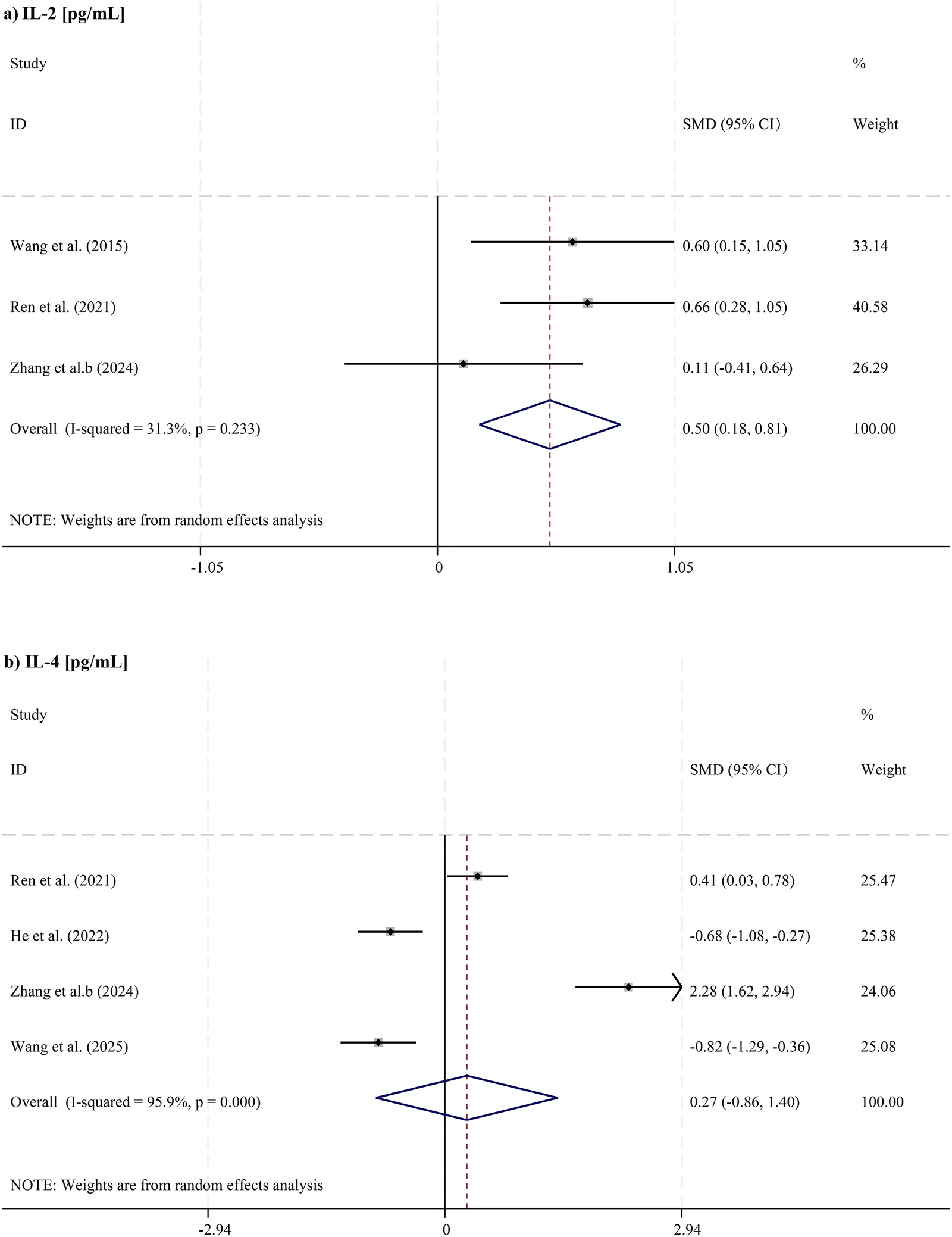
Forest plots of **(a)** IL-2 and **(b)** IL-4. SMD, standardized mean difference; CI, confidence interval.

**Figure 5:**
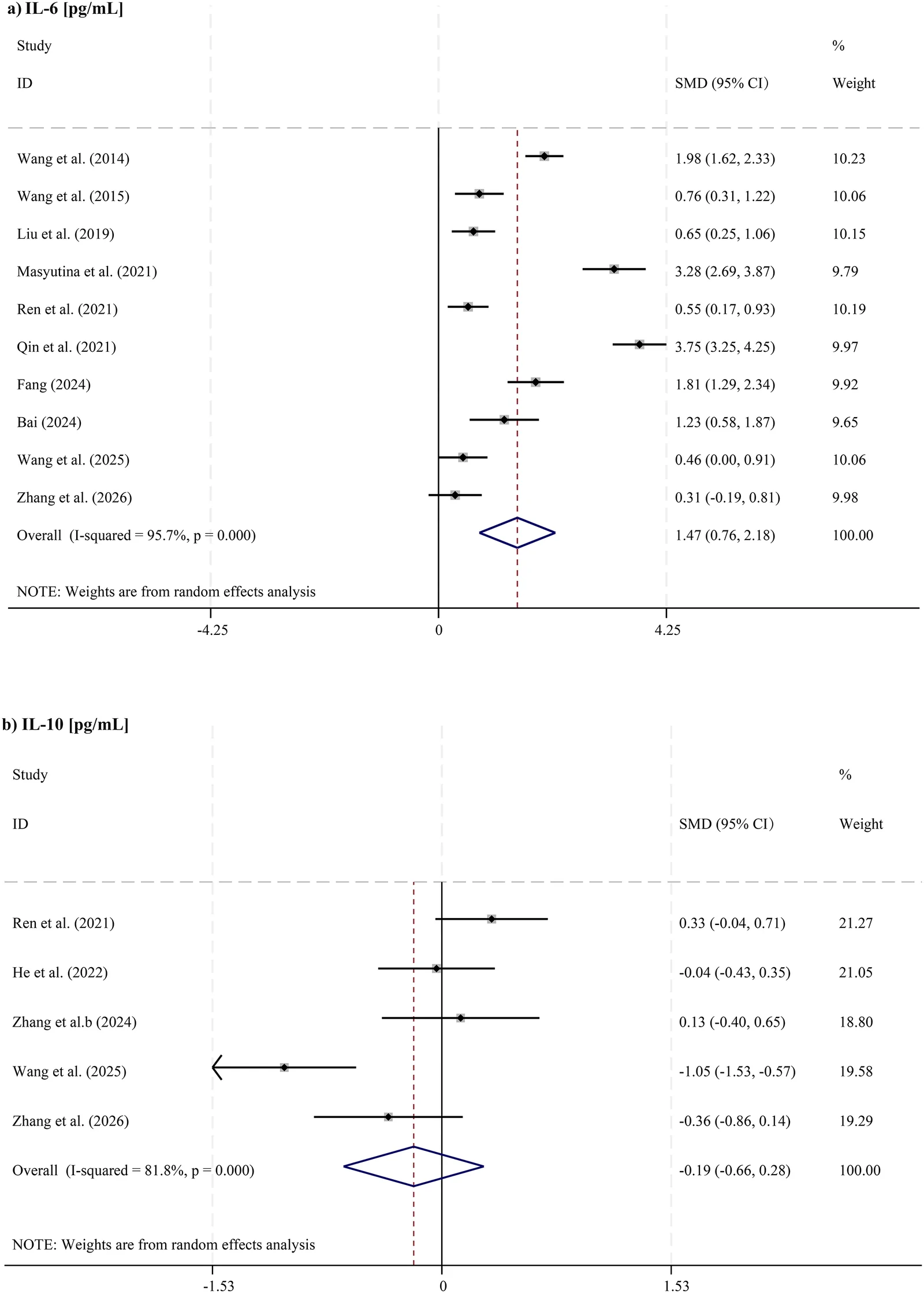
Forest plots of **(a)** IL-6 and **(b)** IL-10. SMD, standardized mean difference; CI, confidence interval.

**Figure 6:**
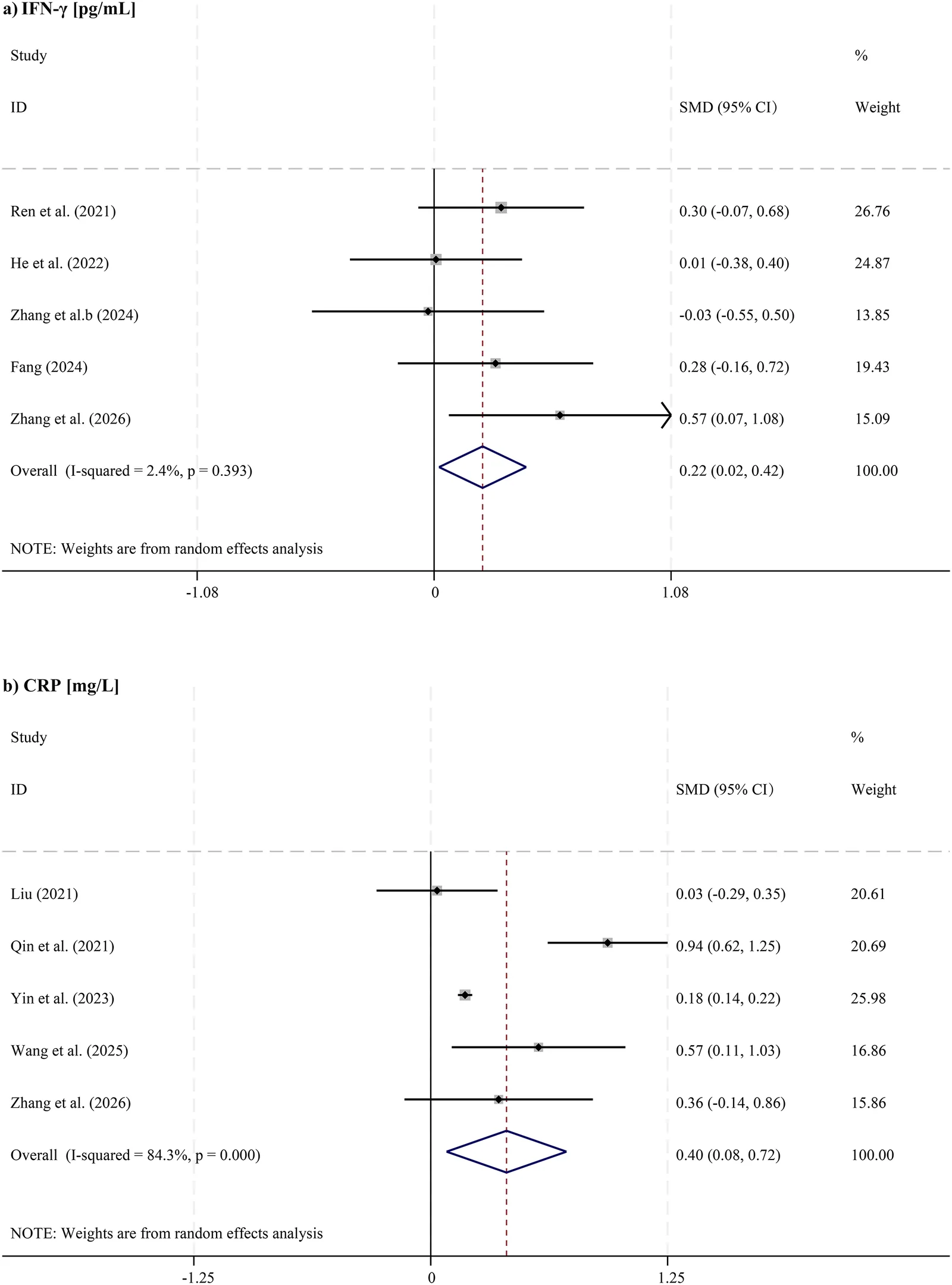
Forest plots of **(a)** IFN-γ and **(b)** CRP. SMD, standardized mean difference; CI, confidence interval.

#### 3.3.3 Other

Neurotrophic and neuropeptide factors were assessed in 10 studies, of which seven examined BDNF and three focused on NPY (24, 27, 28, 30, 31, 33, 37, 40, 42, 51). The included studies had sample sizes ranging from 26 to 171 in the insomnia groups and from 28 to 84 in the healthy controls. No significant difference was observed between patients with insomnia and healthy controls for BDNF (SMD = −0.97, 95% CI: −2.28 to 0.34, p = 0.146). In contrast, NPY levels were significantly lower in patients with insomnia than in healthy controls (SMD = −0.85, 95% CI: −1.27 to −0.43, p < 0.001). Heterogeneity was high for BDNF (I² = 98.2%) and moderate for NPY (I² = 55.4%).

**Figure 7:**
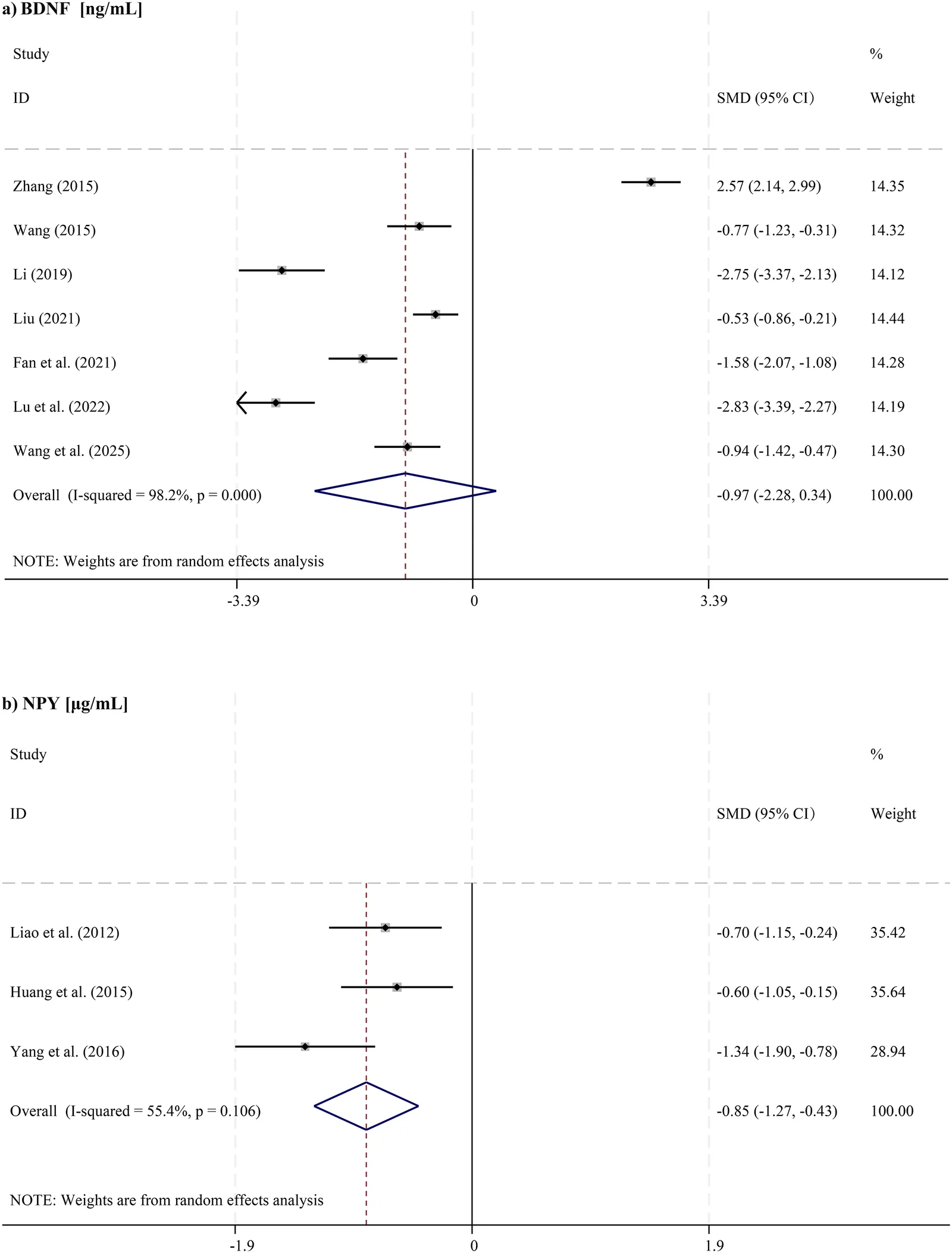
Forest plots of **(a)** BDNF and **(b)** NPY. SMD, standardized mean difference; CI, confidence interval.

Subgroup analyses revealed no significant difference for BDNF in case-control studies (SMD = −0.98, 95% CI: −2.52 to 0.56). For NPY, significantly lower levels were observed in serum samples among patients with insomnia compared with healthy controls (SMD = −1.00, 95% CI: −1.63 to −0.37). The Galbraith plots showed that all studies for BDNF and NPY were within the 95% CI. BDNF exhibited considerable dispersion around the regression line, whereas NPY showed a moderate degree of dispersion and no apparent outliers. Further details are provided in **Tables 1**, **2**; **Figure 7**; **Supplementary Figures S41–S44**.

### 3.4 Sensitivity analysis and risk of bias assessment

Sensitivity analyses indicated that the pooled estimates for most biomarkers were generally robust. Specifically, TNF-α, IL-1β, IL-6, IL-4, CRP, and NPY showed stable results, with consistent effect directions and statistical significance after exclusion of individual studies. Cortisol also remained statistically significant across sensitivity analyses; however, exclusion of He et al. (44), which showed an extremely large effect estimate, attenuated the pooled effect size (SMD = 2.34, 95% CI: 1.35 to 3.33), suggesting that this study contributed to the magnitude of the pooled estimate but did not alter the overall direction of the association. IL-10 showed consistently non-significant results after exclusion of individual studies. In contrast, IL-2 and IFN-γ demonstrated moderate sensitivity, as the direction of effects fluctuated or statistical significance varied after removal of individual studies. CRH and BDNF were more sensitive to individual studies. For CRH, exclusion of He et al. (44) resulted in a non-significant association (SMD = −0.16, 95% CI: −1.72 to 1.40), whereas exclusion of Kong et al. (38) yielded a significant positive association (SMD = 2.08, 95% CI: 0.76 to 3.41), indicating that the pooled CRH estimate was substantially influenced by individual studies.

Publication bias was assessed for IL-6, as it included at least 10 studies. Visual inspection of the funnel plot suggested no obvious asymmetry, and Egger’s test showed no significant evidence of small-study effects (p = 0.412), indicating no apparent publication bias. Detailed results are presented in **Supplementary Figures S45–S58** and **Supplementary Table S6**.

### 3.5 Certainty of evidence

According to the GRADE framework, the certainty of evidence was rated as low at the outset given that all included studies were observational in design. Most outcomes were further downgraded due to serious inconsistency and imprecision, with no upgrading factors identified. Overall, the certainty of evidence ranged from low to very low across inflammatory and neuroendocrine biomarkers, as detailed in **Supplementary Table S7**.

## 4 Discussion

### 4.1 Synopsis of circulating biomarkers

In this systematic review and meta-analysis, we synthesized evidence on multiple circulating biomarkers in adults with insomnia, spanning neuroendocrine regulation, inflammatory processes, neuroplasticity, and stress resilience. This review examined biological alterations associated with insomnia from a systems-level perspective rather than focusing on a single molecular domain. The most consistent abnormalities were observed in the HPA axis and inflammatory domains. Among neuroendocrine markers, circulating cortisol levels were significantly elevated in individuals with insomnia, whereas no significant overall difference was detected for CRH. Among inflammatory markers, concentrations of IL-6, TNF-α, CRP, IL-1β, IL-2, and IFN-γ were significantly increased compared with healthy controls, whereas no significant differences were observed for the anti-inflammatory cytokines IL-4 and IL-10. In the neuroplasticity and stress-resilience domains, no significant pooled difference was identified for BDNF, whereas circulating NPY levels were significantly lower in individuals with insomnia.

### 4.2 Interpretation in context

Our findings support a systems-level model of insomnia in which multiple interacting biological processes may contribute to the development and persistence of sleep disturbance. These processes may involve dysfunction of interconnected brain regions, including the hypothalamus, hippocampus, amygdala, and prefrontal cortex, which play key roles in stress regulation, emotional processing, and sleep-wake control (52–54).

One of the most prominent findings of this review was the elevation of circulating cortisol, which is consistent with the hyperarousal model of insomnia. Cortisol is the principal effector hormone of the HPA axis and reflects sustained activation of the stress-response system (55). Persistent cognitive and emotional arousal may promote prolonged cortisol secretion, thereby increasing wakefulness, impairing sleep continuity, and weakening negative feedback regulation mediated by the hippocampus and prefrontal cortex (10, 56, 57). In contrast, no significant overall difference was observed for CRH. This apparent discrepancy may be related to the biological and technical characteristics of CRH measurement, including its short half-life, pulsatile secretion pattern, and low peripheral concentrations (58–60). These findings suggest that alterations in HPA axis activity may contribute to insomnia, with cortisol representing a more stable circulating marker.

Beyond neuroendocrine alterations, this review identified significant elevations in multiple inflammatory markers, including IL-6, TNF-α, CRP, IL-1β, IL-2, and IFN-γ. These findings suggest that insomnia may be associated with broad activation of inflammatory pathways involving both innate and adaptive immune responses. Among these biomarkers, IL-6, TNF-α, and CRP demonstrated relatively consistent associations across analyses, suggesting that they may represent important inflammatory correlates of insomnia. Overall, these findings support the concept that insomnia is associated with a low-grade inflammatory state rather than isolated changes in individual cytokines. Sleep disturbance may contribute to this inflammatory profile through activation of immune signaling pathways, increased oxidative stress, and disruption of neural circuits involved in sleep regulation and emotional processing (61, 62). In contrast, no significant differences were observed for IL-4 and IL-10. This finding may indicate an absence of detectable compensatory anti-inflammatory responses; however, methodological variability and limited statistical power should also be considered.

We further evaluated biomarkers related to neuroplasticity and stress resilience. No significant overall difference was observed for BDNF, whereas circulating NPY levels were significantly reduced in individuals with insomnia. BDNF plays an essential role in synaptic plasticity, neuronal survival, and sleep-dependent neural remodeling, while NPY exerts anxiolytic and stress-buffering effects and may inhibit CRH release (63–65). Reduced circulating NPY levels may reflect altered stress regulation and reduced stress-buffering capacity, potentially contributing to persistent hyperarousal and emotional dysregulation.

### 4.3 Limitations and future perspectives

Despite providing a broad synthesis of circulating biomarkers associated with insomnia, several methodological considerations should be taken into account when interpreting the present findings. According to the GRADE framework, the certainty of evidence ranged from low to very low across all outcomes. This reflects the fact that all included studies were observational in design, which began at a low certainty rating, and were frequently downgraded further because of substantial inconsistency and imprecision. Accordingly, the overall body of evidence should be viewed as suggestive rather than definitive.

The limited and uneven evidence base also constrains the strength of the conclusions. Although this review evaluated 12 circulating biomarkers, only IL-6 was examined in as many as 10 studies, whereas most other biomarkers were supported by considerably fewer studies. In several cases, multiple biomarkers were reported within the same article, meaning that the apparent breadth of biomarkers exceeded the number of truly independent data sources. As a result, some pooled estimates were based on a relatively small amount of evidence and were associated with wide uncertainty. Methodological heterogeneity further complicates interpretation. Differences in participant characteristics, diagnostic criteria, blood sampling protocols, sample type, and laboratory assay platforms likely contributed to between-study variability. In particular, inconsistent sampling times may have influenced circulating biomarker concentrations, especially for markers with circadian variation such as cortisol. The geographic distribution of included studies may limit the generalizability of the findings, as most available evidence was derived from populations in China. Variation in genetic background, environmental exposures, and healthcare settings across populations may therefore have influenced the observed biomarker patterns.

Subgroup analyses were conducted to investigate potential sources of heterogeneity, but substantial residual heterogeneity persisted for several biomarkers. Consequently, pooled estimates should be viewed as representing overall trends rather than precise estimates applicable to all clinical settings. Residual heterogeneity and variability in the available evidence may explain why some biomarkers, particularly CRH and BDNF, were more sensitive to the exclusion of individual studies. Several biomarkers, especially cortisol and CRH, also exhibited substantial between-study heterogeneity, partly driven by large effect sizes reported in individual studies. Nevertheless, sensitivity analyses showed that the overall direction of the pooled associations remained unchanged after excluding these studies, suggesting that the main findings were robust.

In addition to these methodological issues, the biological interpretation of peripheral biomarkers warrants caution. Circulating concentrations may not fully reflect molecular processes within the central nervous system, particularly for neuropeptides and neurotrophic factors. Because most included studies were cross-sectional, the observed associations cannot establish temporal relationships or causality. Whether these biomarker alterations represent predisposing factors, consequences of chronic sleep disturbance, or correlates of related conditions remains uncertain. Future research would benefit from larger, well-characterized longitudinal studies that apply standardized diagnostic criteria, blood sampling procedures, and assay methods. Combining peripheral biomarker assessment with neuroimaging and multi-omics approaches may help clarify how neuroendocrine, inflammatory, and neuroplasticity-related pathways interact across different clinical phenotypes of insomnia, providing a potential basis for more precise biological stratification and targeted interventions.

## 5 Conclusions

This systematic review and meta-analysis suggests that insomnia is associated with alterations in circulating biomarkers involved in neuroendocrine regulation, inflammatory processes, neuroplasticity, and stress resilience. The most consistent findings were elevated cortisol and increased levels of IL-6, TNF-α, and CRP, supporting the potential involvement of HPA axis hyperactivity and low-grade systemic inflammation in insomnia. In addition, reduced circulating NPY levels suggest impaired stress-buffering capacity among individuals with insomnia. Although evidence for CRH and BDNF remains less consistent, the overall biomarker pattern provides further insights into the biological basis of insomnia. Future longitudinal studies are warranted to clarify causal relationships and determine the clinical relevance of circulating biomarkers.

## Data Availability

The original contributions presented in the study are included in the article/**Supplementary Material**, further inquiries can be directed to the corresponding author/s.

## Glossary

SMD: standardized mean difference
CI: confidence interval
IL-6: interleukin-6
TNF-α: tumor necrosis factor-alpha
CRP: C-reactive protein
IL-1β: interleukin-1 beta
IL-2: interleukin-2
IFN-γ: interferon-gamma
IL-4: interleukin-4
IL-10: interleukin-10
NPY: neuropeptide Y
CRH: corticotropin-releasing hormone
BDNF: brain-derived neurotrophic factor
HPA: hypothalamic-pituitary-adrenal
PROSPERO: International Prospective Register of Systematic Reviews
PRISMA: Preferred Reporting Items for Systematic Reviews and Meta-Analyses
MeSH: Medical Subject Headings
CNKI: China National Knowledge Infrastructure
VIP: VIP Database for Chinese Technical Periodicals
CBM: Chinese Biomedical Literature Database
PECOS: Population, Exposure, Comparator, Outcome, and Study Design
SD: standard deviation
AHRQ: Agency for Healthcare Research and Quality
NOS: Newcastle-Ottawa Scale
GRADE: Grading of Recommendations Assessment, Development and Evaluation
DSM-IV: Diagnostic and Statistical Manual of Mental Disorders, Fourth Edition
DSM-5: Diagnostic and Statistical Manual of Mental Disorders, Fifth Edition
ICD-10: International Classification of Diseases, Tenth Revision
ICSD-3: International Classification of Sleep Disorders, Third Edition
CCMD-2-R: Chinese Classification of Mental Disorders, Second Edition, Revised
ELISA: enzyme-linked immunosorbent assay
CLIA: chemiluminescence immunoassay
IG: insomnia group
CG: control group
N: number
NA: not available
m: month
y: years
SP: substance P
ACTH: adrenocorticotropic hormone
TRH: thyrotropin-releasing hormone
TSH: thyroid-stimulating hormone
T3: triiodothyronine
T4: thyroxine
GnRH: gonadotropin-releasing hormone
PSQI: Pittsburgh Sleep Quality Index
hsCRP: high-sensitivity C-reactive protein
S100β: S100 calcium-binding protein B
GFAP: glial fibrillary acidic protein
GDNF: glial cell line-derived neurotrophic factor
ADA: adenosine deaminase
IL-12: interleukin-12
RANTES: regulated upon activation, normal T cell expressed and secreted
SAA: serum amyloid A
GM-CSF: granulocyte-macrophage colony-stimulating factor
ALT: alanine aminotransferase
AST: aspartate aminotransferase
Cr: creatinine
UA: uric acid
LDL: low-density lipoprotein
D-D: D-dimer
Hcy: homocysteine
miR-147: microRNA-147
TGF-β1: transforming growth factor-beta 1
IL-17: interleukin-17
IL-1RA: interleukin-1 receptor antagonist
IL-5: interleukin-5
IL-13: interleukin-13
IL-28A: interleukin-28A
ROS: reactive oxygen species
IL-8: interleukin-8
LPS: lipopolysaccharide
LBP: lipopolysaccharide-binding protein.
