# Supplementary material for: Biomarker evidence for the pathophysiological mechanisms of insomnia: a systematic review and meta-analysis
Source: Front Immunol. 2026 Aug 19;17:1889366. doi: 10.3389/fimmu.2026.1889366 (PMC13533930; doi:10.3389/fimmu.2026.1889366)
Supplement: Supplementary file 1 [file SupplementaryFile1.pdf]

NIHR | National Institute for Health and Care Research

PROSPERO
International prospective register of systematic reviews

Biomarker Evidence for the Pathophysiological Mechanisms of Insomnia: A Systematic Review and Meta-Analysis

Ying Wang, Pei-Chao Wang, Jia-Fu Ji, Xu Feng, Zi-Heng Wang, Tian-Shu Ma, Dong-Liang Diao, Lin Yi

Citation
Ying Wang, Pei-Chao Wang, Jia-Fu Ji, Xu Feng, Zi-Heng Wang, Tian-Shu Ma, Dong-Liang Diao, Lin Yi. Biomarker Evidence for the Pathophysiological Mechanisms of Insomnia: A Systematic Review and Meta-Analysis. PROSPERO 2026 CRD420261396725. Available from https://www.crd.york.ac.uk/PROSPERO/view/CRD420261396725.

REVIEW TITLE AND BASIC DETAILS

Review title
Biomarker Evidence for the Pathophysiological Mechanisms of Insomnia: A Systematic Review and Meta-Analysis

Condition or domain being studied
Insomnia; Inflammatory biomarkers; Inflammation; Biomarker analysis

Rationale for the review
Insomnia is increasingly recognized as a systemic disorder involving persistent hyperarousal, dysregulation of the hypothalamic-pituitary-adrenal (HPA) axis, immune activation, and impaired stress adaptation. Numerous observational studies have investigated circulating biomarkers related to neuroendocrine, inflammatory, neuroplasticity, and stress-resilience pathways in individuals with insomnia. However, findings have been inconsistent, and previous reviews have generally focused on single biomarkers or specific biological pathways, providing only a limited understanding of the systemic biological alterations associated with insomnia. A comprehensive synthesis of circulating biomarkers across multiple biological domains is currently lacking. This systematic review and meta-analysis aims to compare circulating biomarker levels between adults with insomnia and healthy controls, identify the most consistent and robust biological abnormalities, and provide a systems-level characterization of the biological mechanisms underlying insomnia. The findings may help clarify the

pathophysiology of insomnia and identify candidate biomarkers for biological stratification and personalized management.

Review objectives

To systematically review and meta-analyze observational studies comparing circulating biomarker levels between adults with insomnia and healthy controls, and to identify the most consistent and robust alterations in neuroendocrine, inflammatory, neuroplasticity, and stress-resilience biomarkers associated with insomnia.

Keywords

Insomnia; Biomarkers; Inflammation; Systematic review; Meta-analysis

Country

China

ELIGIBILITY CRITERIA

Population

Included

Adults (aged 18 years or older) diagnosed with insomnia according to recognized diagnostic criteria (e.g., DSM-IV, DSM-5, ICD-10, ICSD-3, CCMD-2-R, or validated clinical guidelines) and healthy control participants without insomnia.

Intervention(s) or exposure(s)

Included

Adult patients with insomnia diagnosed using recognized diagnostic criteria (e.g., DSM-IV, DSM-5, ICD-10, ICSD-3, CCMD-2-R, or validated clinical guidelines). Eligible studies must assess circulating biomarkers in blood (serum, plasma, or whole blood) and compare biomarker levels between insomnia patients and healthy controls.

Comparator(s) or control(s)

Included

PICO tags selected: Biomarker analysis

Study design

Only nonrandomized study types will be included.

Context

Observational studies conducted in adult populations in any geographic or healthcare setting (hospital-based, outpatient, or community-based) were eligible, provided that circulating biomarkers were compared between individuals with insomnia and healthy controls.

TIMELINE OF THE REVIEW

Date of first submission to PROSPERO

15 May 2026

Review timeline
Review start date: 1 March 2026. Review end date: 1 September 2026.

Date of registration in PROSPERO
15 May 2026

AVAILABILITY OF FULL PROTOCOL

Availability of full protocol
A full protocol has not been written.

SEARCHING AND SCREENING

Search for unpublished studies
Only published studies will be sought.

Main sources that will be searched
The main sources to be searched are *CNKI – China National Knowledge Infrastructure database*, *Embase* and *PubMed*.

Other sources that will be searched
Web of Science, Cochrane Library, PubMed, EMBASE, China National Knowledge Infrastructure (CNKI), VIP Database for Chinese Technical Periodicals (VIP), Wanfang Data, and Chinese Biomedical Literature Database (CBM)

Search language restrictions
There are no language restrictions.

Search date restrictions
There are no search date restrictions.

Other methods of identifying studies
Other studies will be identified by: *contacting authors or experts*, *reference list checking (backward citation searching)*, *searching conference proceedings* and *searching dissertation and thesis databases*.

Link to search strategy
A full search strategy is available in the full protocol as described in the *Availability of full protocol* section

Selection process
Studies will be screened independently by at least two people (or person/machine combination) with a process to resolve differences.

Other relevant information about searching and screening
None

DATA COLLECTION PROCESS

Data extraction from published articles and reports

Data will be extracted independently by at least two people (or person/machine combination) with a process to resolve differences.

Authors will be asked to provide any required data not available in published reports.

Study risk of bias or quality assessment

Risk of bias will be assessed using: *Newcastle–Ottawa*

Agency for Healthcare Research and Quality

Data will be assessed independently by at least two people (or person/machine combination) with a process to resolve differences.

Additional information will be sought from study investigators if required information is unclear or unavailable in the study publications/reports.

Reporting bias assessment

Risk of bias due to missing results will be assessed

Certainty assessment

The certainty of evidence will be assessed using the Grading of Recommendations, Assessment, Development and Evaluation (GRADE) approach. The evidence for each outcome will be rated as high, moderate, low, or very low certainty based on study limitations, inconsistency, indirectness, imprecision, and publication bias. All included studies are observational in design and will initially be considered low certainty.

OUTCOMES TO BE ANALYSED

Main outcomes

The main outcomes are differences in circulating biomarker levels between adults with insomnia and healthy controls. Biomarkers include neuroendocrine markers, inflammatory cytokines, and neurotrophic factors measured in blood samples The effect size will be expressed as standardized mean differences (SMD) with 95% confidence intervals (CI).

Additional outcomes

There are no additional outcomes.

PLANNED DATA SYNTHESIS

Strategy for data synthesis

Data will be pooled using random–effects or fixed–effect models depending on the level of heterogeneity. Effect sizes will be calculated as standardized mean differences (SMDs) with 95% confidence intervals (CIs) for continuous outcomes. Heterogeneity will be assessed using the I^2 statistic and Cochran's Q test. When substantial heterogeneity is detected (I^2 > 50%), a random–effects model will be applied; otherwise, a fixed–effect model will be used. Subgroup analyses or sensitivity analyses will be performed where appropriate to explore potential sources of heterogeneity. Publication bias will be assessed using funnel plots and Egger's test when sufficient studies are available.

CURRENT REVIEW STAGE

Stage of the review at this submission

Review stage	Started	Completed
Pilot work	✓	
Formal searching/study identification	✓	
Screening search results against inclusion criteria		
Data extraction or receipt of IPD		
Risk of bias/quality assessment		
Data synthesis		

Review status
The review is currently planned or ongoing.

Publication of review results
Results of the review will be published.

REVIEW AFFILIATION, FUNDING AND PEER REVIEW

Review team members
Dr Ying Wang. ORCID: 0009-0001-6790-0236. Changchun University of Chinese Medicine. China.

No conflict of interest declared.

Pei-Chao Wang. Affiliated Hospital of Shandong University of Traditional Chinese Medicine. China.

No conflict of interest declared.

Jia-Fu Ji. Affiliated Hospital of Shandong University of Traditional Chinese Medicine. China.

No conflict of interest declared.

Xu Feng. Changchun University of Chinese Medicine. China.

No conflict of interest declared.

Zi-Heng Wang. Changchun University of Chinese Medicine. China.

No conflict of interest declared.

Tian-Shu Ma. Changchun University of Chinese Medicine. China.

No conflict of interest declared.

Dong-Liang Diao. Affiliated Hospital of Shandong University of Traditional Chinese Medicine. China.

No conflict of interest declared.

Lin Yi (review guarantor and contact) Affiliated Hospital of Shandong University of Traditional Chinese Medicine. China.

No conflict of interest declared.

Named contact

Lin Yi (kiwi100862025@163.com). Affiliated Hospital of Shandong University of Traditional Chinese Medicine. China.

Review affiliation

Changchun University of Chinese Medicine

Funding source

Review has no specific/external funding but is supported by guarantor/review team (non-commercial) institutions.

Peer review

There has been no peer review of this planned review.

ADDITIONAL INFORMATION

Review conflict of interest

Declared individual interests are recorded under team member details.. No additional interests are recorded for this review.

Medical Subject Headings

Sleep Initiation and Maintenance Disorders

SIMILAR REVIEWS

Check for similar records already in PROSPERO

PROSPERO identified a number of existing PROSPERO records that were similar to this one (last check made on 16 May 2026). These are shown below along with the reasons given by that the review team for the reviews being different and/or proceeding.

- Biomarkers of Insomnia: A Systematic Review and Meta-analysis [published 7 March 2025] [CRD420251004792]. **The authors did not check this review**
- Brain-derived neurotrophic factor in insomnia: a systematic review and meta-analysis [published 1 March 2022] [CRD42022307727]. The review was judged **not to be similar**
- Systematic Review and Meta-Analysis of Childhood Insomnia: Diagnostic Criteria, Treatment Strategies, and Research Priorities Through a Delphi Method Approach [published 3 August 2025] [CRD420251118707]. The review was judged **not to be similar**
- Systematic Review of the Pathophysiological Mechanisms of Illness Anxiety Disorder [published 21 March 2022] [CRD42022303294]. The review was judged **not to be similar**

PROSPERO version history

- Version 1.0, published 15 May 2026

Disclaimer

The content of this record displays the information provided by the review team. PROSPERO does not peer review registration records or endorse their content.

PROSPERO accepts and posts the information provided in good faith; responsibility for record content rests with the review team. The guarantor for this record has affirmed that the information provided is truthful and that they understand that deliberate provision of inaccurate information may be construed as scientific misconduct.

PROSPERO does not accept any liability for the content provided in this record or for its use. Readers use the information provided in this record at their own risk.

Any enquiries about the record should be referred to the named review contact
