# Supplementary material for: Biomarker evidence for the pathophysiological mechanisms of insomnia: a systematic review and meta-analysis
Source: Front Immunol. 2026 Aug 19;17:1889366. doi: 10.3389/fimmu.2026.1889366 (PMC13533930; doi:10.3389/fimmu.2026.1889366)
Supplement: Supplementary file 2 [file SupplementaryFile2.docx]

**Contents**

[Availability of code	1](#_Toc1311126223)

[Figure S1. Subgroup analysis of cortisol by country	2](#_Toc768728094)

[Figure S2. Subgroup analysis of cortisol by study design	3](#_Toc751455506)

[Figure S3. Subgroup analysis of cortisol by sample type	4](#_Toc361361335)

[Figure S4. Subgroup analysis of cortisol by assay method	5](#_Toc316203629)

[Figure S5. Subgroup analysis of CRH by sample type	6](#_Toc1559849925)

[Figure S6. Galbraith Plot of Cortisol	7](#_Toc2064810546)

[Figure S7. Galbraith Plot of CRH	7](#_Toc2082594749)

[Figure S8. Subgroup analysis of TNF-α by study design	8](#_Toc333983990)

[Figure S9. Subgroup analysis of TNF-α by sample type	9](#_Toc1894150319)

[Figure S10. Subgroup analysis of TNF-α by assay method	10](#_Toc686828305)

[Figure S11. Subgroup analysis of IL-1β by country	11](#_Toc798719510)

[Figure S12. Subgroup analysis of IL-1β by study design	12](#_Toc158527173)

[Figure S13. Subgroup analysis of IL-1β by sample type	13](#_Toc1486474331)

[Figure S15. Subgroup analysis of IL-2 by sample type	15](#_Toc1496815566)

[Figure S16. Subgroup analysis of IL-2 by assay method	16](#_Toc1355776804)

[Figure S17. Subgroup analysis of IL-4 by study design	17](#_Toc1739250158)

[Figure S18. Subgroup analysis of IL-4 by sample type	18](#_Toc30002542)

[Figure S20. Subgroup analysis of IL-6 by country	20](#_Toc1741549996)

[Figure S21. Subgroup analysis of IL-6 by study design	21](#_Toc28674162)

[Figure S22. Subgroup analysis of IL-6 by sample type	22](#_Toc890303806)

[Figure S23. Subgroup analysis of IL-6 by assay method	23](#_Toc1817498793)

[Figure S24. Subgroup analysis of IL-10 by study design	24](#_Toc894819023)

[Figure S25. Subgroup analysis of IL-10 by sample type	24](#_Toc395339620)

[Figure S27. Subgroup analysis of IFN-γ by study design	26](#_Toc158589522)

[Figure S28. Subgroup analysis of IFN-γ by sample type	27](#_Toc386890327)

[Figure S29. Subgroup analysis of IFN-γ by assay method	28](#_Toc2032726420)

[Figure S30. Subgroup analysis of CRP by study design	29](#_Toc1863084464)

[Figure S31. Subgroup analysis of CRP by sample type	30](#_Toc401529541)

[Figure S33. Galbraith Plot of TNF-α	31](#_Toc1113376713)

[Figure S34. Galbraith Plot of IL-1β	32](#_Toc1497399080)

[Figure S35. Galbraith Plot of IL-2	32](#_Toc425478367)

[Figure S36. Galbraith Plot of IL-4	33](#_Toc2041853306)

[Figure S37. Galbraith Plot of IL-6	33](#_Toc639834882)

[Figure S38. Galbraith Plot of IL-10	34](#_Toc1254241245)

[Figure S39. Galbraith Plot of IFN-γ	34](#_Toc333125763)

[Figure S40. Galbraith Plot of CRP	35](#_Toc354831012)

[Figure S41. Subgroup analysis of BDNF by study design	35](#_Toc82730965)

[Figure S42. Subgroup analysis of NPY by sample type	36](#_Toc1037409146)

[Figure S43. Galbraith Plot of BDNF	36](#_Toc315786829)

[Figure S44. Galbraith Plot of NPY	37](#_Toc997143266)

[Figure S45. Sensitivity analysis of Cortisol	37](#_Toc24490474)

[Figure S46. Sensitivity analysis of CRH	38](#_Toc1442019941)

[Figure S47. Sensitivity analysis of TNF-α	38](#_Toc1676191992)

[Figure S48. Sensitivity analysis of IL-1β	39](#_Toc1068328198)

[Figure S49. Sensitivity analysis of IL-2	39](#_Toc281251219)

[Figure S50. Sensitivity analysis of IL-4	40](#_Toc377730686)

[Figure S51. Sensitivity analysis of IL-6	40](#_Toc557979070)

[Figure S52. Sensitivity analysis of IL-10	41](#_Toc2040626688)

[Figure S53. Sensitivity analysis of IFN-γ	41](#_Toc1498902626)

[Figure S54. Sensitivity analysis of CRP	42](#_Toc2073255872)

[Figure S55. Sensitivity analysis of BDNF	42](#_Toc141784482)

[Figure S56. Sensitivity analysis of NPY	43](#_Toc1412424451)

[Figure S57. Egger’s test for IL-6	43](#_Toc333514019)

[Figure S58. Funnel plot of IL-6	44](#_Toc437798663)

**Availability of code**

#random-effects model

metan n_experimental mean_experimental sd_experimental n_control mean_control sd_control, label(namevar=author, yearvar=year) random

#subgroup analysis

metan n_experimental mean_experimental sd_experimental n_control mean_control sd_control, label(namevar=author, yearvar=year) by(subgroup) random

#funnel plot

metafunnel _ES _seES

#Egger’s test

metabias _ES _seES, egger graph

#sensitivity analysis

metaninf n_experimental mean_experimental sd_experimental n_control mean_control sd_control, label(namevar=author, yearvar=year) random

#Galbraith

meta set _ES _seES

meta galbraith, mlabel(author) random

**Supplementary figures**

**Figure S1.** Subgroup analysis of cortisol by country
*Note.* SMD = standardized mean difference; CI = confidence interval.

**Figure S2.** Subgroup analysis of cortisol by study design
*Note.* SMD = standardized mean difference; CI = confidence interval.

**Figure S3**. Subgroup analysis of cortisol by sample type

*Note.* SMD = standardized mean difference; CI = confidence interval.

**Figure S4.** Subgroup analysis of cortisol by assay method

*Note.* SMD = standardized mean difference; CI = confidence interval.

**Figure S5.** Subgroup analysis of CRH by sample type

*Note.* SMD = standardized mean difference; CI = confidence interval.

**Figure S6.** Galbraith Plot of Cortisol

*Note.* CI = confidence interval.

**Figure S7.** Galbraith Plot of CRH

*Note.* CI = confidence interval.

**Figure S8.** Subgroup analysis of TNF-α by study design
*Note.* SMD = standardized mean difference; CI = confidence interval.

**Figure S9.** Subgroup analysis of TNF-α by sample type
*Note.* SMD = standardized mean difference; CI = confidence interval.

**Figure S10.** Subgroup analysis of TNF-α by assay method
*Note.* SMD = standardized mean difference; CI = confidence interval.

**Figure S11.** Subgroup analysis of IL-1β by country
*Note.* SMD = standardized mean difference; CI = confidence interval.

**Figure S12.** Subgroup analysis of IL-1β by study design
*Note.* SMD = standardized mean difference; CI = confidence interval.

**Figure S13.** Subgroup analysis of IL-1β by sample type
*Note.* SMD = standardized mean difference; CI = confidence interval.

**Figure S14.** Subgroup analysis of IL-1β by assay method
*Note.* SMD = standardized mean difference; CI = confidence interval.

**Figure S15.** Subgroup analysis of IL-2 by sample type
*Note.* SMD = standardized mean difference; CI = confidence interval.

**Figure S16.** Subgroup analysis of IL-2 by assay method
*Note.* SMD = standardized mean difference; CI = confidence interval.

**Figure S17.** Subgroup analysis of IL-4 by study design
*Note.* SMD = standardized mean difference; CI = confidence interval.

**Figure S18.** Subgroup analysis of IL-4 by sample type
*Note.* SMD = standardized mean difference; CI = confidence interval.

**Figure S19.** Subgroup analysis of IL-4 by assay method
*Note.* SMD = standardized mean difference; CI = confidence interval.

**Figure S20**. Subgroup analysis of IL-6 by country
*Note.* SMD = standardized mean difference; CI = confidence interval.

**Figure S21.** Subgroup analysis of IL-6 by study design
*Note.* SMD = standardized mean difference; CI = confidence interval.

**Figure S22.** Subgroup analysis of IL-6 by sample type
*Note.* SMD = standardized mean difference; CI = confidence interval.

**Figure S23.** Subgroup analysis of IL-6 by assay method
*Note.* SMD = standardized mean difference; CI = confidence interval.

**Figure S24.** Subgroup analysis of IL-10 by study design
*Note.* SMD = standardized mean difference; CI = confidence interval.

**Figure S25.** Subgroup analysis of IL-10 by sample type
*Note.* SMD = standardized mean difference; CI = confidence interval.

**Figure S26.** Subgroup analysis of IL-10 by assay method
*Note.* SMD = standardized mean difference; CI = confidence interval.

**Figure S27.** Subgroup analysis of IFN-γ by study design
*Note.* SMD = standardized mean difference; CI = confidence interval.

**Figure S28.** Subgroup analysis of IFN-γ by sample type
*Note.* SMD = standardized mean difference; CI = confidence interval.

**Figure S29.** Subgroup analysis of IFN-γ by assay method
*Note.* SMD = standardized mean difference; CI = confidence interval.

**Figure S30.** Subgroup analysis of CRP by study design
*Note.* SMD = standardized mean difference; CI = confidence interval.

**Figure S31.** Subgroup analysis of CRP by sample type
*Note.* SMD = standardized mean difference; CI = confidence interval.

**Figure S32.** Subgroup analysis of CRP by assay method
*Note.* SMD = standardized mean difference; CI = confidence interval.

**Figure S33.** Galbraith Plot of TNF-α

*Note.* CI = confidence interval.

**Figure S34.** Galbraith Plot of IL-1β

*Note.* CI = confidence interval.

**Figure S35.** Galbraith Plot of IL-2

*Note.* CI = confidence interval.

**Figure S36.** Galbraith Plot of IL-4

*Note.* CI = confidence interval.

**Figure S37.** Galbraith Plot of IL-6

*Note.* CI = confidence interval.

**Figure S38.** Galbraith Plot of IL-10

*Note.* CI = confidence interval.

**Figure S39.** Galbraith Plot of IFN-γ

*Note.* CI = confidence interval.

**Figure S40.** Galbraith Plot of CRP

*Note.* CI = confidence interval.

**Figure S41.** Subgroup analysis of BDNF by study design
*Note.* SMD = standardized mean difference; CI = confidence interval.

**Figure S42.** Subgroup analysis of NPY by sample type
*Note.* SMD = standardized mean difference; CI = confidence interval.

**Figure S43.** Galbraith Plot of BDNF

*Note.* CI = confidence interval.

**Figure S44.** Galbraith Plot of NPY

*Note.* CI = confidence interval.

**Figure S45.** Sensitivity analysis of Cortisol

*Note.* CI = confidence interval.

**Figure S46.** Sensitivity analysis of CRH

*Note.* CI = confidence interval.

**Figure S47.** Sensitivity analysis of TNF-α

*Note.* CI = confidence interval.

**Figure S48**. Sensitivity analysis of IL-1β

*Note.* CI = confidence interval.

**Figure S49.** Sensitivity analysis of IL-2

*Note.* CI = confidence interval.

**Figure S50**. Sensitivity analysis of IL-4

*Note.* CI = confidence interval.

**Figure S51**. Sensitivity analysis of IL-6

*Note.* CI = confidence interval.

**Figure S52.** Sensitivity analysis of IL-10

*Note.* CI = confidence interval.

**Figure S53.** Sensitivity analysis of IFN-γ

*Note.* CI = confidence interval.

**Figure S54.** Sensitivity analysis of CRP

*Note.* CI = confidence interval.

**Figure S55**. Sensitivity analysis of BDNF

*Note.* CI = confidence interval.

**Figure S56**. Sensitivity analysis of NPY

*Note.* CI = confidence interval.

**Figure S57.** Egger’s test for IL-6

*Note.* CI = confidence interval.

**Figure S58.** Funnel plot of IL-6

*Note.* SMD = standardized mean difference.
