# Supplementary material for: Biomarker evidence for the pathophysiological mechanisms of insomnia: a systematic review and meta-analysis
Source: Front Immunol. 2026 Aug 19;17:1889366. doi: 10.3389/fimmu.2026.1889366 (PMC13533930; doi:10.3389/fimmu.2026.1889366)
Supplement: Supplementary file 3 [file SupplementaryFile3.docx]

**Contents**

[Table S1. Search strategy used for Pubmed database	1](#_Toc848139438)

[Table S2. Search strategy used for Web of Science (WOS) database	108](#_Toc1830569327)

[Table S3. Search strategy used for Cochrane Libiary database	115](#_Toc1527951967)

[Table S4. Search strategy used for EMBASE database	122](#_Toc679258543)

[Table S5. Search strategy used for CNKI, Wanfang Data, VIP and CBM database	130](#_Toc275264749)

[Table S6. Risk of bias assessment for studies included in the meta-analysis of IL-6	132](#_Toc694860805)

[Table S7. Assessment of GRADE criteria (risk of bias, inconsistency, imprecision, and publication bias)	133](#_Toc509477249)

**Table S1.** Search strategy used for Pubmed database

| Search number | Query | Search Details | Results |
| --- | --- | --- | --- |
| 93 | #3 AND #92 | ("Sleep Initiation and Maintenance Disorders"[MeSH Terms] OR ("Sleep Initiation and Maintenance Disorders"[Title/Abstract] OR "Disorders of Initiating and Maintaining Sleep"[Title/Abstract] OR "DIMS"[Title/Abstract] OR "Sleeplessness"[Title/Abstract] OR "insomnia disorder"[Title/Abstract] OR "insomnia disorders"[Title/Abstract] OR "Insomnia"[Title/Abstract] OR "Insomnias"[Title/Abstract] OR "chronic insomnia"[Title/Abstract] OR "insomnia chronic"[Title/Abstract] OR "early awakening"[Title/Abstract] OR "awakening early"[Title/Abstract] OR "nonorganic insomnia"[Title/Abstract] OR (("insomnia s"[All Fields] OR "Sleep Initiation and Maintenance Disorders"[MeSH Terms] OR ("Sleep"[All Fields] AND "Initiation"[All Fields] AND "maintenance"[All Fields] AND "Disorders"[All Fields]) OR "Sleep Initiation and Maintenance Disorders"[All Fields] OR "Insomnia"[All Fields] OR "Insomnias"[All Fields]) AND "Nonorganic"[Title/Abstract]) OR "primary insomnia"[Title/Abstract] OR "insomnia primary"[Title/Abstract] OR "psychophysiological insomnia"[Title/Abstract] OR "insomnia psychophysiological"[Title/Abstract] OR "sleep initiation dysfunction"[Title/Abstract] OR (("dysfunctional"[All Fields] OR "dysfunctionals"[All Fields] OR "dysfunctioning"[All Fields] OR "Dysfunctions"[All Fields] OR "physiopathology"[MeSH Subheading] OR "physiopathology"[All Fields] OR "Dysfunction"[All Fields]) AND "sleep initiation"[Title/Abstract]) OR (("dysfunctional"[All Fields] OR "dysfunctionals"[All Fields] OR "dysfunctioning"[All Fields] OR "Dysfunctions"[All Fields] OR "physiopathology"[MeSH Subheading] OR "physiopathology"[All Fields] OR "Dysfunction"[All Fields]) AND "sleep initiation"[Title/Abstract]) OR ((("Sleep"[MeSH Terms] OR "Sleep"[All Fields] OR "sleeping"[All Fields] OR "sleeps"[All Fields] OR "sleep s"[All Fields]) AND ("initial"[All Fields] OR "initially"[All Fields] OR "initials"[All Fields] OR "initiate"[All Fields] OR "initiated"[All Fields] OR "initiates"[All Fields] OR "initiating"[All Fields] OR "Initiation"[All Fields] OR "initiations"[All Fields] OR "initiator"[All Fields] OR "initiators"[All Fields])) AND "Dysfunctions"[Title/Abstract]))) AND ("Serotonin"[MeSH Terms] OR ("Serotonin"[Title/Abstract] OR "5-HT"[Title/Abstract] OR "5-Hydroxytryptamine"[Title/Abstract] OR "5-Hydroxytryptamine"[Title/Abstract] OR "Enteramine"[Title/Abstract] OR "Hippophaine"[Title/Abstract] OR "Hydroxytryptamine"[Title/Abstract]) OR "Dopamine"[MeSH Terms] OR ("Dopamine"[Title/Abstract] OR "DA"[Title/Abstract] OR "3 4 dihydroxyphenethylamine"[Title/Abstract] OR "3 4 dihydroxyphenethylamine"[Title/Abstract] OR "Hydroxytyramine"[Title/Abstract] OR "dopamine hydrochloride"[Title/Abstract] OR "hydrochloride dopamine"[Title/Abstract] OR "Intropin"[Title/Abstract]) OR "Norepinephrine"[MeSH Terms] OR ("Norepinephrine"[Title/Abstract] OR "NE"[Title/Abstract] OR "NA"[Title/Abstract] OR "Levarterenol"[Title/Abstract] OR "Levonorepinephrine"[Title/Abstract] OR "Noradrenaline"[Title/Abstract] OR "Arterenol"[Title/Abstract] OR "norepinephrine hydrochloride"[Title/Abstract] OR "norepinephrine bitartrate"[Title/Abstract] OR "noradrenaline bitartrate"[Title/Abstract] OR "Levonor"[Title/Abstract] OR "Levophed"[Title/Abstract] OR "levophed bitartrate"[Title/Abstract] OR ((("norepinephrin"[All Fields] OR "Norepinephrine"[Supplementary Concept] OR "Norepinephrine"[All Fields] OR "noradrenalin"[All Fields] OR "Norepinephrine"[MeSH Terms] OR "Noradrenaline"[All Fields] OR "norepinephrines"[All Fields]) AND ("tartrates"[Supplementary Concept] OR "tartrates"[All Fields] OR "Tartrate"[All Fields] OR "tartaric acid"[Supplementary Concept] OR "tartaric acid"[All Fields] OR "tartrates"[MeSH Terms])) AND "renaudin"[Title/Abstract])) OR "gamma-Aminobutyric Acid"[MeSH Terms] OR ("gamma-Aminobutyric Acid"[Title/Abstract] OR "GABA"[Title/Abstract] OR "gamma-Aminobutyric Acid"[Title/Abstract] OR "4 aminobutanoic acid"[Title/Abstract] OR "4 aminobutanoic acid"[Title/Abstract] OR "4 aminobutyric acid"[Title/Abstract] OR "4 aminobutyric acid"[Title/Abstract] OR "Aminalon"[Title/Abstract] OR "Aminalone"[Title/Abstract] OR (("gamma-Aminobutyric Acid"[Supplementary Concept] OR "gamma-Aminobutyric Acid"[All Fields] OR "gamma-Aminobutyric Acid"[All Fields] OR "gamma-Aminobutyric Acid"[MeSH Terms] OR ("gamma aminobutyric"[All Fields] AND "Acid"[All Fields]) OR ("gamma"[All Fields] AND "Aminobutyric"[All Fields] AND "Acid"[All Fields])) AND "monolithium salt"[Title/Abstract]) OR (("gamma-Aminobutyric Acid"[Supplementary Concept] OR "gamma-Aminobutyric Acid"[All Fields] OR "gamma-Aminobutyric Acid"[All Fields] OR "gamma-Aminobutyric Acid"[MeSH Terms] OR ("gamma aminobutyric"[All Fields] AND "Acid"[All Fields]) OR ("gamma"[All Fields] AND "Aminobutyric"[All Fields] AND "Acid"[All Fields])) AND "monolithium salt"[Title/Abstract]) OR (("Lithium"[Supplementary Concept] OR "Lithium"[All Fields] OR "Lithium"[MeSH Terms] OR "lithiums"[All Fields]) AND "GABA"[Title/Abstract]) OR (("gamma-Aminobutyric Acid"[Supplementary Concept] OR "gamma-Aminobutyric Acid"[All Fields] OR "GABA"[All Fields] OR "gamma-Aminobutyric Acid"[MeSH Terms] OR ("gamma aminobutyric"[All Fields] AND "Acid"[All Fields])) AND "Lithium"[Title/Abstract]) OR ("gamma aminobutyric"[All Fields] AND "acid hydrochloride"[Title/Abstract]) OR ((("Acids"[Supplementary Concept] OR "Acids"[All Fields] OR "Acid"[All Fields] OR "Acids"[MeSH Terms]) AND ("hydrochlorid"[All Fields] OR "Hydrochloride"[All Fields] OR "hydrochlorides"[All Fields])) AND "gamma aminobutyric"[Title/Abstract]) OR ((("gamma rays"[MeSH Terms] OR ("gamma"[All Fields] AND "rays"[All Fields]) OR "gamma rays"[All Fields] OR "gamma"[All Fields] OR "gamma s"[All Fields] OR "gammae"[All Fields] OR "gammas"[All Fields]) AND ("aminobutyrate"[All Fields] OR "Aminobutyric"[All Fields])) AND "acid hydrochloride"[Title/Abstract]) OR (("hydrochlorid"[All Fields] OR "Hydrochloride"[All Fields] OR "hydrochlorides"[All Fields]) AND "gamma-Aminobutyric Acid"[Title/Abstract]) OR ("gamma aminobutyric"[All Fields] AND "acid monosodium salt"[Title/Abstract]) OR ((("gamma rays"[MeSH Terms] OR ("gamma"[All Fields] AND "rays"[All Fields]) OR "gamma rays"[All Fields] OR "gamma"[All Fields] OR "gamma s"[All Fields] OR "gammae"[All Fields] OR "gammas"[All Fields]) AND ("aminobutyrate"[All Fields] OR "Aminobutyric"[All Fields])) AND "acid monosodium salt"[Title/Abstract]) OR "Gammalon"[Title/Abstract]) OR "Glutamic Acid"[MeSH Terms] OR ("Glutamic Acid"[Title/Abstract] OR "Glutamate"[Title/Abstract]) OR "Acetylcholine"[MeSH Terms] OR ("Acetylcholine"[Title/Abstract] OR "Miochol"[Title/Abstract] OR "acetylcholine bromide"[Title/Abstract] OR "Bromoacetylcholine"[Title/Abstract] OR "acetylcholine chloride"[Title/Abstract] OR "Chloroacetylcholine"[Title/Abstract] OR "acetylcholine iodide"[Title/Abstract] OR (("acetylcholin"[All Fields] OR "Acetylcholine"[Supplementary Concept] OR "Acetylcholine"[All Fields] OR "Acetylcholine"[MeSH Terms] OR "acetylcholines"[All Fields]) AND "Hydroxide"[Title/Abstract]) OR (("acetylcholin"[All Fields] OR "Acetylcholine"[Supplementary Concept] OR "Acetylcholine"[All Fields] OR "Acetylcholine"[MeSH Terms] OR "acetylcholines"[All Fields]) AND "Fluoride"[Title/Abstract]) OR "acetylcholine picrate"[Title/Abstract] OR (("acetylcholin"[All Fields] OR "Acetylcholine"[Supplementary Concept] OR "Acetylcholine"[All Fields] OR "Acetylcholine"[MeSH Terms] OR "acetylcholines"[All Fields]) AND "L-Tartrate"[Title/Abstract]) OR (("acetylcholin"[All Fields] OR "Acetylcholine"[Supplementary Concept] OR "Acetylcholine"[All Fields] OR "Acetylcholine"[MeSH Terms] OR "acetylcholines"[All Fields]) AND "L-Tartrate"[Title/Abstract]) OR "acetylcholine perchlorate"[Title/Abstract] OR ("Acetilcolina"[All Fields] AND "Cusi"[Title/Abstract])) OR "Histamine"[MeSH Terms] OR ("Histamine"[Title/Abstract] OR "Peremin"[Title/Abstract] OR "histamine dihydrochloride"[Title/Abstract] OR "Ceplene"[Title/Abstract] OR "histamine hydrochloride"[Title/Abstract]) OR "Orexins"[MeSH Terms] OR ("Orexins"[Supplementary Concept] OR "Orexins"[All Fields] OR "orexin"[All Fields] OR "hcrt protein human"[Supplementary Concept] OR "hcrt protein human"[All Fields] OR "Orexins"[MeSH Terms] OR ("Orexins"[Supplementary Concept] OR "Orexins"[All Fields] OR "hypocretin"[All Fields] OR "hcrt protein human"[Supplementary Concept] OR "hcrt protein human"[All Fields] OR "Orexins"[MeSH Terms] OR "hypocretins"[All Fields]) OR ("Orexins"[Supplementary Concept] OR "Orexins"[All Fields] OR "orexin"[All Fields] OR "hcrt protein human"[Supplementary Concept] OR "hcrt protein human"[All Fields] OR "Orexins"[MeSH Terms]) OR ("Orexins"[Supplementary Concept] OR "Orexins"[All Fields] OR "hypocretin"[All Fields] OR "hcrt protein human"[Supplementary Concept] OR "hcrt protein human"[All Fields] OR "Orexins"[MeSH Terms] OR "hypocretins"[All Fields]) OR ("Orexins"[Supplementary Concept] OR "Orexins"[All Fields] OR "orexin a"[All Fields] OR "Orexins"[MeSH Terms]) OR ("Orexins"[Supplementary Concept] OR "Orexins"[All Fields] OR "orexin a"[All Fields] OR "Orexins"[MeSH Terms]) OR ("Orexins"[Supplementary Concept] OR "Orexins"[All Fields] OR "hypocretin 1"[All Fields] OR "Orexins"[MeSH Terms]) OR ("Orexins"[Supplementary Concept] OR "Orexins"[All Fields] OR "hypocretin 1"[All Fields] OR "Orexins"[MeSH Terms]) OR ("Orexins"[Supplementary Concept] OR "Orexins"[All Fields] OR "orexin b"[All Fields] OR "Orexins"[MeSH Terms]) OR ("Orexins"[Supplementary Concept] OR "Orexins"[All Fields] OR "orexin b"[All Fields] OR "Orexins"[MeSH Terms]) OR ("Orexins"[Supplementary Concept] OR "Orexins"[All Fields] OR "hypocretin 2"[All Fields] OR "Orexins"[MeSH Terms]) OR ("Orexins"[Supplementary Concept] OR "Orexins"[All Fields] OR "hypocretin 2"[All Fields] OR "Orexins"[MeSH Terms])) OR "Neuropeptide Y"[MeSH Terms] OR ("Neuropeptide Y"[Title/Abstract] OR "neuropeptide tyrosine"[Title/Abstract] OR "neuropeptide y like immunoreactive peptide"[Title/Abstract] OR "neuropeptide y like immunoreactive peptide"[Title/Abstract]) OR "Corticotropin-Releasing Hormone"[MeSH Terms] OR ("Corticotropin-Releasing Hormone"[Title/Abstract] OR "CRH"[Title/Abstract] OR "Corticotropin-Releasing Hormone"[Title/Abstract] OR "corticotropin releasing hormone 41"[Title/Abstract] OR "corticotropin releasing hormone 41"[Title/Abstract] OR "CRF-41"[Title/Abstract] OR "acth releasing hormone"[Title/Abstract] OR "acth releasing hormone"[Title/Abstract] OR "CRF"[Title/Abstract] OR "acth releasing factor"[Title/Abstract] OR "acth releasing factor"[Title/Abstract] OR "Corticoliberin"[Title/Abstract] OR "corticotropin releasing factor"[Title/Abstract] OR "corticotropin releasing factor"[Title/Abstract] OR "corticotropin releasing factor 41"[Title/Abstract] OR "corticotropin releasing factor 41"[Title/Abstract]) OR "Adrenocorticotropic Hormone"[MeSH Terms] OR ("Adrenocorticotropic Hormone"[Title/Abstract] OR "ACTH"[Title/Abstract] OR "Corticotropin"[Title/Abstract] OR (("Adrenocorticotropic Hormone"[Supplementary Concept] OR "Adrenocorticotropic Hormone"[All Fields] OR "Corticotropin"[All Fields] OR "Adrenocorticotropic Hormone"[MeSH Terms] OR ("Adrenocorticotropic"[All Fields] AND "Hormone"[All Fields]) OR "corticotropins"[All Fields]) AND "1-39"[Title/Abstract]) OR (("Adrenocorticotropic Hormone"[Supplementary Concept] OR "Adrenocorticotropic Hormone"[All Fields] OR "Corticotrophin"[All Fields] OR "Adrenocorticotropic Hormone"[MeSH Terms] OR ("Adrenocorticotropic"[All Fields] AND "Hormone"[All Fields]) OR "corticotrophins"[All Fields]) AND "1-39"[Title/Abstract]) OR "adrenocorticotrophic hormone"[Title/Abstract] OR "Adrenocorticotropin"[Title/Abstract] OR "1 39 acth"[Title/Abstract] OR (("Adrenocorticotropic Hormone"[Supplementary Concept] OR "Adrenocorticotropic Hormone"[All Fields] OR "ACTH"[All Fields] OR "Adrenocorticotropic Hormone"[MeSH Terms] OR ("Adrenocorticotropic"[All Fields] AND "Hormone"[All Fields])) AND "1-39"[Title/Abstract]) OR "Corticotrophin"[Title/Abstract]) OR "Hydrocortisone"[MeSH Terms] OR ("Hydrocortisone"[Title/Abstract] OR "Cortisol"[Title/Abstract] OR "Cortril"[Title/Abstract] OR "Epicortisol"[Title/Abstract] OR "11-Epicortisol"[Title/Abstract] OR "11-Epicortisol"[Title/Abstract] OR "Cortef"[Title/Abstract]) OR "Melatonin"[MeSH Terms] OR "Melatonin"[Title/Abstract] OR "Growth Hormone"[MeSH Terms] OR ("Growth Hormone"[Title/Abstract] OR "pituitary growth hormone"[Title/Abstract] OR "Somatotropin"[Title/Abstract] OR (("f8 protein human"[Supplementary Concept] OR "f8 protein human"[All Fields] OR "recombinate"[All Fields] OR "Recombinant"[All Fields] OR "recombinants"[All Fields] OR "recombinated"[All Fields] OR "recombinates"[All Fields] OR "recombination, genetic"[MeSH Terms] OR ("recombination"[All Fields] AND "genetic"[All Fields]) OR "genetic recombination"[All Fields] OR "recombination"[All Fields] OR "recombinations"[All Fields] OR "recombinational"[All Fields] OR "recombinative"[All Fields] OR "recombine"[All Fields] OR "recombined"[All Fields] OR "recombineered"[All Fields] OR "recombineering"[All Fields] OR "recombines"[All Fields] OR "recombining"[All Fields]) AND "pituitary growth hormones"[Title/Abstract]) OR "recombinant growth hormones"[Title/Abstract] OR "recombinant somatotropins"[Title/Abstract] OR "recombinant somatotropin"[Title/Abstract] OR "recombinant growth hormone"[Title/Abstract]) OR "Prolactin"[MeSH Terms] OR ("Prolactin"[Title/Abstract] OR "pituitary lactogenic hormone"[Title/Abstract] OR "pituitary mammotropic hormone"[Title/Abstract] OR "Mammotropin"[Title/Abstract] OR ("PRL"[All Fields] AND "Prolactin"[Title/Abstract])) OR "Leptin"[MeSH Terms] OR ("Leptin"[Title/Abstract] OR "ob gene product"[Title/Abstract] OR "ob protein"[Title/Abstract] OR "obese gene product"[Title/Abstract] OR "obese protein"[Title/Abstract]) OR "Ghrelin"[MeSH Terms] OR ("Ghrelin"[Title/Abstract] OR "appetite regulating hormone"[Title/Abstract] OR "appetite regulating hormone"[Title/Abstract] OR (("gastrics"[All Fields] OR "stomach"[MeSH Terms] OR "stomach"[All Fields] OR "gastric"[All Fields]) AND "MLTRP"[Title/Abstract]) OR "motilin related peptide"[Title/Abstract] OR "motilin related peptide"[Title/Abstract] OR "ghrl protein"[Title/Abstract] OR "PpMTLRP"[Title/Abstract] OR "ghrelin precursor"[Title/Abstract] OR ("Ghrelin-Obestatin"[All Fields] AND "Preprohormone"[Title/Abstract]) OR ((("ghrelins"[All Fields] OR "ghrl protein human"[Supplementary Concept] OR "ghrl protein human"[All Fields] OR "Ghrelin"[All Fields] OR "Ghrelin"[Supplementary Concept] OR "Ghrelin"[MeSH Terms]) AND ("ghrl protein human"[Supplementary Concept] OR "ghrl protein human"[All Fields] OR "Obestatin"[All Fields] OR "Ghrelin"[Supplementary Concept] OR "Ghrelin"[All Fields] OR "Ghrelin"[MeSH Terms])) AND "Preprohormone"[Title/Abstract]) OR ("Motilin-Related"[All Fields] AND "peptide precursor"[Title/Abstract]) OR ((("Motilin"[Supplementary Concept] OR "Motilin"[All Fields] OR "Motilin"[MeSH Terms] OR "motilins"[All Fields]) AND ("family"[MeSH Terms] OR "family"[All Fields] OR "relation"[All Fields] OR "relatability"[All Fields] OR "relatable"[All Fields] OR "Related"[All Fields] OR "relates"[All Fields] OR "relating"[All Fields] OR "relational"[All Fields] OR "relations"[All Fields])) AND "peptide precursor"[Title/Abstract]) OR "Ppghrelin"[Title/Abstract] OR "Obestatin"[Title/Abstract]) OR "Interleukin-6"[MeSH Terms] OR ("Interleukin-6"[Title/Abstract] OR "Interleukin-6"[Title/Abstract] OR "b cell stimulatory factor 2"[Title/Abstract] OR "b cell stimulatory factor 2"[Title/Abstract] OR "b cell differentiation factor"[Title/Abstract] OR "b cell differentiation factor"[Title/Abstract] OR "b cell differentiation factor 2"[Title/Abstract] OR "b cell differentiation factor 2"[Title/Abstract] OR "b cell stimulatory factor 2"[Title/Abstract] OR "b cell stimulatory factor 2"[Title/Abstract] OR "BSF-2"[Title/Abstract] OR "hepatocyte stimulating factor"[Title/Abstract] OR "hepatocyte stimulating factor"[Title/Abstract] OR "hybridoma growth factor"[Title/Abstract] OR "ifn beta 2"[Title/Abstract] OR "IL-6"[Title/Abstract] OR "IL6"[Title/Abstract] OR "interferon beta 2"[Title/Abstract] OR "interferon beta 2"[Title/Abstract] OR "MGI-2"[Title/Abstract] OR "myeloid differentiation inducing protein"[Title/Abstract] OR "myeloid differentiation inducing protein"[Title/Abstract] OR "plasmacytoma growth factor"[Title/Abstract]) OR "Interleukin-1beta"[MeSH Terms] OR ("Interleukin-1beta"[Title/Abstract] OR "Interleukin-1beta"[Title/Abstract] OR "Catabolin"[Title/Abstract] OR "il 1 beta"[Title/Abstract] OR "interleukin 1 beta"[Title/Abstract] OR "interleukin 1 beta"[Title/Abstract]) OR "Tumor Necrosis Factor-alpha"[MeSH Terms] OR ("Tumor Necrosis Factor-alpha"[Title/Abstract] OR "Tumor Necrosis Factor-alpha"[Title/Abstract] OR "TNF-alpha"[Title/Abstract] OR "tumor necrosis factor"[Title/Abstract] OR ((("tumour necrosis factor"[All Fields] OR "Tumor Necrosis Factor-alpha"[Supplementary Concept] OR "Tumor Necrosis Factor-alpha"[All Fields] OR "tumor necrosis factor"[All Fields] OR "Tumor Necrosis Factor-alpha"[MeSH Terms] OR ("Tumor"[All Fields] AND "Necrosis"[All Fields] AND "Factor-alpha"[All Fields]) OR ("Tumor"[All Fields] AND "Necrosis"[All Fields] AND "Factor"[All Fields])) AND ("ligand s"[All Fields] OR "liganded"[All Fields] OR "liganding"[All Fields] OR "ligands"[Supplementary Concept] OR "ligands"[All Fields] OR "ligand"[All Fields] OR "ligands"[MeSH Terms])) AND "superfamily member 2"[Title/Abstract]) OR "TNFalpha"[Title/Abstract] OR "cachectin tumor necrosis factor"[Title/Abstract] OR "cachectin tumor necrosis factor"[Title/Abstract] OR "Cachectin"[Title/Abstract]) OR "Interleukin-17"[MeSH Terms] OR ("Interleukin-17"[Title/Abstract] OR "CTLA-8"[Title/Abstract] OR "IL-17"[Title/Abstract] OR "Interleukin-17"[Title/Abstract] OR "Interleukin-17B"[Title/Abstract] OR "Interleukin-17B"[Title/Abstract] OR "IL-17B"[Title/Abstract] OR "Interleukin-17A"[Title/Abstract] OR "Interleukin-17A"[Title/Abstract] OR "CTLA8"[Title/Abstract] OR "cytotoxic t lymphocyte associated antigen 8"[Title/Abstract] OR "cytotoxic t lymphocyte associated antigen 8"[Title/Abstract] OR "IL-17A"[Title/Abstract] OR "Interleukin-17C"[Title/Abstract] OR "Interleukin-17C"[Title/Abstract] OR (("cytokin"[All Fields] OR "cytokine s"[All Fields] OR "cytokines"[Supplementary Concept] OR "cytokines"[All Fields] OR "Cytokine"[All Fields] OR "cytokines"[MeSH Terms] OR "cytokinic"[All Fields] OR "cytokins"[All Fields]) AND "CX2"[Title/Abstract]) OR "IL-17C"[Title/Abstract] OR "Interleukin-17E"[Title/Abstract] OR "Interleukin-17E"[Title/Abstract] OR "IL-17E"[Title/Abstract] OR "IL-17E"[Title/Abstract] OR "Interleukin-25"[Title/Abstract] OR "Interleukin-25"[Title/Abstract] OR "Interleukin-17F"[Title/Abstract] OR "Interleukin-17F"[Title/Abstract] OR "cytokine ml 1"[Title/Abstract] OR "cytokine ml 1"[Title/Abstract] OR "IL-17F"[Title/Abstract]) OR "Interleukin-8"[MeSH Terms] OR ("Interleukin-8"[Title/Abstract] OR "Interleukin-8"[Title/Abstract] OR "IL-8"[Title/Abstract] OR "IL8"[Title/Abstract] OR "monocyte derived neutrophil chemotactic factor"[Title/Abstract] OR "neutrophil activation factor"[Title/Abstract] OR "lymphocyte derived neutrophil activating peptide"[Title/Abstract] OR "monocyte derived neutrophil activating peptide"[Title/Abstract] OR ((("macrophages, alveolar"[MeSH Terms] OR ("macrophages"[All Fields] AND "alveolar"[All Fields]) OR "alveolar macrophages"[All Fields] OR ("alveolar"[All Fields] AND "macrophage"[All Fields]) OR "alveolar macrophage"[All Fields]) AND ("chemotactant"[All Fields] OR "Chemotactic"[All Fields] OR "chemotactically"[All Fields] OR "chemotactism"[All Fields])) AND "Factor-I"[Title/Abstract]) OR ((("macrophages, alveolar"[MeSH Terms] OR ("macrophages"[All Fields] AND "alveolar"[All Fields]) OR "alveolar macrophages"[All Fields] OR ("alveolar"[All Fields] AND "macrophage"[All Fields]) OR "alveolar macrophage"[All Fields]) AND ("chemotactant"[All Fields] OR "Chemotactic"[All Fields] OR "chemotactically"[All Fields] OR "chemotactism"[All Fields])) AND "Factor-I"[Title/Abstract]) OR "anionic neutrophil activating peptide"[Title/Abstract] OR "anionic neutrophil activating peptide"[Title/Abstract] OR "chemokine cxcl8"[Title/Abstract] OR "cxcl8 chemokines"[Title/Abstract] OR "macrophage derived chemotactic factor"[Title/Abstract] OR "neutrophil chemotactic factor"[Title/Abstract] OR "cxcl8 chemokine"[Title/Abstract] OR "granulocyte chemotactic peptide interleukin 8"[Title/Abstract] OR "granulocyte chemotactic peptide interleukin 8"[Title/Abstract] OR "AMCF-I"[Title/Abstract]) OR "Interleukin-10"[MeSH Terms] OR ("Interleukin-10"[Title/Abstract] OR "Interleukin-10"[Title/Abstract] OR "cytokine synthesis inhibitory factor"[Title/Abstract] OR "IL-10"[Title/Abstract] OR "IL10"[Title/Abstract]) OR "Interferon-gamma"[MeSH Terms] OR ("Interferon-gamma"[Title/Abstract] OR "interferon type ii"[Title/Abstract] OR "immune interferon"[Title/Abstract] OR "type ii interferon"[Title/Abstract] OR "gamma-Interferon"[Title/Abstract]) OR "Transforming Growth Factor beta"[MeSH Terms] OR ("Transforming Growth Factor beta"[Title/Abstract] OR "platelet transforming growth factor"[Title/Abstract] OR "bone derived transforming growth factor"[Title/Abstract] OR "bone derived transforming growth factor"[Title/Abstract] OR "TGF-beta"[Title/Abstract] OR "TGFbeta"[Title/Abstract] OR "milk growth factor"[Title/Abstract]) OR "C-Reactive Protein"[MeSH Terms] OR ("C-Reactive Protein"[Title/Abstract] OR "C-Reactive Protein"[Title/Abstract] OR "high sensitivity c reactive protein"[Title/Abstract] OR "high sensitivity c reactive protein"[Title/Abstract] OR "hs-CRP"[Title/Abstract] OR "hsCRP"[Title/Abstract]) OR "Serum Amyloid A Protein"[MeSH Terms] OR ("Serum Amyloid A Protein"[Title/Abstract] OR "amyloid a precursor"[Title/Abstract] OR "amyloid a protein related serum component"[Title/Abstract] OR "amyloid a protein related serum component"[Title/Abstract] OR (("amyloidogenic proteins"[Supplementary Concept] OR "amyloidogenic proteins"[All Fields] OR "amyloid protein"[All Fields] OR "amyloidogenic proteins"[MeSH Terms] OR ("amyloidogenic"[All Fields] AND "Proteins"[All Fields]) OR ("Amyloid"[All Fields] AND "Protein"[All Fields])) AND "aa precursor"[Title/Abstract]) OR "amyloid protein saa"[Title/Abstract] OR "amyloid serum protein saa"[Title/Abstract] OR (("Amyloid-Related"[All Fields] AND ("blood proteins"[Supplementary Concept] OR "blood proteins"[All Fields] OR "serum protein"[All Fields] OR "blood proteins"[MeSH Terms] OR ("blood"[All Fields] AND "Proteins"[All Fields]) OR ("Serum"[All Fields] AND "Protein"[All Fields]))) AND "SAA"[Title/Abstract]) OR (("Serum"[MeSH Terms] OR "Serum"[All Fields] OR "serums"[All Fields] OR "serum s"[All Fields] OR "serumal"[All Fields]) AND "a related protein"[Title/Abstract]) OR "serum amyloid a"[Title/Abstract] OR "serum amyloid protein a"[Title/Abstract] OR "amyloid a protein"[Title/Abstract] OR "amyloid fibril protein aa"[Title/Abstract] OR "amyloid protein aa"[Title/Abstract] OR "amyloid related serum protein"[Title/Abstract] OR "SAA"[Title/Abstract]) OR "Brain-Derived Neurotrophic Factor"[MeSH Terms] OR ("Brain-Derived Neurotrophic Factor"[Title/Abstract] OR "BDNF"[Title/Abstract] OR "Brain-Derived Neurotrophic Factor"[Title/Abstract]) OR "Nerve Growth Factor"[MeSH Terms] OR ("Nerve Growth Factor"[Title/Abstract] OR "NGF"[Title/Abstract] OR "nerve growth factor 1"[Title/Abstract] OR "nerve growth factor beta subunit"[Title/Abstract] OR "beta nerve growth factor"[Title/Abstract] OR "beta nerve growth factor"[Title/Abstract] OR ("NGF"[All Fields] AND "1beta"[Title/Abstract]) OR "nerve growth factor alpha subunit"[Title/Abstract] OR "nerve growth factor gamma subunit"[Title/Abstract]) OR "Glial Cell Line-Derived Neurotrophic Factor"[MeSH Terms] OR ("Glial Cell Line-Derived Neurotrophic Factor"[Title/Abstract] OR "GDNF"[Title/Abstract] OR "Glial Cell Line-Derived Neurotrophic Factor"[Title/Abstract] OR "Glial Cell Line-Derived Neurotrophic Factor"[Title/Abstract] OR "glial cell derived neurotrophic factor"[Title/Abstract] OR "glial cell derived neurotrophic factor"[Title/Abstract] OR "glial derived neurotrophic factor"[Title/Abstract] OR "glial derived neurotrophic factor"[Title/Abstract] OR "gdnf growth factor"[Title/Abstract]) OR "Malondialdehyde"[MeSH Terms] OR ("Malondialdehyde"[Title/Abstract] OR "MDA"[Title/Abstract] OR "Malonaldehyde"[Title/Abstract] OR "Malonylaldehyde"[Title/Abstract] OR "Malonyldialdehyde"[Title/Abstract] OR "Propanedial"[Title/Abstract] OR "sodium malondialdehyde"[Title/Abstract]) OR "Superoxide Dismutase"[MeSH Terms] OR ("Superoxide Dismutase"[Title/Abstract] OR "SOD"[Title/Abstract] OR "Erythrocuprein"[Title/Abstract] OR "Hemocuprein"[Title/Abstract] OR "cu superoxide dismutase"[Title/Abstract] OR "cu superoxide dismutase"[Title/Abstract] OR "fe zn superoxide dismutase"[Title/Abstract] OR "fe zn superoxide dismutase"[Title/Abstract] OR "cobalt superoxide dismutase"[Title/Abstract] OR "mn superoxide dismutase"[Title/Abstract] OR "mn superoxide dismutase"[Title/Abstract] OR "Mn-SOD"[Title/Abstract] OR "Mn-SOD"[Title/Abstract] OR "manganese superoxide dismutase"[Title/Abstract] OR "ag zn superoxide dismutase"[Title/Abstract] OR "ag zn superoxide dismutase"[Title/Abstract] OR "iron superoxide dismutase"[Title/Abstract] OR "fe superoxide dismutase"[Title/Abstract] OR "fe superoxide dismutase"[Title/Abstract]) OR "Glutathione"[MeSH Terms] OR ("Glutathione"[Title/Abstract] OR "GSH"[Title/Abstract]) OR "Nitric Oxide"[MeSH Terms] OR ("Nitric Oxide"[Title/Abstract] OR "nitrogen monoxide"[Title/Abstract] OR "mononitrogen monoxide"[Title/Abstract] OR "endogenous nitrate vasodilator"[Title/Abstract] OR "endothelium derived nitric oxide"[Title/Abstract]) OR "Insulin"[MeSH Terms] OR ("Insulin"[Title/Abstract] OR "regular insulin"[Title/Abstract] OR "soluble insulin"[Title/Abstract] OR "Iletin"[Title/Abstract] OR "insulin a chain"[Title/Abstract] OR "Novolin"[Title/Abstract] OR "sodium insulin"[Title/Abstract] OR "insulin b chain"[Title/Abstract]) OR "Glucose"[MeSH Terms] OR ("Glucose"[Title/Abstract] OR "D-Glucose"[Title/Abstract] OR "D-Glucose"[Title/Abstract] OR "Dextrose"[Title/Abstract] OR "glucose monohydrate"[Title/Abstract] OR "anhydrous dextrose"[Title/Abstract]) OR "Triglycerides"[MeSH Terms] OR ("Triglycerides"[Title/Abstract] OR "Triglyceride"[Title/Abstract] OR "Triacylglycerols"[Title/Abstract] OR "Triacylglycerol"[Title/Abstract]) OR "lipoproteins, ldl"[MeSH Terms] OR ("Lipoproteins"[Title/Abstract] OR "lipoproteins ldl"[Title/Abstract] OR "LDL"[Title/Abstract] OR "ldl lipoproteins"[Title/Abstract] OR "beta-Lipoprotein"[Title/Abstract] OR "beta-Lipoprotein"[Title/Abstract] OR "low density lipoprotein"[Title/Abstract] OR "low density lipoprotein"[Title/Abstract] OR "beta-Lipoproteins"[Title/Abstract] OR "beta-Lipoproteins"[Title/Abstract] OR "low density lipoproteins"[Title/Abstract] OR "low density lipoproteins"[Title/Abstract] OR "LDL-1"[Title/Abstract] OR (("oxidized low density lipoprotein"[Supplementary Concept] OR "oxidized low density lipoprotein"[All Fields] OR "LDL"[All Fields]) AND "1"[Title/Abstract]) OR "LDL1"[Title/Abstract] OR "low density lipoprotein 1"[Title/Abstract] OR "low density lipoprotein 1"[Title/Abstract] OR "LDL-2"[Title/Abstract] OR (("oxidized low density lipoprotein"[Supplementary Concept] OR "oxidized low density lipoprotein"[All Fields] OR "LDL"[All Fields]) AND "2"[Title/Abstract]) OR "LDL2"[Title/Abstract] OR "low density lipoprotein 2"[Title/Abstract] OR "low density lipoprotein 2"[Title/Abstract]) OR "Amyloid beta-Peptides"[MeSH Terms] OR ("Amyloid beta-Peptides"[Title/Abstract] OR "Amyloid beta-Peptides"[Title/Abstract] OR "alzheimer beta protein"[Title/Abstract] OR "alzheimer beta protein"[Title/Abstract] OR (("alzheime s"[All Fields] OR "alzheimer disease"[MeSH Terms] OR ("Alzheimer"[All Fields] AND "disease"[All Fields]) OR "alzheimer disease"[All Fields] OR "Alzheimer"[All Fields] OR "alzheimer s"[All Fields] OR "alzheimers"[All Fields] OR "alzheimers s"[All Fields]) AND "ABP"[Title/Abstract]) OR (("alzheime s"[All Fields] OR "alzheimer disease"[MeSH Terms] OR ("Alzheimer"[All Fields] AND "disease"[All Fields]) OR "alzheimer disease"[All Fields] OR "Alzheimer"[All Fields] OR "alzheimer s"[All Fields] OR "alzheimers"[All Fields] OR "alzheimers s"[All Fields]) AND "ABP"[Title/Abstract]) OR (("alzheime s"[All Fields] OR "alzheimer disease"[MeSH Terms] OR ("Alzheimer"[All Fields] AND "disease"[All Fields]) OR "alzheimer disease"[All Fields] OR "Alzheimer"[All Fields] OR "alzheimer s"[All Fields] OR "alzheimers"[All Fields] OR "alzheimers s"[All Fields]) AND "ABP"[Title/Abstract]) OR (("alzheime s"[All Fields] OR "alzheimer disease"[MeSH Terms] OR ("Alzheimer"[All Fields] AND "disease"[All Fields]) OR "alzheimer disease"[All Fields] OR "Alzheimer"[All Fields] OR "alzheimer s"[All Fields] OR "alzheimers"[All Fields] OR "alzheimers s"[All Fields]) AND "amyloid fibril protein"[Title/Abstract]) OR (("Amyloid"[Supplementary Concept] OR "Amyloid"[All Fields] OR "amyloids"[All Fields] OR "Amyloid"[MeSH Terms] OR "amyloidal"[All Fields] OR "amyloidic"[All Fields] OR "app protein human"[Supplementary Concept] OR "app protein human"[All Fields]) AND "AD-AP"[Title/Abstract]) OR (("Amyloid"[Supplementary Concept] OR "Amyloid"[All Fields] OR "amyloids"[All Fields] OR "Amyloid"[MeSH Terms] OR "amyloidal"[All Fields] OR "amyloidic"[All Fields] OR "app protein human"[Supplementary Concept] OR "app protein human"[All Fields]) AND "AD-AP"[Title/Abstract]) OR "amyloid beta peptide"[Title/Abstract] OR "amyloid beta peptide"[Title/Abstract] OR "amyloid beta protein"[Title/Abstract] OR "amyloid beta protein"[Title/Abstract] OR "amyloid beta proteins"[Title/Abstract] OR "amyloid beta proteins"[Title/Abstract] OR "amyloid protein a4"[Title/Abstract] OR "beta-Amyloid"[Title/Abstract] OR "beta amyloid protein"[Title/Abstract] OR "beta amyloid protein"[Title/Abstract]) OR "tau Proteins"[MeSH Terms] OR ("tau Proteins"[Title/Abstract] OR "tau protein"[Title/Abstract]) OR "fatty acids, volatile"[MeSH Terms] OR ("fatty acids volatile"[Title/Abstract] OR "volatile fatty acids"[Title/Abstract] OR "short chain fatty acids"[Title/Abstract] OR "short chain fatty acid"[Title/Abstract] OR "short chain fatty acid"[Title/Abstract] OR "volatile fatty acid"[Title/Abstract]) OR "Tryptophan"[MeSH Terms] OR ("Tryptophan"[Title/Abstract] OR "Levotryptophan"[Title/Abstract] OR "L-Tryptophan"[Title/Abstract] OR "L-Tryptophan"[Title/Abstract] OR "tryptophan metabolism alterations"[Title/Abstract] OR "Tryptan"[Title/Abstract] OR "Ardeytropin"[Title/Abstract] OR (("Tryptophan"[Supplementary Concept] OR "Tryptophan"[All Fields] OR "L-Tryptophan"[All Fields] OR "Tryptophan"[MeSH Terms]) AND "ratiopharm"[Title/Abstract]) OR "ratio-Tryptophan"[Title/Abstract] OR "ratio-Tryptophan"[Title/Abstract] OR "Lyphan"[Title/Abstract] OR ("PMS"[All Fields] AND "Tryptophan"[Title/Abstract]) OR "Optimax"[Title/Abstract]) OR "Kynurenine"[MeSH Terms] OR "Kynurenine"[Title/Abstract] OR ("neurotransmit*"[Title/Abstract] OR "hormone*"[Title/Abstract] OR "cytokine*"[Title/Abstract] OR "neurotrophic*"[Title/Abstract] OR "oxidative stress*"[Title/Abstract] OR "metabolic*"[Title/Abstract]) OR ("biomarker*"[Title/Abstract] OR "marker*"[Title/Abstract] OR "parameter*"[Title/Abstract] OR "index*"[Title/Abstract])) | 16,723 |
| 92 | #4 OR #5 OR #6 OR #7 OR #8 OR #9 OR #10 OR #11 OR #12 OR #13 OR #14 OR #15 OR #16 OR #17 OR #18 OR #19 OR #20 OR #21 OR #22 OR #23 OR #24 OR #25 OR #26 OR #27 OR #28 OR #29 OR #30 OR #31 OR #32 OR #33 OR #34 OR #35 OR #36 OR #37 OR #38 OR #39 OR #40 OR #41 OR #42 OR #43 OR #44 OR #45 OR #46 OR #47 OR #48 OR #49 OR #50 OR #51 OR #52 OR #53 OR #54 OR #55 OR #56 OR #57 OR #58 OR #59 OR #60 OR #61 OR #62 OR #63 OR #64 OR #65 OR #66 OR #67 OR #68 OR #69 OR #70 OR #71 OR #72 OR #73 OR #74 OR #75 OR #76 OR #77 OR #78 OR #79 OR #80 OR #81 OR #82 OR #83 OR #84 OR #85 OR #86 OR #87 OR #88 OR #89 OR #90 OR #91 | "Serotonin"[MeSH Terms] OR ("Serotonin"[Title/Abstract] OR "5-HT"[Title/Abstract] OR "5-Hydroxytryptamine"[Title/Abstract] OR "5-Hydroxytryptamine"[Title/Abstract] OR "Enteramine"[Title/Abstract] OR "Hippophaine"[Title/Abstract] OR "Hydroxytryptamine"[Title/Abstract]) OR "Dopamine"[MeSH Terms] OR ("Dopamine"[Title/Abstract] OR "DA"[Title/Abstract] OR "3 4 dihydroxyphenethylamine"[Title/Abstract] OR "3 4 dihydroxyphenethylamine"[Title/Abstract] OR "Hydroxytyramine"[Title/Abstract] OR "dopamine hydrochloride"[Title/Abstract] OR "hydrochloride dopamine"[Title/Abstract] OR "Intropin"[Title/Abstract]) OR "Norepinephrine"[MeSH Terms] OR ("Norepinephrine"[Title/Abstract] OR "NE"[Title/Abstract] OR "NA"[Title/Abstract] OR "Levarterenol"[Title/Abstract] OR "Levonorepinephrine"[Title/Abstract] OR "Noradrenaline"[Title/Abstract] OR "Arterenol"[Title/Abstract] OR "norepinephrine hydrochloride"[Title/Abstract] OR "norepinephrine bitartrate"[Title/Abstract] OR "noradrenaline bitartrate"[Title/Abstract] OR "Levonor"[Title/Abstract] OR "Levophed"[Title/Abstract] OR "levophed bitartrate"[Title/Abstract] OR ((("norepinephrin"[All Fields] OR "Norepinephrine"[Supplementary Concept] OR "Norepinephrine"[All Fields] OR "noradrenalin"[All Fields] OR "Norepinephrine"[MeSH Terms] OR "Noradrenaline"[All Fields] OR "norepinephrines"[All Fields]) AND ("tartrates"[Supplementary Concept] OR "tartrates"[All Fields] OR "Tartrate"[All Fields] OR "tartaric acid"[Supplementary Concept] OR "tartaric acid"[All Fields] OR "tartrates"[MeSH Terms])) AND "renaudin"[Title/Abstract])) OR "gamma-Aminobutyric Acid"[MeSH Terms] OR ("gamma-Aminobutyric Acid"[Title/Abstract] OR "GABA"[Title/Abstract] OR "gamma-Aminobutyric Acid"[Title/Abstract] OR "4 aminobutanoic acid"[Title/Abstract] OR "4 aminobutanoic acid"[Title/Abstract] OR "4 aminobutyric acid"[Title/Abstract] OR "4 aminobutyric acid"[Title/Abstract] OR "Aminalon"[Title/Abstract] OR "Aminalone"[Title/Abstract] OR (("gamma-Aminobutyric Acid"[Supplementary Concept] OR "gamma-Aminobutyric Acid"[All Fields] OR "gamma-Aminobutyric Acid"[All Fields] OR "gamma-Aminobutyric Acid"[MeSH Terms] OR ("gamma aminobutyric"[All Fields] AND "Acid"[All Fields]) OR ("gamma"[All Fields] AND "Aminobutyric"[All Fields] AND "Acid"[All Fields])) AND "monolithium salt"[Title/Abstract]) OR (("gamma-Aminobutyric Acid"[Supplementary Concept] OR "gamma-Aminobutyric Acid"[All Fields] OR "gamma-Aminobutyric Acid"[All Fields] OR "gamma-Aminobutyric Acid"[MeSH Terms] OR ("gamma aminobutyric"[All Fields] AND "Acid"[All Fields]) OR ("gamma"[All Fields] AND "Aminobutyric"[All Fields] AND "Acid"[All Fields])) AND "monolithium salt"[Title/Abstract]) OR (("Lithium"[Supplementary Concept] OR "Lithium"[All Fields] OR "Lithium"[MeSH Terms] OR "lithiums"[All Fields]) AND "GABA"[Title/Abstract]) OR (("gamma-Aminobutyric Acid"[Supplementary Concept] OR "gamma-Aminobutyric Acid"[All Fields] OR "GABA"[All Fields] OR "gamma-Aminobutyric Acid"[MeSH Terms] OR ("gamma aminobutyric"[All Fields] AND "Acid"[All Fields])) AND "Lithium"[Title/Abstract]) OR ("gamma aminobutyric"[All Fields] AND "acid hydrochloride"[Title/Abstract]) OR ((("Acids"[Supplementary Concept] OR "Acids"[All Fields] OR "Acid"[All Fields] OR "Acids"[MeSH Terms]) AND ("hydrochlorid"[All Fields] OR "Hydrochloride"[All Fields] OR "hydrochlorides"[All Fields])) AND "gamma aminobutyric"[Title/Abstract]) OR ((("gamma rays"[MeSH Terms] OR ("gamma"[All Fields] AND "rays"[All Fields]) OR "gamma rays"[All Fields] OR "gamma"[All Fields] OR "gamma s"[All Fields] OR "gammae"[All Fields] OR "gammas"[All Fields]) AND ("aminobutyrate"[All Fields] OR "Aminobutyric"[All Fields])) AND "acid hydrochloride"[Title/Abstract]) OR (("hydrochlorid"[All Fields] OR "Hydrochloride"[All Fields] OR "hydrochlorides"[All Fields]) AND "gamma-Aminobutyric Acid"[Title/Abstract]) OR ("gamma aminobutyric"[All Fields] AND "acid monosodium salt"[Title/Abstract]) OR ((("gamma rays"[MeSH Terms] OR ("gamma"[All Fields] AND "rays"[All Fields]) OR "gamma rays"[All Fields] OR "gamma"[All Fields] OR "gamma s"[All Fields] OR "gammae"[All Fields] OR "gammas"[All Fields]) AND ("aminobutyrate"[All Fields] OR "Aminobutyric"[All Fields])) AND "acid monosodium salt"[Title/Abstract]) OR "Gammalon"[Title/Abstract]) OR "Glutamic Acid"[MeSH Terms] OR ("Glutamic Acid"[Title/Abstract] OR "Glutamate"[Title/Abstract]) OR "Acetylcholine"[MeSH Terms] OR ("Acetylcholine"[Title/Abstract] OR "Miochol"[Title/Abstract] OR "acetylcholine bromide"[Title/Abstract] OR "Bromoacetylcholine"[Title/Abstract] OR "acetylcholine chloride"[Title/Abstract] OR "Chloroacetylcholine"[Title/Abstract] OR "acetylcholine iodide"[Title/Abstract] OR (("acetylcholin"[All Fields] OR "Acetylcholine"[Supplementary Concept] OR "Acetylcholine"[All Fields] OR "Acetylcholine"[MeSH Terms] OR "acetylcholines"[All Fields]) AND "Hydroxide"[Title/Abstract]) OR (("acetylcholin"[All Fields] OR "Acetylcholine"[Supplementary Concept] OR "Acetylcholine"[All Fields] OR "Acetylcholine"[MeSH Terms] OR "acetylcholines"[All Fields]) AND "Fluoride"[Title/Abstract]) OR "acetylcholine picrate"[Title/Abstract] OR (("acetylcholin"[All Fields] OR "Acetylcholine"[Supplementary Concept] OR "Acetylcholine"[All Fields] OR "Acetylcholine"[MeSH Terms] OR "acetylcholines"[All Fields]) AND "L-Tartrate"[Title/Abstract]) OR (("acetylcholin"[All Fields] OR "Acetylcholine"[Supplementary Concept] OR "Acetylcholine"[All Fields] OR "Acetylcholine"[MeSH Terms] OR "acetylcholines"[All Fields]) AND "L-Tartrate"[Title/Abstract]) OR "acetylcholine perchlorate"[Title/Abstract] OR ("Acetilcolina"[All Fields] AND "Cusi"[Title/Abstract])) OR "Histamine"[MeSH Terms] OR ("Histamine"[Title/Abstract] OR "Peremin"[Title/Abstract] OR "histamine dihydrochloride"[Title/Abstract] OR "Ceplene"[Title/Abstract] OR "histamine hydrochloride"[Title/Abstract]) OR "Orexins"[MeSH Terms] OR ("Orexins"[Supplementary Concept] OR "Orexins"[All Fields] OR "orexin"[All Fields] OR "hcrt protein human"[Supplementary Concept] OR "hcrt protein human"[All Fields] OR "Orexins"[MeSH Terms] OR ("Orexins"[Supplementary Concept] OR "Orexins"[All Fields] OR "hypocretin"[All Fields] OR "hcrt protein human"[Supplementary Concept] OR "hcrt protein human"[All Fields] OR "Orexins"[MeSH Terms] OR "hypocretins"[All Fields]) OR ("Orexins"[Supplementary Concept] OR "Orexins"[All Fields] OR "orexin"[All Fields] OR "hcrt protein human"[Supplementary Concept] OR "hcrt protein human"[All Fields] OR "Orexins"[MeSH Terms]) OR ("Orexins"[Supplementary Concept] OR "Orexins"[All Fields] OR "hypocretin"[All Fields] OR "hcrt protein human"[Supplementary Concept] OR "hcrt protein human"[All Fields] OR "Orexins"[MeSH Terms] OR "hypocretins"[All Fields]) OR ("Orexins"[Supplementary Concept] OR "Orexins"[All Fields] OR "orexin a"[All Fields] OR "Orexins"[MeSH Terms]) OR ("Orexins"[Supplementary Concept] OR "Orexins"[All Fields] OR "orexin a"[All Fields] OR "Orexins"[MeSH Terms]) OR ("Orexins"[Supplementary Concept] OR "Orexins"[All Fields] OR "hypocretin 1"[All Fields] OR "Orexins"[MeSH Terms]) OR ("Orexins"[Supplementary Concept] OR "Orexins"[All Fields] OR "hypocretin 1"[All Fields] OR "Orexins"[MeSH Terms]) OR ("Orexins"[Supplementary Concept] OR "Orexins"[All Fields] OR "orexin b"[All Fields] OR "Orexins"[MeSH Terms]) OR ("Orexins"[Supplementary Concept] OR "Orexins"[All Fields] OR "orexin b"[All Fields] OR "Orexins"[MeSH Terms]) OR ("Orexins"[Supplementary Concept] OR "Orexins"[All Fields] OR "hypocretin 2"[All Fields] OR "Orexins"[MeSH Terms]) OR ("Orexins"[Supplementary Concept] OR "Orexins"[All Fields] OR "hypocretin 2"[All Fields] OR "Orexins"[MeSH Terms])) OR "Neuropeptide Y"[MeSH Terms] OR ("Neuropeptide Y"[Title/Abstract] OR "neuropeptide tyrosine"[Title/Abstract] OR "neuropeptide y like immunoreactive peptide"[Title/Abstract] OR "neuropeptide y like immunoreactive peptide"[Title/Abstract]) OR "Corticotropin-Releasing Hormone"[MeSH Terms] OR ("Corticotropin-Releasing Hormone"[Title/Abstract] OR "CRH"[Title/Abstract] OR "Corticotropin-Releasing Hormone"[Title/Abstract] OR "corticotropin releasing hormone 41"[Title/Abstract] OR "corticotropin releasing hormone 41"[Title/Abstract] OR "CRF-41"[Title/Abstract] OR "acth releasing hormone"[Title/Abstract] OR "acth releasing hormone"[Title/Abstract] OR "CRF"[Title/Abstract] OR "acth releasing factor"[Title/Abstract] OR "acth releasing factor"[Title/Abstract] OR "Corticoliberin"[Title/Abstract] OR "corticotropin releasing factor"[Title/Abstract] OR "corticotropin releasing factor"[Title/Abstract] OR "corticotropin releasing factor 41"[Title/Abstract] OR "corticotropin releasing factor 41"[Title/Abstract]) OR "Adrenocorticotropic Hormone"[MeSH Terms] OR ("Adrenocorticotropic Hormone"[Title/Abstract] OR "ACTH"[Title/Abstract] OR "Corticotropin"[Title/Abstract] OR (("Adrenocorticotropic Hormone"[Supplementary Concept] OR "Adrenocorticotropic Hormone"[All Fields] OR "Corticotropin"[All Fields] OR "Adrenocorticotropic Hormone"[MeSH Terms] OR ("Adrenocorticotropic"[All Fields] AND "Hormone"[All Fields]) OR "corticotropins"[All Fields]) AND "1-39"[Title/Abstract]) OR (("Adrenocorticotropic Hormone"[Supplementary Concept] OR "Adrenocorticotropic Hormone"[All Fields] OR "Corticotrophin"[All Fields] OR "Adrenocorticotropic Hormone"[MeSH Terms] OR ("Adrenocorticotropic"[All Fields] AND "Hormone"[All Fields]) OR "corticotrophins"[All Fields]) AND "1-39"[Title/Abstract]) OR "adrenocorticotrophic hormone"[Title/Abstract] OR "Adrenocorticotropin"[Title/Abstract] OR "1 39 acth"[Title/Abstract] OR (("Adrenocorticotropic Hormone"[Supplementary Concept] OR "Adrenocorticotropic Hormone"[All Fields] OR "ACTH"[All Fields] OR "Adrenocorticotropic Hormone"[MeSH Terms] OR ("Adrenocorticotropic"[All Fields] AND "Hormone"[All Fields])) AND "1-39"[Title/Abstract]) OR "Corticotrophin"[Title/Abstract]) OR "Hydrocortisone"[MeSH Terms] OR ("Hydrocortisone"[Title/Abstract] OR "Cortisol"[Title/Abstract] OR "Cortril"[Title/Abstract] OR "Epicortisol"[Title/Abstract] OR "11-Epicortisol"[Title/Abstract] OR "11-Epicortisol"[Title/Abstract] OR "Cortef"[Title/Abstract]) OR "Melatonin"[MeSH Terms] OR "Melatonin"[Title/Abstract] OR "Growth Hormone"[MeSH Terms] OR ("Growth Hormone"[Title/Abstract] OR "pituitary growth hormone"[Title/Abstract] OR "Somatotropin"[Title/Abstract] OR (("f8 protein human"[Supplementary Concept] OR "f8 protein human"[All Fields] OR "recombinate"[All Fields] OR "Recombinant"[All Fields] OR "recombinants"[All Fields] OR "recombinated"[All Fields] OR "recombinates"[All Fields] OR "recombination, genetic"[MeSH Terms] OR ("recombination"[All Fields] AND "genetic"[All Fields]) OR "genetic recombination"[All Fields] OR "recombination"[All Fields] OR "recombinations"[All Fields] OR "recombinational"[All Fields] OR "recombinative"[All Fields] OR "recombine"[All Fields] OR "recombined"[All Fields] OR "recombineered"[All Fields] OR "recombineering"[All Fields] OR "recombines"[All Fields] OR "recombining"[All Fields]) AND "pituitary growth hormones"[Title/Abstract]) OR "recombinant growth hormones"[Title/Abstract] OR "recombinant somatotropins"[Title/Abstract] OR "recombinant somatotropin"[Title/Abstract] OR "recombinant growth hormone"[Title/Abstract]) OR "Prolactin"[MeSH Terms] OR ("Prolactin"[Title/Abstract] OR "pituitary lactogenic hormone"[Title/Abstract] OR "pituitary mammotropic hormone"[Title/Abstract] OR "Mammotropin"[Title/Abstract] OR ("PRL"[All Fields] AND "Prolactin"[Title/Abstract])) OR "Leptin"[MeSH Terms] OR ("Leptin"[Title/Abstract] OR "ob gene product"[Title/Abstract] OR "ob protein"[Title/Abstract] OR "obese gene product"[Title/Abstract] OR "obese protein"[Title/Abstract]) OR "Ghrelin"[MeSH Terms] OR ("Ghrelin"[Title/Abstract] OR "appetite regulating hormone"[Title/Abstract] OR "appetite regulating hormone"[Title/Abstract] OR (("gastrics"[All Fields] OR "stomach"[MeSH Terms] OR "stomach"[All Fields] OR "gastric"[All Fields]) AND "MLTRP"[Title/Abstract]) OR "motilin related peptide"[Title/Abstract] OR "motilin related peptide"[Title/Abstract] OR "ghrl protein"[Title/Abstract] OR "PpMTLRP"[Title/Abstract] OR "ghrelin precursor"[Title/Abstract] OR ("Ghrelin-Obestatin"[All Fields] AND "Preprohormone"[Title/Abstract]) OR ((("ghrelins"[All Fields] OR "ghrl protein human"[Supplementary Concept] OR "ghrl protein human"[All Fields] OR "Ghrelin"[All Fields] OR "Ghrelin"[Supplementary Concept] OR "Ghrelin"[MeSH Terms]) AND ("ghrl protein human"[Supplementary Concept] OR "ghrl protein human"[All Fields] OR "Obestatin"[All Fields] OR "Ghrelin"[Supplementary Concept] OR "Ghrelin"[All Fields] OR "Ghrelin"[MeSH Terms])) AND "Preprohormone"[Title/Abstract]) OR ("Motilin-Related"[All Fields] AND "peptide precursor"[Title/Abstract]) OR ((("Motilin"[Supplementary Concept] OR "Motilin"[All Fields] OR "Motilin"[MeSH Terms] OR "motilins"[All Fields]) AND ("family"[MeSH Terms] OR "family"[All Fields] OR "relation"[All Fields] OR "relatability"[All Fields] OR "relatable"[All Fields] OR "Related"[All Fields] OR "relates"[All Fields] OR "relating"[All Fields] OR "relational"[All Fields] OR "relations"[All Fields])) AND "peptide precursor"[Title/Abstract]) OR "Ppghrelin"[Title/Abstract] OR "Obestatin"[Title/Abstract]) OR "Interleukin-6"[MeSH Terms] OR ("Interleukin-6"[Title/Abstract] OR "Interleukin-6"[Title/Abstract] OR "b cell stimulatory factor 2"[Title/Abstract] OR "b cell stimulatory factor 2"[Title/Abstract] OR "b cell differentiation factor"[Title/Abstract] OR "b cell differentiation factor"[Title/Abstract] OR "b cell differentiation factor 2"[Title/Abstract] OR "b cell differentiation factor 2"[Title/Abstract] OR "b cell stimulatory factor 2"[Title/Abstract] OR "b cell stimulatory factor 2"[Title/Abstract] OR "BSF-2"[Title/Abstract] OR "hepatocyte stimulating factor"[Title/Abstract] OR "hepatocyte stimulating factor"[Title/Abstract] OR "hybridoma growth factor"[Title/Abstract] OR "ifn beta 2"[Title/Abstract] OR "IL-6"[Title/Abstract] OR "IL6"[Title/Abstract] OR "interferon beta 2"[Title/Abstract] OR "interferon beta 2"[Title/Abstract] OR "MGI-2"[Title/Abstract] OR "myeloid differentiation inducing protein"[Title/Abstract] OR "myeloid differentiation inducing protein"[Title/Abstract] OR "plasmacytoma growth factor"[Title/Abstract]) OR "Interleukin-1beta"[MeSH Terms] OR ("Interleukin-1beta"[Title/Abstract] OR "Interleukin-1beta"[Title/Abstract] OR "Catabolin"[Title/Abstract] OR "il 1 beta"[Title/Abstract] OR "interleukin 1 beta"[Title/Abstract] OR "interleukin 1 beta"[Title/Abstract]) OR "Tumor Necrosis Factor-alpha"[MeSH Terms] OR ("Tumor Necrosis Factor-alpha"[Title/Abstract] OR "Tumor Necrosis Factor-alpha"[Title/Abstract] OR "TNF-alpha"[Title/Abstract] OR "tumor necrosis factor"[Title/Abstract] OR ((("tumour necrosis factor"[All Fields] OR "Tumor Necrosis Factor-alpha"[Supplementary Concept] OR "Tumor Necrosis Factor-alpha"[All Fields] OR "tumor necrosis factor"[All Fields] OR "Tumor Necrosis Factor-alpha"[MeSH Terms] OR ("Tumor"[All Fields] AND "Necrosis"[All Fields] AND "Factor-alpha"[All Fields]) OR ("Tumor"[All Fields] AND "Necrosis"[All Fields] AND "Factor"[All Fields])) AND ("ligand s"[All Fields] OR "liganded"[All Fields] OR "liganding"[All Fields] OR "ligands"[Supplementary Concept] OR "ligands"[All Fields] OR "ligand"[All Fields] OR "ligands"[MeSH Terms])) AND "superfamily member 2"[Title/Abstract]) OR "TNFalpha"[Title/Abstract] OR "cachectin tumor necrosis factor"[Title/Abstract] OR "cachectin tumor necrosis factor"[Title/Abstract] OR "Cachectin"[Title/Abstract]) OR "Interleukin-17"[MeSH Terms] OR ("Interleukin-17"[Title/Abstract] OR "CTLA-8"[Title/Abstract] OR "IL-17"[Title/Abstract] OR "Interleukin-17"[Title/Abstract] OR "Interleukin-17B"[Title/Abstract] OR "Interleukin-17B"[Title/Abstract] OR "IL-17B"[Title/Abstract] OR "Interleukin-17A"[Title/Abstract] OR "Interleukin-17A"[Title/Abstract] OR "CTLA8"[Title/Abstract] OR "cytotoxic t lymphocyte associated antigen 8"[Title/Abstract] OR "cytotoxic t lymphocyte associated antigen 8"[Title/Abstract] OR "IL-17A"[Title/Abstract] OR "Interleukin-17C"[Title/Abstract] OR "Interleukin-17C"[Title/Abstract] OR (("cytokin"[All Fields] OR "cytokine s"[All Fields] OR "cytokines"[Supplementary Concept] OR "cytokines"[All Fields] OR "Cytokine"[All Fields] OR "cytokines"[MeSH Terms] OR "cytokinic"[All Fields] OR "cytokins"[All Fields]) AND "CX2"[Title/Abstract]) OR "IL-17C"[Title/Abstract] OR "Interleukin-17E"[Title/Abstract] OR "Interleukin-17E"[Title/Abstract] OR "IL-17E"[Title/Abstract] OR "IL-17E"[Title/Abstract] OR "Interleukin-25"[Title/Abstract] OR "Interleukin-25"[Title/Abstract] OR "Interleukin-17F"[Title/Abstract] OR "Interleukin-17F"[Title/Abstract] OR "cytokine ml 1"[Title/Abstract] OR "cytokine ml 1"[Title/Abstract] OR "IL-17F"[Title/Abstract]) OR "Interleukin-8"[MeSH Terms] OR ("Interleukin-8"[Title/Abstract] OR "Interleukin-8"[Title/Abstract] OR "IL-8"[Title/Abstract] OR "IL8"[Title/Abstract] OR "monocyte derived neutrophil chemotactic factor"[Title/Abstract] OR "neutrophil activation factor"[Title/Abstract] OR "lymphocyte derived neutrophil activating peptide"[Title/Abstract] OR "monocyte derived neutrophil activating peptide"[Title/Abstract] OR ((("macrophages, alveolar"[MeSH Terms] OR ("macrophages"[All Fields] AND "alveolar"[All Fields]) OR "alveolar macrophages"[All Fields] OR ("alveolar"[All Fields] AND "macrophage"[All Fields]) OR "alveolar macrophage"[All Fields]) AND ("chemotactant"[All Fields] OR "Chemotactic"[All Fields] OR "chemotactically"[All Fields] OR "chemotactism"[All Fields])) AND "Factor-I"[Title/Abstract]) OR ((("macrophages, alveolar"[MeSH Terms] OR ("macrophages"[All Fields] AND "alveolar"[All Fields]) OR "alveolar macrophages"[All Fields] OR ("alveolar"[All Fields] AND "macrophage"[All Fields]) OR "alveolar macrophage"[All Fields]) AND ("chemotactant"[All Fields] OR "Chemotactic"[All Fields] OR "chemotactically"[All Fields] OR "chemotactism"[All Fields])) AND "Factor-I"[Title/Abstract]) OR "anionic neutrophil activating peptide"[Title/Abstract] OR "anionic neutrophil activating peptide"[Title/Abstract] OR "chemokine cxcl8"[Title/Abstract] OR "cxcl8 chemokines"[Title/Abstract] OR "macrophage derived chemotactic factor"[Title/Abstract] OR "neutrophil chemotactic factor"[Title/Abstract] OR "cxcl8 chemokine"[Title/Abstract] OR "granulocyte chemotactic peptide interleukin 8"[Title/Abstract] OR "granulocyte chemotactic peptide interleukin 8"[Title/Abstract] OR "AMCF-I"[Title/Abstract]) OR "Interleukin-10"[MeSH Terms] OR ("Interleukin-10"[Title/Abstract] OR "Interleukin-10"[Title/Abstract] OR "cytokine synthesis inhibitory factor"[Title/Abstract] OR "IL-10"[Title/Abstract] OR "IL10"[Title/Abstract]) OR "Interferon-gamma"[MeSH Terms] OR ("Interferon-gamma"[Title/Abstract] OR "interferon type ii"[Title/Abstract] OR "immune interferon"[Title/Abstract] OR "type ii interferon"[Title/Abstract] OR "gamma-Interferon"[Title/Abstract]) OR "Transforming Growth Factor beta"[MeSH Terms] OR ("Transforming Growth Factor beta"[Title/Abstract] OR "platelet transforming growth factor"[Title/Abstract] OR "bone derived transforming growth factor"[Title/Abstract] OR "bone derived transforming growth factor"[Title/Abstract] OR "TGF-beta"[Title/Abstract] OR "TGFbeta"[Title/Abstract] OR "milk growth factor"[Title/Abstract]) OR "C-Reactive Protein"[MeSH Terms] OR ("C-Reactive Protein"[Title/Abstract] OR "C-Reactive Protein"[Title/Abstract] OR "high sensitivity c reactive protein"[Title/Abstract] OR "high sensitivity c reactive protein"[Title/Abstract] OR "hs-CRP"[Title/Abstract] OR "hsCRP"[Title/Abstract]) OR "Serum Amyloid A Protein"[MeSH Terms] OR ("Serum Amyloid A Protein"[Title/Abstract] OR "amyloid a precursor"[Title/Abstract] OR "amyloid a protein related serum component"[Title/Abstract] OR "amyloid a protein related serum component"[Title/Abstract] OR (("amyloidogenic proteins"[Supplementary Concept] OR "amyloidogenic proteins"[All Fields] OR "amyloid protein"[All Fields] OR "amyloidogenic proteins"[MeSH Terms] OR ("amyloidogenic"[All Fields] AND "Proteins"[All Fields]) OR ("Amyloid"[All Fields] AND "Protein"[All Fields])) AND "aa precursor"[Title/Abstract]) OR "amyloid protein saa"[Title/Abstract] OR "amyloid serum protein saa"[Title/Abstract] OR (("Amyloid-Related"[All Fields] AND ("blood proteins"[Supplementary Concept] OR "blood proteins"[All Fields] OR "serum protein"[All Fields] OR "blood proteins"[MeSH Terms] OR ("blood"[All Fields] AND "Proteins"[All Fields]) OR ("Serum"[All Fields] AND "Protein"[All Fields]))) AND "SAA"[Title/Abstract]) OR (("Serum"[MeSH Terms] OR "Serum"[All Fields] OR "serums"[All Fields] OR "serum s"[All Fields] OR "serumal"[All Fields]) AND "a related protein"[Title/Abstract]) OR "serum amyloid a"[Title/Abstract] OR "serum amyloid protein a"[Title/Abstract] OR "amyloid a protein"[Title/Abstract] OR "amyloid fibril protein aa"[Title/Abstract] OR "amyloid protein aa"[Title/Abstract] OR "amyloid related serum protein"[Title/Abstract] OR "SAA"[Title/Abstract]) OR "Brain-Derived Neurotrophic Factor"[MeSH Terms] OR ("Brain-Derived Neurotrophic Factor"[Title/Abstract] OR "BDNF"[Title/Abstract] OR "Brain-Derived Neurotrophic Factor"[Title/Abstract]) OR "Nerve Growth Factor"[MeSH Terms] OR ("Nerve Growth Factor"[Title/Abstract] OR "NGF"[Title/Abstract] OR "nerve growth factor 1"[Title/Abstract] OR "nerve growth factor beta subunit"[Title/Abstract] OR "beta nerve growth factor"[Title/Abstract] OR "beta nerve growth factor"[Title/Abstract] OR ("NGF"[All Fields] AND "1beta"[Title/Abstract]) OR "nerve growth factor alpha subunit"[Title/Abstract] OR "nerve growth factor gamma subunit"[Title/Abstract]) OR "Glial Cell Line-Derived Neurotrophic Factor"[MeSH Terms] OR ("Glial Cell Line-Derived Neurotrophic Factor"[Title/Abstract] OR "GDNF"[Title/Abstract] OR "Glial Cell Line-Derived Neurotrophic Factor"[Title/Abstract] OR "Glial Cell Line-Derived Neurotrophic Factor"[Title/Abstract] OR "glial cell derived neurotrophic factor"[Title/Abstract] OR "glial cell derived neurotrophic factor"[Title/Abstract] OR "glial derived neurotrophic factor"[Title/Abstract] OR "glial derived neurotrophic factor"[Title/Abstract] OR "gdnf growth factor"[Title/Abstract]) OR "Malondialdehyde"[MeSH Terms] OR ("Malondialdehyde"[Title/Abstract] OR "MDA"[Title/Abstract] OR "Malonaldehyde"[Title/Abstract] OR "Malonylaldehyde"[Title/Abstract] OR "Malonyldialdehyde"[Title/Abstract] OR "Propanedial"[Title/Abstract] OR "sodium malondialdehyde"[Title/Abstract]) OR "Superoxide Dismutase"[MeSH Terms] OR ("Superoxide Dismutase"[Title/Abstract] OR "SOD"[Title/Abstract] OR "Erythrocuprein"[Title/Abstract] OR "Hemocuprein"[Title/Abstract] OR "cu superoxide dismutase"[Title/Abstract] OR "cu superoxide dismutase"[Title/Abstract] OR "fe zn superoxide dismutase"[Title/Abstract] OR "fe zn superoxide dismutase"[Title/Abstract] OR "cobalt superoxide dismutase"[Title/Abstract] OR "mn superoxide dismutase"[Title/Abstract] OR "mn superoxide dismutase"[Title/Abstract] OR "Mn-SOD"[Title/Abstract] OR "Mn-SOD"[Title/Abstract] OR "manganese superoxide dismutase"[Title/Abstract] OR "ag zn superoxide dismutase"[Title/Abstract] OR "ag zn superoxide dismutase"[Title/Abstract] OR "iron superoxide dismutase"[Title/Abstract] OR "fe superoxide dismutase"[Title/Abstract] OR "fe superoxide dismutase"[Title/Abstract]) OR "Glutathione"[MeSH Terms] OR ("Glutathione"[Title/Abstract] OR "GSH"[Title/Abstract]) OR "Nitric Oxide"[MeSH Terms] OR ("Nitric Oxide"[Title/Abstract] OR "nitrogen monoxide"[Title/Abstract] OR "mononitrogen monoxide"[Title/Abstract] OR "endogenous nitrate vasodilator"[Title/Abstract] OR "endothelium derived nitric oxide"[Title/Abstract]) OR "Insulin"[MeSH Terms] OR ("Insulin"[Title/Abstract] OR "regular insulin"[Title/Abstract] OR "soluble insulin"[Title/Abstract] OR "Iletin"[Title/Abstract] OR "insulin a chain"[Title/Abstract] OR "Novolin"[Title/Abstract] OR "sodium insulin"[Title/Abstract] OR "insulin b chain"[Title/Abstract]) OR "Glucose"[MeSH Terms] OR ("Glucose"[Title/Abstract] OR "D-Glucose"[Title/Abstract] OR "D-Glucose"[Title/Abstract] OR "Dextrose"[Title/Abstract] OR "glucose monohydrate"[Title/Abstract] OR "anhydrous dextrose"[Title/Abstract]) OR "Triglycerides"[MeSH Terms] OR ("Triglycerides"[Title/Abstract] OR "Triglyceride"[Title/Abstract] OR "Triacylglycerols"[Title/Abstract] OR "Triacylglycerol"[Title/Abstract]) OR "lipoproteins, ldl"[MeSH Terms] OR ("Lipoproteins"[Title/Abstract] OR "lipoproteins ldl"[Title/Abstract] OR "LDL"[Title/Abstract] OR "ldl lipoproteins"[Title/Abstract] OR "beta-Lipoprotein"[Title/Abstract] OR "beta-Lipoprotein"[Title/Abstract] OR "low density lipoprotein"[Title/Abstract] OR "low density lipoprotein"[Title/Abstract] OR "beta-Lipoproteins"[Title/Abstract] OR "beta-Lipoproteins"[Title/Abstract] OR "low density lipoproteins"[Title/Abstract] OR "low density lipoproteins"[Title/Abstract] OR "LDL-1"[Title/Abstract] OR (("oxidized low density lipoprotein"[Supplementary Concept] OR "oxidized low density lipoprotein"[All Fields] OR "LDL"[All Fields]) AND "1"[Title/Abstract]) OR "LDL1"[Title/Abstract] OR "low density lipoprotein 1"[Title/Abstract] OR "low density lipoprotein 1"[Title/Abstract] OR "LDL-2"[Title/Abstract] OR (("oxidized low density lipoprotein"[Supplementary Concept] OR "oxidized low density lipoprotein"[All Fields] OR "LDL"[All Fields]) AND "2"[Title/Abstract]) OR "LDL2"[Title/Abstract] OR "low density lipoprotein 2"[Title/Abstract] OR "low density lipoprotein 2"[Title/Abstract]) OR "Amyloid beta-Peptides"[MeSH Terms] OR ("Amyloid beta-Peptides"[Title/Abstract] OR "Amyloid beta-Peptides"[Title/Abstract] OR "alzheimer beta protein"[Title/Abstract] OR "alzheimer beta protein"[Title/Abstract] OR (("alzheime s"[All Fields] OR "alzheimer disease"[MeSH Terms] OR ("Alzheimer"[All Fields] AND "disease"[All Fields]) OR "alzheimer disease"[All Fields] OR "Alzheimer"[All Fields] OR "alzheimer s"[All Fields] OR "alzheimers"[All Fields] OR "alzheimers s"[All Fields]) AND "ABP"[Title/Abstract]) OR (("alzheime s"[All Fields] OR "alzheimer disease"[MeSH Terms] OR ("Alzheimer"[All Fields] AND "disease"[All Fields]) OR "alzheimer disease"[All Fields] OR "Alzheimer"[All Fields] OR "alzheimer s"[All Fields] OR "alzheimers"[All Fields] OR "alzheimers s"[All Fields]) AND "ABP"[Title/Abstract]) OR (("alzheime s"[All Fields] OR "alzheimer disease"[MeSH Terms] OR ("Alzheimer"[All Fields] AND "disease"[All Fields]) OR "alzheimer disease"[All Fields] OR "Alzheimer"[All Fields] OR "alzheimer s"[All Fields] OR "alzheimers"[All Fields] OR "alzheimers s"[All Fields]) AND "ABP"[Title/Abstract]) OR (("alzheime s"[All Fields] OR "alzheimer disease"[MeSH Terms] OR ("Alzheimer"[All Fields] AND "disease"[All Fields]) OR "alzheimer disease"[All Fields] OR "Alzheimer"[All Fields] OR "alzheimer s"[All Fields] OR "alzheimers"[All Fields] OR "alzheimers s"[All Fields]) AND "amyloid fibril protein"[Title/Abstract]) OR (("Amyloid"[Supplementary Concept] OR "Amyloid"[All Fields] OR "amyloids"[All Fields] OR "Amyloid"[MeSH Terms] OR "amyloidal"[All Fields] OR "amyloidic"[All Fields] OR "app protein human"[Supplementary Concept] OR "app protein human"[All Fields]) AND "AD-AP"[Title/Abstract]) OR (("Amyloid"[Supplementary Concept] OR "Amyloid"[All Fields] OR "amyloids"[All Fields] OR "Amyloid"[MeSH Terms] OR "amyloidal"[All Fields] OR "amyloidic"[All Fields] OR "app protein human"[Supplementary Concept] OR "app protein human"[All Fields]) AND "AD-AP"[Title/Abstract]) OR "amyloid beta peptide"[Title/Abstract] OR "amyloid beta peptide"[Title/Abstract] OR "amyloid beta protein"[Title/Abstract] OR "amyloid beta protein"[Title/Abstract] OR "amyloid beta proteins"[Title/Abstract] OR "amyloid beta proteins"[Title/Abstract] OR "amyloid protein a4"[Title/Abstract] OR "beta-Amyloid"[Title/Abstract] OR "beta amyloid protein"[Title/Abstract] OR "beta amyloid protein"[Title/Abstract]) OR "tau Proteins"[MeSH Terms] OR ("tau Proteins"[Title/Abstract] OR "tau protein"[Title/Abstract]) OR "fatty acids, volatile"[MeSH Terms] OR ("fatty acids volatile"[Title/Abstract] OR "volatile fatty acids"[Title/Abstract] OR "short chain fatty acids"[Title/Abstract] OR "short chain fatty acid"[Title/Abstract] OR "short chain fatty acid"[Title/Abstract] OR "volatile fatty acid"[Title/Abstract]) OR "Tryptophan"[MeSH Terms] OR ("Tryptophan"[Title/Abstract] OR "Levotryptophan"[Title/Abstract] OR "L-Tryptophan"[Title/Abstract] OR "L-Tryptophan"[Title/Abstract] OR "tryptophan metabolism alterations"[Title/Abstract] OR "Tryptan"[Title/Abstract] OR "Ardeytropin"[Title/Abstract] OR (("Tryptophan"[Supplementary Concept] OR "Tryptophan"[All Fields] OR "L-Tryptophan"[All Fields] OR "Tryptophan"[MeSH Terms]) AND "ratiopharm"[Title/Abstract]) OR "ratio-Tryptophan"[Title/Abstract] OR "ratio-Tryptophan"[Title/Abstract] OR "Lyphan"[Title/Abstract] OR ("PMS"[All Fields] AND "Tryptophan"[Title/Abstract]) OR "Optimax"[Title/Abstract]) OR "Kynurenine"[MeSH Terms] OR "Kynurenine"[Title/Abstract] OR ("neurotransmit*"[Title/Abstract] OR "hormone*"[Title/Abstract] OR "cytokine*"[Title/Abstract] OR "neurotrophic*"[Title/Abstract] OR "oxidative stress*"[Title/Abstract] OR "metabolic*"[Title/Abstract]) OR ("biomarker*"[Title/Abstract] OR "marker*"[Title/Abstract] OR "parameter*"[Title/Abstract] OR "index*"[Title/Abstract]) | 8,287,566 |
| 91 | (((biomarker*[Title/Abstract]) OR (marker*[Title/Abstract])) OR (parameter*[Title/Abstract])) OR (index*[Title/Abstract]) | "biomarker*"[Title/Abstract] OR "marker*"[Title/Abstract] OR "parameter*"[Title/Abstract] OR "index*"[Title/Abstract] | 4,045,507 |
| 90 | (((((neurotransmit*[Title/Abstract]) OR (hormone*[Title/Abstract])) OR (cytokine*[Title/Abstract])) OR (neurotrophic*[Title/Abstract])) OR (oxidative stress*[Title/Abstract])) OR (metabolic*[Title/Abstract]) | "neurotransmit*"[Title/Abstract] OR "hormone*"[Title/Abstract] OR "cytokine*"[Title/Abstract] OR "neurotrophic*"[Title/Abstract] OR "oxidative stress*"[Title/Abstract] OR "metabolic*"[Title/Abstract] | 2,265,252 |
| 89 | Kynurenine[Title/Abstract] | "Kynurenine"[Title/Abstract] | 8,251 |
| 88 | "Kynurenine"[Mesh] | "Kynurenine"[MeSH Terms] | 4,812 |
| 87 | ((((((((((((((((((Tryptophan[Title/Abstract]) OR (Levotryptophan[Title/Abstract])) OR (L-Tryptophan[Title/Abstract])) OR (L Tryptophan[Title/Abstract])) OR (Tryptophan Metabolism Alterations[Title/Abstract])) OR (Tryptan[Title/Abstract])) OR (Tryptacin[Title/Abstract])) OR (Trofan[Title/Abstract])) OR (Ardeydorm[Title/Abstract])) OR (Ardeytropin[Title/Abstract])) OR (L-Tryptophan-ratiopharm[Title/Abstract])) OR (L Tryptophan ratiopharm[Title/Abstract])) OR (ratio-Tryptophan[Title/Abstract])) OR (ratio Tryptophan[Title/Abstract])) OR (Lyphan[Title/Abstract])) OR (Naturruhe[Title/Abstract])) OR (PMS-Tryptophan[Title/Abstract])) OR (PMS Tryptophan[Title/Abstract])) OR (Optimax[Title/Abstract]) | "Tryptophan"[Title/Abstract] OR "Levotryptophan"[Title/Abstract] OR "L-Tryptophan"[Title/Abstract] OR "L-Tryptophan"[Title/Abstract] OR "tryptophan metabolism alterations"[Title/Abstract] OR "Tryptan"[Title/Abstract] OR "Ardeytropin"[Title/Abstract] OR (("Tryptophan"[Supplementary Concept] OR "Tryptophan"[All Fields] OR "L-Tryptophan"[All Fields] OR "Tryptophan"[MeSH Terms]) AND "ratiopharm"[Title/Abstract]) OR "ratio-Tryptophan"[Title/Abstract] OR "ratio-Tryptophan"[Title/Abstract] OR "Lyphan"[Title/Abstract] OR ("PMS"[All Fields] AND "Tryptophan"[Title/Abstract]) OR "Optimax"[Title/Abstract] | 63,992 |
| 86 | "Tryptophan"[Mesh] | "Tryptophan"[MeSH Terms] | 38,152 |
| 85 | (((((Fatty Acids, Volatile[Title/Abstract]) OR (Volatile Fatty Acids[Title/Abstract])) OR (Short-Chain Fatty Acids[Title/Abstract])) OR (Short-Chain Fatty Acid[Title/Abstract])) OR (Short Chain Fatty Acid[Title/Abstract])) OR (Volatile Fatty Acid[Title/Abstract]) | "fatty acids volatile"[Title/Abstract] OR "volatile fatty acids"[Title/Abstract] OR "short chain fatty acids"[Title/Abstract] OR "short chain fatty acid"[Title/Abstract] OR "short chain fatty acid"[Title/Abstract] OR "volatile fatty acid"[Title/Abstract] | 31,308 |
| 84 | "Fatty Acids, Volatile"[Mesh] | "fatty acids, volatile"[MeSH Terms] | 123,983 |
| 83 | (tau Proteins[Title/Abstract]) OR (tau Protein[Title/Abstract]) | "tau proteins"[Title/Abstract] OR "tau protein"[Title/Abstract] | 11,131 |
| 82 | "tau Proteins"[Mesh] | "tau Proteins"[MeSH Terms] | 23,514 |
| 81 | (((((((((((((((((((Amyloid beta-Peptides[Title/Abstract]) OR (Amyloid beta Peptides[Title/Abstract])) OR (Alzheimer beta-Protein[Title/Abstract])) OR (Alzheimer beta Protein[Title/Abstract])) OR (Alzheimer's ABP[Title/Abstract])) OR (Alzheimer ABP[Title/Abstract])) OR (Alzheimers ABP[Title/Abstract])) OR (Alzheimer's Amyloid Fibril Protein[Title/Abstract])) OR (Amyloid AD-AP[Title/Abstract])) OR (Amyloid AD AP[Title/Abstract])) OR (Amyloid beta-Peptide[Title/Abstract])) OR (Amyloid beta Peptide[Title/Abstract])) OR (Amyloid beta-Protein[Title/Abstract])) OR (Amyloid beta Protein[Title/Abstract])) OR (Amyloid beta-Proteins[Title/Abstract])) OR (Amyloid beta Proteins[Title/Abstract])) OR (Amyloid Protein A4[Title/Abstract])) OR (beta Amyloid[Title/Abstract])) OR (beta-Amyloid Protein[Title/Abstract])) OR (beta Amyloid Protein[Title/Abstract]) | "amyloid beta peptides"[Title/Abstract] OR "amyloid beta peptides"[Title/Abstract] OR "alzheimer beta protein"[Title/Abstract] OR "alzheimer beta protein"[Title/Abstract] OR (("alzheime s"[All Fields] OR "alzheimer disease"[MeSH Terms] OR ("Alzheimer"[All Fields] AND "disease"[All Fields]) OR "alzheimer disease"[All Fields] OR "Alzheimer"[All Fields] OR "alzheimer s"[All Fields] OR "alzheimers"[All Fields] OR "alzheimers s"[All Fields]) AND "ABP"[Title/Abstract]) OR (("alzheime s"[All Fields] OR "alzheimer disease"[MeSH Terms] OR ("Alzheimer"[All Fields] AND "disease"[All Fields]) OR "alzheimer disease"[All Fields] OR "Alzheimer"[All Fields] OR "alzheimer s"[All Fields] OR "alzheimers"[All Fields] OR "alzheimers s"[All Fields]) AND "ABP"[Title/Abstract]) OR (("alzheime s"[All Fields] OR "alzheimer disease"[MeSH Terms] OR ("Alzheimer"[All Fields] AND "disease"[All Fields]) OR "alzheimer disease"[All Fields] OR "Alzheimer"[All Fields] OR "alzheimer s"[All Fields] OR "alzheimers"[All Fields] OR "alzheimers s"[All Fields]) AND "ABP"[Title/Abstract]) OR (("alzheime s"[All Fields] OR "alzheimer disease"[MeSH Terms] OR ("Alzheimer"[All Fields] AND "disease"[All Fields]) OR "alzheimer disease"[All Fields] OR "Alzheimer"[All Fields] OR "alzheimer s"[All Fields] OR "alzheimers"[All Fields] OR "alzheimers s"[All Fields]) AND "amyloid fibril protein"[Title/Abstract]) OR (("Amyloid"[Supplementary Concept] OR "Amyloid"[All Fields] OR "amyloids"[All Fields] OR "Amyloid"[MeSH Terms] OR "amyloidal"[All Fields] OR "amyloidic"[All Fields] OR "app protein human"[Supplementary Concept] OR "app protein human"[All Fields]) AND "AD-AP"[Title/Abstract]) OR (("Amyloid"[Supplementary Concept] OR "Amyloid"[All Fields] OR "amyloids"[All Fields] OR "Amyloid"[MeSH Terms] OR "amyloidal"[All Fields] OR "amyloidic"[All Fields] OR "app protein human"[Supplementary Concept] OR "app protein human"[All Fields]) AND "AD-AP"[Title/Abstract]) OR "amyloid beta peptide"[Title/Abstract] OR "amyloid beta peptide"[Title/Abstract] OR "amyloid beta protein"[Title/Abstract] OR "amyloid beta protein"[Title/Abstract] OR "amyloid beta proteins"[Title/Abstract] OR "amyloid beta proteins"[Title/Abstract] OR "amyloid protein a4"[Title/Abstract] OR "beta-Amyloid"[Title/Abstract] OR "beta amyloid protein"[Title/Abstract] OR "beta amyloid protein"[Title/Abstract] | 33,323 |
| 80 | "Amyloid beta-Peptides"[Mesh] | "Amyloid beta-Peptides"[MeSH Terms] | 47,499 |
| 79 | (((((((((((((((((((((Lipoproteins[Title/Abstract]) OR (Lipoproteins, LDL[Title/Abstract])) OR (LDL[Title/Abstract])) OR (LDL Lipoproteins[Title/Abstract])) OR (beta-Lipoprotein[Title/Abstract])) OR (beta Lipoprotein[Title/Abstract])) OR (Low-Density Lipoprotein[Title/Abstract])) OR (Low Density Lipoprotein[Title/Abstract])) OR (beta-Lipoproteins[Title/Abstract])) OR (beta Lipoproteins[Title/Abstract])) OR (Low-Density Lipoproteins[Title/Abstract])) OR (Low Density Lipoproteins[Title/Abstract])) OR (LDL-1[Title/Abstract])) OR (LDL(1[Title/Abstract]))) OR (LDL1[Title/Abstract])) OR (Low-Density Lipoprotein 1[Title/Abstract])) OR (Low Density Lipoprotein 1[Title/Abstract])) OR (LDL-2[Title/Abstract])) OR (LDL(2[Title/Abstract]))) OR (LDL2[Title/Abstract])) OR (Low-Density Lipoprotein 2[Title/Abstract])) OR (Low Density Lipoprotein 2[Title/Abstract]) | "Lipoproteins"[Title/Abstract] OR "lipoproteins ldl"[Title/Abstract] OR "LDL"[Title/Abstract] OR "ldl lipoproteins"[Title/Abstract] OR "beta-Lipoprotein"[Title/Abstract] OR "beta-Lipoprotein"[Title/Abstract] OR "low density lipoprotein"[Title/Abstract] OR "low density lipoprotein"[Title/Abstract] OR "beta-Lipoproteins"[Title/Abstract] OR "beta-Lipoproteins"[Title/Abstract] OR "low density lipoproteins"[Title/Abstract] OR "low density lipoproteins"[Title/Abstract] OR "LDL-1"[Title/Abstract] OR (("oxidized low density lipoprotein"[Supplementary Concept] OR "oxidized low density lipoprotein"[All Fields] OR "LDL"[All Fields]) AND "1"[Title/Abstract]) OR "LDL1"[Title/Abstract] OR "low density lipoprotein 1"[Title/Abstract] OR "low density lipoprotein 1"[Title/Abstract] OR "LDL-2"[Title/Abstract] OR (("oxidized low density lipoprotein"[Supplementary Concept] OR "oxidized low density lipoprotein"[All Fields] OR "LDL"[All Fields]) AND "2"[Title/Abstract]) OR "LDL2"[Title/Abstract] OR "low density lipoprotein 2"[Title/Abstract] OR "low density lipoprotein 2"[Title/Abstract] | 176,073 |
| 78 | "Lipoproteins, LDL"[Mesh] | "lipoproteins, ldl"[MeSH Terms] | 63,716 |
| 77 | (((Triglycerides[Title/Abstract]) OR (Triglyceride[Title/Abstract])) OR (Triacylglycerols[Title/Abstract])) OR (Triacylglycerol[Title/Abstract]) | "Triglycerides"[Title/Abstract] OR "Triglyceride"[Title/Abstract] OR "Triacylglycerols"[Title/Abstract] OR "Triacylglycerol"[Title/Abstract] | 174,334 |
| 76 | "Triglycerides"[Mesh] | "Triglycerides"[MeSH Terms] | 90,696 |
| 75 | (((((Glucose[Title/Abstract]) OR (D-Glucose[Title/Abstract])) OR (D Glucose[Title/Abstract])) OR (Dextrose[Title/Abstract])) OR (Glucose Monohydrate[Title/Abstract])) OR (Anhydrous Dextrose[Title/Abstract]) | "Glucose"[Title/Abstract] OR "D-Glucose"[Title/Abstract] OR "D-Glucose"[Title/Abstract] OR "Dextrose"[Title/Abstract] OR "glucose monohydrate"[Title/Abstract] OR "anhydrous dextrose"[Title/Abstract] | 650,648 |
| 74 | "Glucose"[Mesh] | "Glucose"[MeSH Terms] | 363,441 |
| 73 | (((((((Insulin[Title/Abstract]) OR (Regular Insulin[Title/Abstract])) OR (Soluble Insulin[Title/Abstract])) OR (Iletin[Title/Abstract])) OR (Insulin A Chain[Title/Abstract])) OR (Novolin[Title/Abstract])) OR (Sodium Insulin[Title/Abstract])) OR (Insulin B Chain[Title/Abstract]) | "Insulin"[Title/Abstract] OR "regular insulin"[Title/Abstract] OR "soluble insulin"[Title/Abstract] OR "Iletin"[Title/Abstract] OR "insulin a chain"[Title/Abstract] OR "Novolin"[Title/Abstract] OR "sodium insulin"[Title/Abstract] OR "insulin b chain"[Title/Abstract] | 451,767 |
| 72 | "Insulin"[Mesh] | "Insulin"[MeSH Terms] | 207,582 |
| 71 | (((((Nitric oxide[Title/Abstract]) OR (NO[Title/Abstract])) OR (Nitrogen Monoxide[Title/Abstract])) OR (Mononitrogen Monoxide[Title/Abstract])) OR (Endogenous Nitrate Vasodilator[Title/Abstract])) OR (Endothelium-Derived Nitric Oxide[Title/Abstract]) | "nitric oxide"[Title/Abstract] OR "nitrogen monoxide"[Title/Abstract] OR "mononitrogen monoxide"[Title/Abstract] OR "endogenous nitrate vasodilator"[Title/Abstract] OR "endothelium derived nitric oxide"[Title/Abstract] | 180,395 |
| 70 | "Nitric Oxide"[Mesh] | "Nitric Oxide"[MeSH Terms] | 101,397 |
| 69 | (Glutathione[Title/Abstract]) OR (GSH[Title/Abstract]) | "Glutathione"[Title/Abstract] OR "GSH"[Title/Abstract] | 191,159 |
| 68 | "Glutathione"[Mesh] | "Glutathione"[MeSH Terms] | 67,428 |
| 67 | ((((((((((((((((((Superoxide dismutase[Title/Abstract]) OR (SOD[Title/Abstract])) OR (Erythrocuprein[Title/Abstract])) OR (Hemocuprein[Title/Abstract])) OR (Cu-Superoxide Dismutase[Title/Abstract])) OR (Cu Superoxide Dismutase[Title/Abstract])) OR (Fe-Zn Superoxide Dismutase[Title/Abstract])) OR (Fe Zn Superoxide Dismutase[Title/Abstract])) OR (Cobalt Superoxide Dismutase[Title/Abstract])) OR (Mn-Superoxide Dismutase[Title/Abstract])) OR (Mn Superoxide Dismutase[Title/Abstract])) OR (Mn-SOD[Title/Abstract])) OR (Mn SOD[Title/Abstract])) OR (Manganese Superoxide Dismutase[Title/Abstract])) OR (Ag-Zn Superoxide Dismutase[Title/Abstract])) OR (Ag Zn Superoxide Dismutase[Title/Abstract])) OR (Iron Superoxide Dismutase[Title/Abstract])) OR (Fe-Superoxide Dismutase[Title/Abstract])) OR (Fe Superoxide Dismutase[Title/Abstract]) | "superoxide dismutase"[Title/Abstract] OR "SOD"[Title/Abstract] OR "Erythrocuprein"[Title/Abstract] OR "Hemocuprein"[Title/Abstract] OR "cu superoxide dismutase"[Title/Abstract] OR "cu superoxide dismutase"[Title/Abstract] OR "fe zn superoxide dismutase"[Title/Abstract] OR "fe zn superoxide dismutase"[Title/Abstract] OR "cobalt superoxide dismutase"[Title/Abstract] OR "mn superoxide dismutase"[Title/Abstract] OR "mn superoxide dismutase"[Title/Abstract] OR "Mn-SOD"[Title/Abstract] OR "Mn-SOD"[Title/Abstract] OR "manganese superoxide dismutase"[Title/Abstract] OR "ag zn superoxide dismutase"[Title/Abstract] OR "ag zn superoxide dismutase"[Title/Abstract] OR "iron superoxide dismutase"[Title/Abstract] OR "fe superoxide dismutase"[Title/Abstract] OR "fe superoxide dismutase"[Title/Abstract] | 125,790 |
| 66 | "Superoxide Dismutase"[Mesh] | "Superoxide Dismutase"[MeSH Terms] | 61,352 |
| 65 | ((((((Malondialdehyde[Title/Abstract]) OR (MDA[Title/Abstract])) OR (Malonaldehyde[Title/Abstract])) OR (Malonylaldehyde[Title/Abstract])) OR (Malonyldialdehyde[Title/Abstract])) OR (Propanedial[Title/Abstract])) OR (Sodium Malondialdehyde[Title/Abstract]) | "Malondialdehyde"[Title/Abstract] OR "MDA"[Title/Abstract] OR "Malonaldehyde"[Title/Abstract] OR "Malonylaldehyde"[Title/Abstract] OR "Malonyldialdehyde"[Title/Abstract] OR "Propanedial"[Title/Abstract] OR "sodium malondialdehyde"[Title/Abstract] | 130,004 |
| 64 | "Malondialdehyde"[Mesh] | "Malondialdehyde"[MeSH Terms] | 39,809 |
| 63 | ((((((((Glial cell line‐derived neurotrophic factor[Title/Abstract]) OR (GDNF[Title/Abstract])) OR (Glial Cell Line Derived Neurotrophic Factor[Title/Abstract])) OR (Glial Cell-Line Derived Neurotrophic Factor[Title/Abstract])) OR (Glial Cell-Derived Neurotrophic Factor[Title/Abstract])) OR (Glial Cell Derived Neurotrophic Factor[Title/Abstract])) OR (Glial-Derived Neurotrophic Factor[Title/Abstract])) OR (Glial Derived Neurotrophic Factor[Title/Abstract])) OR (GDNF Growth Factor[Title/Abstract]) | "glial cell line derived neurotrophic factor"[Title/Abstract] OR "GDNF"[Title/Abstract] OR "glial cell line derived neurotrophic factor"[Title/Abstract] OR "glial cell line derived neurotrophic factor"[Title/Abstract] OR "glial cell derived neurotrophic factor"[Title/Abstract] OR "glial cell derived neurotrophic factor"[Title/Abstract] OR "glial derived neurotrophic factor"[Title/Abstract] OR "glial derived neurotrophic factor"[Title/Abstract] OR "gdnf growth factor"[Title/Abstract] | 6,814 |
| 62 | "Glial Cell Line-Derived Neurotrophic Factor"[Mesh] | "Glial Cell Line-Derived Neurotrophic Factor"[MeSH Terms] | 3,969 |
| 61 | (((((((((Nerve growth factor[Title/Abstract]) OR (NGF[Title/Abstract])) OR (Nerve Growth Factor 1[Title/Abstract])) OR (Nerve Growth Factor beta Subunit[Title/Abstract])) OR (beta-Nerve Growth Factor[Title/Abstract])) OR (beta Nerve Growth Factor[Title/Abstract])) OR (NGF-1beta[Title/Abstract])) OR (NGF 1beta[Title/Abstract])) OR (Nerve Growth Factor alpha Subunit[Title/Abstract])) OR (Nerve Growth Factor gamma Subunit[Title/Abstract]) | "nerve growth factor"[Title/Abstract] OR "NGF"[Title/Abstract] OR "nerve growth factor 1"[Title/Abstract] OR "nerve growth factor beta subunit"[Title/Abstract] OR "beta nerve growth factor"[Title/Abstract] OR "beta nerve growth factor"[Title/Abstract] OR ("NGF"[All Fields] AND "1beta"[Title/Abstract]) OR "nerve growth factor alpha subunit"[Title/Abstract] OR "nerve growth factor gamma subunit"[Title/Abstract] | 25,607 |
| 60 | "Nerve Growth Factor"[Mesh] | "Nerve Growth Factor"[MeSH Terms] | 8,354 |
| 59 | ((Brain‐derived neurotrophic factor[Title/Abstract]) OR (BDNF[Title/Abstract])) OR (Brain Derived Neurotrophic Factor[Title/Abstract]) | "brain derived neurotrophic factor"[Title/Abstract] OR "BDNF"[Title/Abstract] OR "brain derived neurotrophic factor"[Title/Abstract] | 36,313 |
| 58 | "Brain-Derived Neurotrophic Factor"[Mesh] | "Brain-Derived Neurotrophic Factor"[MeSH Terms] | 22,304 |
| 57 | (((((((((((((((Serum Amyloid A Protein[Title/Abstract]) OR (Amyloid A Precursor[Title/Abstract])) OR (Amyloid A Protein-Related Serum Component[Title/Abstract])) OR (Amyloid A Protein Related Serum Component[Title/Abstract])) OR (Amyloid Protein AA Precursor[Title/Abstract])) OR (Amyloid Protein SAA[Title/Abstract])) OR (Amyloid Serum Protein SAA[Title/Abstract])) OR (Amyloid-Related Serum Protein (SAA[Title/Abstract]))) OR (Serum A Related Protein[Title/Abstract])) OR (Serum Amyloid A[Title/Abstract])) OR (Serum Amyloid Protein A[Title/Abstract])) OR (Amyloid A Protein[Title/Abstract])) OR (Amyloid Fibril Protein AA[Title/Abstract])) OR (Amyloid Protein AA[Title/Abstract])) OR (Amyloid-Related Serum Protein[Title/Abstract])) OR (SAA[Title/Abstract]) | "serum amyloid a protein"[Title/Abstract] OR "amyloid a precursor"[Title/Abstract] OR "amyloid a protein related serum component"[Title/Abstract] OR "amyloid a protein related serum component"[Title/Abstract] OR (("amyloidogenic proteins"[Supplementary Concept] OR "amyloidogenic proteins"[All Fields] OR "amyloid protein"[All Fields] OR "amyloidogenic proteins"[MeSH Terms] OR ("amyloidogenic"[All Fields] AND "proteins"[All Fields]) OR ("Amyloid"[All Fields] AND "Protein"[All Fields])) AND "aa precursor"[Title/Abstract]) OR "amyloid protein saa"[Title/Abstract] OR "amyloid serum protein saa"[Title/Abstract] OR (("Amyloid-Related"[All Fields] AND ("blood proteins"[Supplementary Concept] OR "blood proteins"[All Fields] OR "serum protein"[All Fields] OR "blood proteins"[MeSH Terms] OR ("blood"[All Fields] AND "proteins"[All Fields]) OR ("Serum"[All Fields] AND "Protein"[All Fields]))) AND "SAA"[Title/Abstract]) OR (("Serum"[MeSH Terms] OR "Serum"[All Fields] OR "serums"[All Fields] OR "serum s"[All Fields] OR "serumal"[All Fields]) AND "a related protein"[Title/Abstract]) OR "serum amyloid a"[Title/Abstract] OR "serum amyloid protein a"[Title/Abstract] OR "amyloid a protein"[Title/Abstract] OR "amyloid fibril protein aa"[Title/Abstract] OR "amyloid protein aa"[Title/Abstract] OR "amyloid related serum protein"[Title/Abstract] OR "SAA"[Title/Abstract] | 11,110 |
| 56 | "Serum Amyloid A Protein"[Mesh] | "Serum Amyloid A Protein"[MeSH Terms] | 4,792 |
| 55 | (((((C‐reactive protein[Title/Abstract]) OR (C Reactive Protein[Title/Abstract])) OR (High Sensitivity C-Reactive Protein[Title/Abstract])) OR (High Sensitivity C Reactive Protein[Title/Abstract])) OR (hs-CRP[Title/Abstract])) OR (hsCRP[Title/Abstract]) | "c reactive protein"[Title/Abstract] OR "c reactive protein"[Title/Abstract] OR "high sensitivity c reactive protein"[Title/Abstract] OR "high sensitivity c reactive protein"[Title/Abstract] OR "hs-CRP"[Title/Abstract] OR "hsCRP"[Title/Abstract] | 113,018 |
| 54 | "C-Reactive Protein"[Mesh] | "C-Reactive Protein"[MeSH Terms] | 60,692 |
| 53 | ((((((Transforming Growth Factor beta[Title/Abstract]) OR (Platelet Transforming Growth Factor[Title/Abstract])) OR (Bone-Derived Transforming Growth Factor[Title/Abstract])) OR (Bone Derived Transforming Growth Factor[Title/Abstract])) OR (TGF-beta[Title/Abstract])) OR (TGFbeta[Title/Abstract])) OR (Milk Growth Factor[Title/Abstract]) | "transforming growth factor beta"[Title/Abstract] OR "platelet transforming growth factor"[Title/Abstract] OR "bone derived transforming growth factor"[Title/Abstract] OR "bone derived transforming growth factor"[Title/Abstract] OR "TGF-beta"[Title/Abstract] OR "TGFbeta"[Title/Abstract] OR "milk growth factor"[Title/Abstract] | 100,786 |
| 52 | "Transforming Growth Factor beta"[Mesh] | "Transforming Growth Factor beta"[MeSH Terms] | 75,138 |
| 51 | ((((Interferon-gamma[Title/Abstract]) OR (Interferon Type II[Title/Abstract])) OR (Immune Interferon[Title/Abstract])) OR (Type II Interferon[Title/Abstract])) OR (gamma-Interferon[Title/Abstract]) | "Interferon-gamma"[Title/Abstract] OR "interferon type ii"[Title/Abstract] OR "immune interferon"[Title/Abstract] OR "type ii interferon"[Title/Abstract] OR "gamma-Interferon"[Title/Abstract] | 72,218 |
| 50 | "Interferon-gamma"[Mesh] | "Interferon-gamma"[MeSH Terms] | 74,208 |
| 49 | (((((Interleukin‐10[Title/Abstract]) OR (Interleukin 10[Title/Abstract])) OR (CSIF-10[Title/Abstract])) OR (Cytokine Synthesis Inhibitory Factor[Title/Abstract])) OR (IL-10[Title/Abstract])) OR (IL10[Title/Abstract]) | "Interleukin-10"[Title/Abstract] OR "Interleukin-10"[Title/Abstract] OR "cytokine synthesis inhibitory factor"[Title/Abstract] OR "IL-10"[Title/Abstract] OR "IL10"[Title/Abstract] | 93,224 |
| 48 | "Interleukin-10"[Mesh] | "Interleukin-10"[MeSH Terms] | 33,992 |
| 47 | (((((((((((((((((((Interleukin‐8[Title/Abstract]) OR (Interleukin 8[Title/Abstract])) OR (IL-8[Title/Abstract])) OR (IL8[Title/Abstract])) OR (Monocyte-Derived Neutrophil Chemotactic Factor[Title/Abstract])) OR (Neutrophil Activation Factor[Title/Abstract])) OR (Lymphocyte-Derived Neutrophil-Activating Peptide[Title/Abstract])) OR (Monocyte-Derived Neutrophil-Activating Peptide[Title/Abstract])) OR (Alveolar Macrophage Chemotactic Factor-I[Title/Abstract])) OR (Alveolar Macrophage Chemotactic Factor I[Title/Abstract])) OR (Anionic Neutrophil-Activating Peptide[Title/Abstract])) OR (Anionic Neutrophil Activating Peptide[Title/Abstract])) OR (Chemokine CXCL8[Title/Abstract])) OR (CXCL8 Chemokines[Title/Abstract])) OR (Macrophage-Derived Chemotactic Factor[Title/Abstract])) OR (Neutrophil Chemotactic Factor[Title/Abstract])) OR (CXCL8 Chemokine[Title/Abstract])) OR (Granulocyte Chemotactic Peptide-Interleukin-8[Title/Abstract])) OR (Granulocyte Chemotactic Peptide Interleukin 8[Title/Abstract])) OR (AMCF-I[Title/Abstract]) | "Interleukin-8"[Title/Abstract] OR "Interleukin-8"[Title/Abstract] OR "IL-8"[Title/Abstract] OR "IL8"[Title/Abstract] OR "monocyte derived neutrophil chemotactic factor"[Title/Abstract] OR "neutrophil activation factor"[Title/Abstract] OR "lymphocyte derived neutrophil activating peptide"[Title/Abstract] OR "monocyte derived neutrophil activating peptide"[Title/Abstract] OR ((("macrophages, alveolar"[MeSH Terms] OR ("macrophages"[All Fields] AND "alveolar"[All Fields]) OR "alveolar macrophages"[All Fields] OR ("alveolar"[All Fields] AND "macrophage"[All Fields]) OR "alveolar macrophage"[All Fields]) AND ("chemotactant"[All Fields] OR "Chemotactic"[All Fields] OR "chemotactically"[All Fields] OR "chemotactism"[All Fields])) AND "Factor-I"[Title/Abstract]) OR ((("macrophages, alveolar"[MeSH Terms] OR ("macrophages"[All Fields] AND "alveolar"[All Fields]) OR "alveolar macrophages"[All Fields] OR ("alveolar"[All Fields] AND "macrophage"[All Fields]) OR "alveolar macrophage"[All Fields]) AND ("chemotactant"[All Fields] OR "Chemotactic"[All Fields] OR "chemotactically"[All Fields] OR "chemotactism"[All Fields])) AND "Factor-I"[Title/Abstract]) OR "anionic neutrophil activating peptide"[Title/Abstract] OR "anionic neutrophil activating peptide"[Title/Abstract] OR "chemokine cxcl8"[Title/Abstract] OR "cxcl8 chemokines"[Title/Abstract] OR "macrophage derived chemotactic factor"[Title/Abstract] OR "neutrophil chemotactic factor"[Title/Abstract] OR "cxcl8 chemokine"[Title/Abstract] OR "granulocyte chemotactic peptide interleukin 8"[Title/Abstract] OR "granulocyte chemotactic peptide interleukin 8"[Title/Abstract] OR "AMCF-I"[Title/Abstract] | 58,468 |
| 46 | "Interleukin-8"[Mesh] | "Interleukin-8"[MeSH Terms] | 25,041 |
| 45 | (((((((((((((((((((((((((((Interleukin‐17[Title/Abstract]) OR (CTLA-8[Title/Abstract])) OR (IL-17[Title/Abstract])) OR (Interleukin 17[Title/Abstract])) OR (Interleukin-17B[Title/Abstract])) OR (Interleukin 17B[Title/Abstract])) OR (IL-17B[Title/Abstract])) OR (Interleukin-17A[Title/Abstract])) OR (Interleukin 17A[Title/Abstract])) OR (CTLA8[Title/Abstract])) OR (Cytotoxic T lymphocyte-Associated Antigen 8[Title/Abstract])) OR (Cytotoxic T lymphocyte Associated Antigen 8[Title/Abstract])) OR (IL-17A[Title/Abstract])) OR (Interleukin-17C[Title/Abstract])) OR (Interleukin 17C[Title/Abstract])) OR (Cytokine CX2[Title/Abstract])) OR (IL-17C[Title/Abstract])) OR (Interleukin-17E[Title/Abstract])) OR (Interleukin 17E[Title/Abstract])) OR (IL-17E[Title/Abstract])) OR (IL 17E[Title/Abstract])) OR (Interleukin-25[Title/Abstract])) OR (Interleukin 25[Title/Abstract])) OR (Interleukin-17F[Title/Abstract])) OR (Interleukin 17F[Title/Abstract])) OR (Cytokine ML-1[Title/Abstract])) OR (Cytokine ML 1[Title/Abstract])) OR (IL-17F[Title/Abstract]) | "Interleukin-17"[Title/Abstract] OR "CTLA-8"[Title/Abstract] OR "IL-17"[Title/Abstract] OR "Interleukin-17"[Title/Abstract] OR "Interleukin-17B"[Title/Abstract] OR "Interleukin-17B"[Title/Abstract] OR "IL-17B"[Title/Abstract] OR "Interleukin-17A"[Title/Abstract] OR "Interleukin-17A"[Title/Abstract] OR "CTLA8"[Title/Abstract] OR "cytotoxic t lymphocyte associated antigen 8"[Title/Abstract] OR "cytotoxic t lymphocyte associated antigen 8"[Title/Abstract] OR "IL-17A"[Title/Abstract] OR "Interleukin-17C"[Title/Abstract] OR "Interleukin-17C"[Title/Abstract] OR (("cytokin"[All Fields] OR "cytokine s"[All Fields] OR "cytokines"[Supplementary Concept] OR "cytokines"[All Fields] OR "Cytokine"[All Fields] OR "cytokines"[MeSH Terms] OR "cytokinic"[All Fields] OR "cytokins"[All Fields]) AND "CX2"[Title/Abstract]) OR "IL-17C"[Title/Abstract] OR "Interleukin-17E"[Title/Abstract] OR "Interleukin-17E"[Title/Abstract] OR "IL-17E"[Title/Abstract] OR "IL-17E"[Title/Abstract] OR "Interleukin-25"[Title/Abstract] OR "Interleukin-25"[Title/Abstract] OR "Interleukin-17F"[Title/Abstract] OR "Interleukin-17F"[Title/Abstract] OR "cytokine ml 1"[Title/Abstract] OR "cytokine ml 1"[Title/Abstract] OR "IL-17F"[Title/Abstract] | 41,246 |
| 44 | "Interleukin-17"[Mesh] | "Interleukin-17"[MeSH Terms] | 16,863 |
| 43 | ((((((((Tumor Necrosis Factor-alpha[Title/Abstract]) OR (Tumor Necrosis Factor alpha[Title/Abstract])) OR (TNF-alpha[Title/Abstract])) OR (Tumor Necrosis Factor[Title/Abstract])) OR (Tumor Necrosis Factor Ligand Superfamily Member 2[Title/Abstract])) OR (TNFalpha[Title/Abstract])) OR (Cachectin-Tumor Necrosis Factor[Title/Abstract])) OR (Cachectin Tumor Necrosis Factor[Title/Abstract])) OR (Cachectin[Title/Abstract]) | "tumor necrosis factor alpha"[Title/Abstract] OR "tumor necrosis factor alpha"[Title/Abstract] OR "TNF-alpha"[Title/Abstract] OR "tumor necrosis factor"[Title/Abstract] OR ((("tumour necrosis factor"[All Fields] OR "tumor necrosis factor alpha"[Supplementary Concept] OR "tumor necrosis factor alpha"[All Fields] OR "tumor necrosis factor"[All Fields] OR "tumor necrosis factor alpha"[MeSH Terms] OR ("Tumor"[All Fields] AND "Necrosis"[All Fields] AND "Factor-alpha"[All Fields]) OR ("Tumor"[All Fields] AND "Necrosis"[All Fields] AND "Factor"[All Fields])) AND ("ligand s"[All Fields] OR "liganded"[All Fields] OR "liganding"[All Fields] OR "ligands"[Supplementary Concept] OR "ligands"[All Fields] OR "ligand"[All Fields] OR "ligands"[MeSH Terms])) AND "superfamily member 2"[Title/Abstract]) OR "TNFalpha"[Title/Abstract] OR "cachectin tumor necrosis factor"[Title/Abstract] OR "cachectin tumor necrosis factor"[Title/Abstract] OR "Cachectin"[Title/Abstract] | 303,585 |
| 42 | "Tumor Necrosis Factor-alpha"[Mesh] | "Tumor Necrosis Factor-alpha"[MeSH Terms] | 147,398 |
| 41 | (((((Interleukin‐1beta[Title/Abstract]) OR (Interleukin 1beta[Title/Abstract])) OR (Catabolin[Title/Abstract])) OR (IL-1 beta[Title/Abstract])) OR (Interleukin-1 beta[Title/Abstract])) OR (Interleukin 1 beta[Title/Abstract]) | "Interleukin-1beta"[Title/Abstract] OR "Interleukin-1beta"[Title/Abstract] OR "Catabolin"[Title/Abstract] OR "il 1 beta"[Title/Abstract] OR "interleukin 1 beta"[Title/Abstract] OR "interleukin 1 beta"[Title/Abstract] | 51,499 |
| 40 | "Interleukin-1beta"[Mesh] | "Interleukin-1beta"[MeSH Terms] | 31,505 |
| 39 | ((((((((((((((((((((((Interleukin‐6[Title/Abstract]) OR (Interleukin 6[Title/Abstract])) OR (B Cell Stimulatory Factor-2[Title/Abstract])) OR (B Cell Stimulatory Factor 2[Title/Abstract])) OR (B-Cell Differentiation Factor[Title/Abstract])) OR (B Cell Differentiation Factor[Title/Abstract])) OR (B-Cell Differentiation Factor-2[Title/Abstract])) OR (B Cell Differentiation Factor 2[Title/Abstract])) OR (B-Cell Stimulatory Factor 2[Title/Abstract])) OR (B-Cell Stimulatory Factor-2[Title/Abstract])) OR (BSF-2[Title/Abstract])) OR (Hepatocyte-Stimulating Factor[Title/Abstract])) OR (Hepatocyte Stimulating Factor[Title/Abstract])) OR (Hybridoma Growth Factor[Title/Abstract])) OR (IFN-beta 2[Title/Abstract])) OR (IL-6[Title/Abstract])) OR (IL6[Title/Abstract])) OR (Interferon beta-2[Title/Abstract])) OR (Interferon beta 2[Title/Abstract])) OR (MGI-2[Title/Abstract])) OR (Myeloid Differentiation-Inducing Protein[Title/Abstract])) OR (Myeloid Differentiation Inducing Protein[Title/Abstract])) OR (Plasmacytoma Growth Factor[Title/Abstract]) | "Interleukin-6"[Title/Abstract] OR "Interleukin-6"[Title/Abstract] OR "b cell stimulatory factor 2"[Title/Abstract] OR "b cell stimulatory factor 2"[Title/Abstract] OR "b cell differentiation factor"[Title/Abstract] OR "b cell differentiation factor"[Title/Abstract] OR "b cell differentiation factor 2"[Title/Abstract] OR "b cell differentiation factor 2"[Title/Abstract] OR "b cell stimulatory factor 2"[Title/Abstract] OR "b cell stimulatory factor 2"[Title/Abstract] OR "BSF-2"[Title/Abstract] OR "hepatocyte stimulating factor"[Title/Abstract] OR "hepatocyte stimulating factor"[Title/Abstract] OR "hybridoma growth factor"[Title/Abstract] OR "ifn beta 2"[Title/Abstract] OR "IL-6"[Title/Abstract] OR "IL6"[Title/Abstract] OR "interferon beta 2"[Title/Abstract] OR "interferon beta 2"[Title/Abstract] OR "MGI-2"[Title/Abstract] OR "myeloid differentiation inducing protein"[Title/Abstract] OR "myeloid differentiation inducing protein"[Title/Abstract] OR "plasmacytoma growth factor"[Title/Abstract] | 223,215 |
| 38 | "Interleukin-6"[Mesh] | "Interleukin-6"[MeSH Terms] | 82,831 |
| 37 | ((((((((((((((Ghrelin[Title/Abstract]) OR (Appetite-Regulating Hormone[Title/Abstract])) OR (Appetite Regulating Hormone[Title/Abstract])) OR (Gastric MLTRP[Title/Abstract])) OR (Motilin-Related Peptide[Title/Abstract])) OR (Motilin Related Peptide[Title/Abstract])) OR (GHRL Protein[Title/Abstract])) OR (PpMTLRP[Title/Abstract])) OR (Ghrelin Precursor[Title/Abstract])) OR (Ghrelin-Obestatin Preprohormone[Title/Abstract])) OR (Ghrelin Obestatin Preprohormone[Title/Abstract])) OR (Motilin-Related Peptide Precursor[Title/Abstract])) OR (Motilin Related Peptide Precursor[Title/Abstract])) OR (Ppghrelin[Title/Abstract])) OR (Obestatin[Title/Abstract]) | "Ghrelin"[Title/Abstract] OR "appetite regulating hormone"[Title/Abstract] OR "appetite regulating hormone"[Title/Abstract] OR (("gastrics"[All Fields] OR "stomach"[MeSH Terms] OR "stomach"[All Fields] OR "gastric"[All Fields]) AND "MLTRP"[Title/Abstract]) OR "motilin related peptide"[Title/Abstract] OR "motilin related peptide"[Title/Abstract] OR "ghrl protein"[Title/Abstract] OR "PpMTLRP"[Title/Abstract] OR "ghrelin precursor"[Title/Abstract] OR ("Ghrelin-Obestatin"[All Fields] AND "Preprohormone"[Title/Abstract]) OR ((("ghrelins"[All Fields] OR "ghrl protein human"[Supplementary Concept] OR "ghrl protein human"[All Fields] OR "Ghrelin"[All Fields] OR "Ghrelin"[Supplementary Concept] OR "Ghrelin"[MeSH Terms]) AND ("ghrl protein human"[Supplementary Concept] OR "ghrl protein human"[All Fields] OR "Obestatin"[All Fields] OR "Ghrelin"[Supplementary Concept] OR "Ghrelin"[All Fields] OR "Ghrelin"[MeSH Terms])) AND "Preprohormone"[Title/Abstract]) OR ("Motilin-Related"[All Fields] AND "peptide precursor"[Title/Abstract]) OR ((("Motilin"[Supplementary Concept] OR "Motilin"[All Fields] OR "Motilin"[MeSH Terms] OR "motilins"[All Fields]) AND ("family"[MeSH Terms] OR "family"[All Fields] OR "relation"[All Fields] OR "relatability"[All Fields] OR "relatable"[All Fields] OR "Related"[All Fields] OR "relates"[All Fields] OR "relating"[All Fields] OR "relational"[All Fields] OR "relations"[All Fields])) AND "peptide precursor"[Title/Abstract]) OR "Ppghrelin"[Title/Abstract] OR "Obestatin"[Title/Abstract] | 13,021 |
| 36 | "Ghrelin"[Mesh] | "Ghrelin"[MeSH Terms] | 8,978 |
| 35 | ((((Leptin[Title/Abstract]) OR (Ob Gene Product[Title/Abstract])) OR (Ob Protein[Title/Abstract])) OR (Obese Gene Product[Title/Abstract])) OR (Obese Protein[Title/Abstract]) | "Leptin"[Title/Abstract] OR "ob gene product"[Title/Abstract] OR "ob protein"[Title/Abstract] OR "obese gene product"[Title/Abstract] OR "obese protein"[Title/Abstract] | 43,866 |
| 34 | "Leptin"[Mesh] | "Leptin"[MeSH Terms] | 27,576 |
| 33 | ((((Prolactin[Title/Abstract]) OR (Pituitary Lactogenic Hormone[Title/Abstract])) OR (Pituitary Mammotropic Hormone[Title/Abstract])) OR (Mammotropin[Title/Abstract])) OR (PRL (Prolactin[Title/Abstract])) | "Prolactin"[Title/Abstract] OR "pituitary lactogenic hormone"[Title/Abstract] OR "pituitary mammotropic hormone"[Title/Abstract] OR "Mammotropin"[Title/Abstract] OR ("PRL"[All Fields] AND "Prolactin"[Title/Abstract]) | 46,682 |
| 32 | "Prolactin"[Mesh] | "Prolactin"[MeSH Terms] | 40,982 |
| 31 | (((((((Growth hormone[Title/Abstract]) OR (Pituitary Growth Hormone[Title/Abstract])) OR (Somatotropin[Title/Abstract])) OR (Recombinant Pituitary Growth Hormones[Title/Abstract])) OR (Recombinant Growth Hormones[Title/Abstract])) OR (Recombinant Somatotropins[Title/Abstract])) OR (Recombinant Somatotropin[Title/Abstract])) OR (Recombinant Growth Hormone[Title/Abstract]) | "growth hormone"[Title/Abstract] OR "pituitary growth hormone"[Title/Abstract] OR "Somatotropin"[Title/Abstract] OR (("f8 protein human"[Supplementary Concept] OR "f8 protein human"[All Fields] OR "recombinate"[All Fields] OR "Recombinant"[All Fields] OR "recombinants"[All Fields] OR "recombinated"[All Fields] OR "recombinates"[All Fields] OR "recombination, genetic"[MeSH Terms] OR ("recombination"[All Fields] AND "genetic"[All Fields]) OR "genetic recombination"[All Fields] OR "recombination"[All Fields] OR "recombinations"[All Fields] OR "recombinational"[All Fields] OR "recombinative"[All Fields] OR "recombine"[All Fields] OR "recombined"[All Fields] OR "recombineered"[All Fields] OR "recombineering"[All Fields] OR "recombines"[All Fields] OR "recombining"[All Fields]) AND "pituitary growth hormones"[Title/Abstract]) OR "recombinant growth hormones"[Title/Abstract] OR "recombinant somatotropins"[Title/Abstract] OR "recombinant somatotropin"[Title/Abstract] OR "recombinant growth hormone"[Title/Abstract] | 65,840 |
| 30 | "Growth Hormone"[Mesh] | "Growth Hormone"[MeSH Terms] | 58,267 |
| 29 | Melatonin[Title/Abstract] | "Melatonin"[Title/Abstract] | 34,658 |
| 28 | "Melatonin"[Mesh] | "Melatonin"[MeSH Terms] | 25,791 |
| 27 | (((((((Hydrocortisone[Title/Abstract]) OR (Cortisol[Title/Abstract])) OR (Cortifair[Title/Abstract])) OR (Cortril[Title/Abstract])) OR (Epicortisol[Title/Abstract])) OR (11-Epicortisol[Title/Abstract])) OR (11 Epicortisol[Title/Abstract])) OR (Cortef[Title/Abstract]) | "Hydrocortisone"[Title/Abstract] OR "Cortisol"[Title/Abstract] OR "Cortril"[Title/Abstract] OR "Epicortisol"[Title/Abstract] OR "11-Epicortisol"[Title/Abstract] OR "11-Epicortisol"[Title/Abstract] OR "Cortef"[Title/Abstract] | 96,506 |
| 26 | "Hydrocortisone"[Mesh] | "Hydrocortisone"[MeSH Terms] | 81,838 |
| 25 | (((((((((Adrenocorticotropic hormone[Title/Abstract]) OR (ACTH[Title/Abstract])) OR (Corticotropin[Title/Abstract])) OR (Corticotropin (1-39[Title/Abstract]))) OR (Corticotrophin (1-39[Title/Abstract]))) OR (Adrenocorticotrophic Hormone[Title/Abstract])) OR (Adrenocorticotropin[Title/Abstract])) OR (1-39 ACTH[Title/Abstract])) OR (ACTH (1-39[Title/Abstract]))) OR (Corticotrophin[Title/Abstract]) | "adrenocorticotropic hormone"[Title/Abstract] OR "ACTH"[Title/Abstract] OR "Corticotropin"[Title/Abstract] OR (("adrenocorticotropic hormone"[Supplementary Concept] OR "adrenocorticotropic hormone"[All Fields] OR "Corticotropin"[All Fields] OR "adrenocorticotropic hormone"[MeSH Terms] OR ("Adrenocorticotropic"[All Fields] AND "Hormone"[All Fields]) OR "corticotropins"[All Fields]) AND "1-39"[Title/Abstract]) OR (("adrenocorticotropic hormone"[Supplementary Concept] OR "adrenocorticotropic hormone"[All Fields] OR "Corticotrophin"[All Fields] OR "adrenocorticotropic hormone"[MeSH Terms] OR ("Adrenocorticotropic"[All Fields] AND "Hormone"[All Fields]) OR "corticotrophins"[All Fields]) AND "1-39"[Title/Abstract]) OR "adrenocorticotrophic hormone"[Title/Abstract] OR "Adrenocorticotropin"[Title/Abstract] OR "1 39 acth"[Title/Abstract] OR (("adrenocorticotropic hormone"[Supplementary Concept] OR "adrenocorticotropic hormone"[All Fields] OR "ACTH"[All Fields] OR "adrenocorticotropic hormone"[MeSH Terms] OR ("Adrenocorticotropic"[All Fields] AND "Hormone"[All Fields])) AND "1-39"[Title/Abstract]) OR "Corticotrophin"[Title/Abstract] | 65,893 |
| 24 | "Adrenocorticotropic Hormone"[Mesh] | "Adrenocorticotropic Hormone"[MeSH Terms] | 51,428 |
| 23 | (((((((((((((((Corticotropin‐releasing hormone[Title/Abstract]) OR (CRH[Title/Abstract])) OR (Corticotropin Releasing Hormone[Title/Abstract])) OR (Corticotropin-Releasing Hormone-41[Title/Abstract])) OR (Corticotropin Releasing Hormone 41[Title/Abstract])) OR (CRF-41[Title/Abstract])) OR (ACTH-Releasing Hormone[Title/Abstract])) OR (ACTH Releasing Hormone[Title/Abstract])) OR (CRF[Title/Abstract])) OR (ACTH-Releasing Factor[Title/Abstract])) OR (ACTH Releasing Factor[Title/Abstract])) OR (Corticoliberin[Title/Abstract])) OR (Corticotropin-Releasing Factor[Title/Abstract])) OR (Corticotropin Releasing Factor[Title/Abstract])) OR (Corticotropin-Releasing Factor-41[Title/Abstract])) OR (Corticotropin Releasing Factor 41[Title/Abstract]) | "corticotropin releasing hormone"[Title/Abstract] OR "CRH"[Title/Abstract] OR "corticotropin releasing hormone"[Title/Abstract] OR "corticotropin releasing hormone 41"[Title/Abstract] OR "corticotropin releasing hormone 41"[Title/Abstract] OR "CRF-41"[Title/Abstract] OR "acth releasing hormone"[Title/Abstract] OR "acth releasing hormone"[Title/Abstract] OR "CRF"[Title/Abstract] OR "acth releasing factor"[Title/Abstract] OR "acth releasing factor"[Title/Abstract] OR "Corticoliberin"[Title/Abstract] OR "corticotropin releasing factor"[Title/Abstract] OR "corticotropin releasing factor"[Title/Abstract] OR "corticotropin releasing factor 41"[Title/Abstract] OR "corticotropin releasing factor 41"[Title/Abstract] | 30,097 |
| 22 | "Corticotropin-Releasing Hormone"[Mesh] | "Corticotropin-Releasing Hormone"[MeSH Terms] | 12,800 |
| 21 | (((Neuropeptide Y[Title/Abstract]) OR (Neuropeptide Tyrosine[Title/Abstract])) OR (Neuropeptide Y Like Immunoreactive Peptide[Title/Abstract])) OR (Neuropeptide Y-Like Immunoreactive Peptide[Title/Abstract]) | "neuropeptide y"[Title/Abstract] OR "neuropeptide tyrosine"[Title/Abstract] OR "neuropeptide y like immunoreactive peptide"[Title/Abstract] OR "neuropeptide y like immunoreactive peptide"[Title/Abstract] | 14,939 |
| 20 | "Neuropeptide Y"[Mesh] | "Neuropeptide Y"[MeSH Terms] | 10,969 |
| 19 | (((((((((((Orexins) OR (Hypocretin)) OR (Orexin)) OR (Hypocretins)) OR (Orexin-A)) OR (Orexin A)) OR (Hypocretin-1)) OR (Hypocretin 1)) OR (Orexin-B)) OR (Orexin B)) OR (Hypocretin-2)) OR (Hypocretin 2) | "orexins"[Supplementary Concept] OR "orexins"[All Fields] OR "orexin"[All Fields] OR "hcrt protein human"[Supplementary Concept] OR "hcrt protein human"[All Fields] OR "orexins"[MeSH Terms] OR "orexins"[Supplementary Concept] OR "orexins"[All Fields] OR "hypocretin"[All Fields] OR "hcrt protein human"[Supplementary Concept] OR "hcrt protein human"[All Fields] OR "orexins"[MeSH Terms] OR "hypocretins"[All Fields] OR "orexins"[Supplementary Concept] OR "orexins"[All Fields] OR "orexin"[All Fields] OR "hcrt protein human"[Supplementary Concept] OR "hcrt protein human"[All Fields] OR "orexins"[MeSH Terms] OR "orexins"[Supplementary Concept] OR "orexins"[All Fields] OR "hypocretin"[All Fields] OR "hcrt protein human"[Supplementary Concept] OR "hcrt protein human"[All Fields] OR "orexins"[MeSH Terms] OR "hypocretins"[All Fields] OR "orexins"[Supplementary Concept] OR "orexins"[All Fields] OR "orexin a"[All Fields] OR "orexins"[MeSH Terms] OR "orexins"[Supplementary Concept] OR "orexins"[All Fields] OR "orexin a"[All Fields] OR "orexins"[MeSH Terms] OR "orexins"[Supplementary Concept] OR "orexins"[All Fields] OR "hypocretin 1"[All Fields] OR "orexins"[MeSH Terms] OR "orexins"[Supplementary Concept] OR "orexins"[All Fields] OR "hypocretin 1"[All Fields] OR "orexins"[MeSH Terms] OR "orexins"[Supplementary Concept] OR "orexins"[All Fields] OR "orexin b"[All Fields] OR "orexins"[MeSH Terms] OR "orexins"[Supplementary Concept] OR "orexins"[All Fields] OR "orexin b"[All Fields] OR "orexins"[MeSH Terms] OR "orexins"[Supplementary Concept] OR "orexins"[All Fields] OR "hypocretin 2"[All Fields] OR "orexins"[MeSH Terms] OR "orexins"[Supplementary Concept] OR "orexins"[All Fields] OR "hypocretin 2"[All Fields] OR "orexins"[MeSH Terms] | 7,807 |
| 18 | "Orexins"[Mesh] | "Orexins"[MeSH Terms] | 4,160 |
| 17 | ((((Histamine[Title/Abstract]) OR (Peremin[Title/Abstract])) OR (Histamine Dihydrochloride[Title/Abstract])) OR (Ceplene[Title/Abstract])) OR (Histamine Hydrochloride[Title/Abstract]) | "Histamine"[Title/Abstract] OR "Peremin"[Title/Abstract] OR "histamine dihydrochloride"[Title/Abstract] OR "Ceplene"[Title/Abstract] OR "histamine hydrochloride"[Title/Abstract] | 64,945 |
| 16 | "Histamine"[Mesh] | "Histamine"[MeSH Terms] | 38,746 |
| 15 | (((((((((((((Acetylcholine[Title/Abstract]) OR (Miochol[Title/Abstract])) OR (Acetylcholine Bromide[Title/Abstract])) OR (Bromoacetylcholine[Title/Abstract])) OR (Acetylcholine Chloride[Title/Abstract])) OR (Chloroacetylcholine[Title/Abstract])) OR (Acetylcholine Iodide[Title/Abstract])) OR (Acetylcholine Hydroxide[Title/Abstract])) OR (Acetylcholine Fluoride[Title/Abstract])) OR (Acetylcholine Picrate[Title/Abstract])) OR (Acetylcholine L-Tartrate[Title/Abstract])) OR (Acetylcholine L Tartrate[Title/Abstract])) OR (Acetylcholine Perchlorate[Title/Abstract])) OR (Acetilcolina Cusi[Title/Abstract]) | "Acetylcholine"[Title/Abstract] OR "Miochol"[Title/Abstract] OR "acetylcholine bromide"[Title/Abstract] OR "Bromoacetylcholine"[Title/Abstract] OR "acetylcholine chloride"[Title/Abstract] OR "Chloroacetylcholine"[Title/Abstract] OR "acetylcholine iodide"[Title/Abstract] OR (("acetylcholin"[All Fields] OR "Acetylcholine"[Supplementary Concept] OR "Acetylcholine"[All Fields] OR "Acetylcholine"[MeSH Terms] OR "acetylcholines"[All Fields]) AND "Hydroxide"[Title/Abstract]) OR (("acetylcholin"[All Fields] OR "Acetylcholine"[Supplementary Concept] OR "Acetylcholine"[All Fields] OR "Acetylcholine"[MeSH Terms] OR "acetylcholines"[All Fields]) AND "Fluoride"[Title/Abstract]) OR "acetylcholine picrate"[Title/Abstract] OR (("acetylcholin"[All Fields] OR "Acetylcholine"[Supplementary Concept] OR "Acetylcholine"[All Fields] OR "Acetylcholine"[MeSH Terms] OR "acetylcholines"[All Fields]) AND "L-Tartrate"[Title/Abstract]) OR (("acetylcholin"[All Fields] OR "Acetylcholine"[Supplementary Concept] OR "Acetylcholine"[All Fields] OR "Acetylcholine"[MeSH Terms] OR "acetylcholines"[All Fields]) AND "L-Tartrate"[Title/Abstract]) OR "acetylcholine perchlorate"[Title/Abstract] OR ("Acetilcolina"[All Fields] AND "Cusi"[Title/Abstract]) | 89,624 |
| 14 | "Acetylcholine"[Mesh] | "Acetylcholine"[MeSH Terms] | 51,198 |
| 13 | (Glutamic Acid[Title/Abstract]) OR (Glutamate[Title/Abstract]) | "glutamic acid"[Title/Abstract] OR "Glutamate"[Title/Abstract] | 152,143 |
| 12 | "Glutamic Acid"[Mesh] | "Glutamic Acid"[MeSH Terms] | 46,142 |
| 11 | (((((((((((((((((((Gamma‐aminobutyric acid[Title/Abstract]) OR (GABA[Title/Abstract])) OR (gamma Aminobutyric Acid[Title/Abstract])) OR (4-Aminobutanoic Acid[Title/Abstract])) OR (4 Aminobutanoic Acid[Title/Abstract])) OR (4-Aminobutyric Acid[Title/Abstract])) OR (4 Aminobutyric Acid[Title/Abstract])) OR (Aminalon[Title/Abstract])) OR (Aminalone[Title/Abstract])) OR (gamma-Aminobutyric Acid, Monolithium Salt[Title/Abstract])) OR (gamma Aminobutyric Acid, Monolithium Salt[Title/Abstract])) OR (Lithium GABA[Title/Abstract])) OR (GABA, Lithium[Title/Abstract])) OR (gamma-Aminobutyric Acid, Hydrochloride[Title/Abstract])) OR (Acid, Hydrochloride gamma-Aminobutyric[Title/Abstract])) OR (gamma Aminobutyric Acid, Hydrochloride[Title/Abstract])) OR (Hydrochloride gamma-Aminobutyric Acid[Title/Abstract])) OR (gamma-Aminobutyric Acid, Monosodium Salt[Title/Abstract])) OR (gamma Aminobutyric Acid, Monosodium Salt[Title/Abstract])) OR (Gammalon[Title/Abstract]) | "gamma aminobutyric acid"[Title/Abstract] OR "GABA"[Title/Abstract] OR "gamma aminobutyric acid"[Title/Abstract] OR "4 aminobutanoic acid"[Title/Abstract] OR "4 aminobutanoic acid"[Title/Abstract] OR "4 aminobutyric acid"[Title/Abstract] OR "4 aminobutyric acid"[Title/Abstract] OR "Aminalon"[Title/Abstract] OR "Aminalone"[Title/Abstract] OR (("gamma aminobutyric acid"[Supplementary Concept] OR "gamma aminobutyric acid"[All Fields] OR "gamma aminobutyric acid"[All Fields] OR "gamma aminobutyric acid"[MeSH Terms] OR ("gamma aminobutyric"[All Fields] AND "Acid"[All Fields]) OR ("gamma"[All Fields] AND "Aminobutyric"[All Fields] AND "Acid"[All Fields])) AND "monolithium salt"[Title/Abstract]) OR (("gamma aminobutyric acid"[Supplementary Concept] OR "gamma aminobutyric acid"[All Fields] OR "gamma aminobutyric acid"[All Fields] OR "gamma aminobutyric acid"[MeSH Terms] OR ("gamma aminobutyric"[All Fields] AND "Acid"[All Fields]) OR ("gamma"[All Fields] AND "Aminobutyric"[All Fields] AND "Acid"[All Fields])) AND "monolithium salt"[Title/Abstract]) OR (("Lithium"[Supplementary Concept] OR "Lithium"[All Fields] OR "Lithium"[MeSH Terms] OR "lithiums"[All Fields]) AND "GABA"[Title/Abstract]) OR (("gamma aminobutyric acid"[Supplementary Concept] OR "gamma aminobutyric acid"[All Fields] OR "GABA"[All Fields] OR "gamma aminobutyric acid"[MeSH Terms] OR ("gamma aminobutyric"[All Fields] AND "Acid"[All Fields])) AND "Lithium"[Title/Abstract]) OR ("gamma aminobutyric"[All Fields] AND "acid hydrochloride"[Title/Abstract]) OR ((("acids"[Supplementary Concept] OR "acids"[All Fields] OR "Acid"[All Fields] OR "acids"[MeSH Terms]) AND ("hydrochlorid"[All Fields] OR "Hydrochloride"[All Fields] OR "hydrochlorides"[All Fields])) AND "gamma aminobutyric"[Title/Abstract]) OR ((("gamma rays"[MeSH Terms] OR ("gamma"[All Fields] AND "rays"[All Fields]) OR "gamma rays"[All Fields] OR "gamma"[All Fields] OR "gamma s"[All Fields] OR "gammae"[All Fields] OR "gammas"[All Fields]) AND ("aminobutyrate"[All Fields] OR "Aminobutyric"[All Fields])) AND "acid hydrochloride"[Title/Abstract]) OR (("hydrochlorid"[All Fields] OR "Hydrochloride"[All Fields] OR "hydrochlorides"[All Fields]) AND "gamma aminobutyric acid"[Title/Abstract]) OR ("gamma aminobutyric"[All Fields] AND "acid monosodium salt"[Title/Abstract]) OR ((("gamma rays"[MeSH Terms] OR ("gamma"[All Fields] AND "rays"[All Fields]) OR "gamma rays"[All Fields] OR "gamma"[All Fields] OR "gamma s"[All Fields] OR "gammae"[All Fields] OR "gammas"[All Fields]) AND ("aminobutyrate"[All Fields] OR "Aminobutyric"[All Fields])) AND "acid monosodium salt"[Title/Abstract]) OR "Gammalon"[Title/Abstract] | 75,809 |
| 10 | "gamma-Aminobutyric Acid"[Mesh] | "gamma-Aminobutyric Acid"[MeSH Terms] | 50,345 |
| 9 | (((((((((((((Norepinephrine[Title/Abstract]) OR (NE[Title/Abstract])) OR (NA[Title/Abstract])) OR (Levarterenol[Title/Abstract])) OR (Levonorepinephrine[Title/Abstract])) OR (Noradrenaline[Title/Abstract])) OR (Arterenol[Title/Abstract])) OR (Norepinephrine Hydrochloride[Title/Abstract])) OR (Norepinephrine Bitartrate[Title/Abstract])) OR (Noradrenaline Bitartrate[Title/Abstract])) OR (Levonor[Title/Abstract])) OR (Levophed[Title/Abstract])) OR (Levophed Bitartrate[Title/Abstract])) OR (Noradrénaline tartrate renaudin[Title/Abstract]) | "Norepinephrine"[Title/Abstract] OR "NE"[Title/Abstract] OR "NA"[Title/Abstract] OR "Levarterenol"[Title/Abstract] OR "Levonorepinephrine"[Title/Abstract] OR "Noradrenaline"[Title/Abstract] OR "Arterenol"[Title/Abstract] OR "norepinephrine hydrochloride"[Title/Abstract] OR "norepinephrine bitartrate"[Title/Abstract] OR "noradrenaline bitartrate"[Title/Abstract] OR "Levonor"[Title/Abstract] OR "Levophed"[Title/Abstract] OR "levophed bitartrate"[Title/Abstract] OR ((("norepinephrin"[All Fields] OR "Norepinephrine"[Supplementary Concept] OR "Norepinephrine"[All Fields] OR "noradrenalin"[All Fields] OR "Norepinephrine"[MeSH Terms] OR "Noradrenaline"[All Fields] OR "norepinephrines"[All Fields]) AND ("tartrates"[Supplementary Concept] OR "tartrates"[All Fields] OR "tartrate"[All Fields] OR "tartaric acid"[Supplementary Concept] OR "tartaric acid"[All Fields] OR "tartrates"[MeSH Terms])) AND "renaudin"[Title/Abstract]) | 330,450 |
| 8 | "Norepinephrine"[Mesh] | "Norepinephrine"[MeSH Terms] | 88,745 |
| 7 | ((((((((Dopamine[Title/Abstract]) OR (DA[Title/Abstract])) OR ("3,4-Dihydroxyphenethylamine"[Title/Abstract])) OR ("3,4 Dihydroxyphenethylamine"[Title/Abstract])) OR (Hydroxytyramine[Title/Abstract])) OR ("4-(2-Aminoethyl)-1,2-benzenediol"[Title/Abstract])) OR (Dopamine Hydrochloride[Title/Abstract])) OR (Hydrochloride, Dopamine[Title/Abstract])) OR (Intropin[Title/Abstract]) | "Dopamine"[Title/Abstract] OR "DA"[Title/Abstract] OR "3 4 dihydroxyphenethylamine"[Title/Abstract] OR "3 4 dihydroxyphenethylamine"[Title/Abstract] OR "Hydroxytyramine"[Title/Abstract] OR "dopamine hydrochloride"[Title/Abstract] OR "hydrochloride dopamine"[Title/Abstract] OR "Intropin"[Title/Abstract] | 224,116 |
| 6 | "Dopamine"[Mesh] | "Dopamine"[MeSH Terms] | 85,820 |
| 5 | (((((((Serotonin[Title/Abstract]) OR (5-HT[Title/Abstract])) OR (5-Hydroxytryptamine[Title/Abstract])) OR (5 Hydroxytryptamine[Title/Abstract])) OR ("3-(2-Aminoethyl)-1H-indol-5-ol"[Title/Abstract])) OR (Enteramine[Title/Abstract])) OR (Hippophaine[Title/Abstract])) OR (Hydroxytryptamine[Title/Abstract]) | "Serotonin"[Title/Abstract] OR "5-HT"[Title/Abstract] OR "5-Hydroxytryptamine"[Title/Abstract] OR "5-Hydroxytryptamine"[Title/Abstract] OR "Enteramine"[Title/Abstract] OR "Hippophaine"[Title/Abstract] OR "Hydroxytryptamine"[Title/Abstract] | 136,626 |
| 4 | "Serotonin"[Mesh] | "Serotonin"[MeSH Terms] | 73,217 |
| 3 | ("Sleep Initiation and Maintenance Disorders"[Mesh]) OR (((((((((((((((((((((("Sleep Initiation and Maintenance Disorders"[Title/Abstract]) OR ("Disorders of Initiating and Maintaining Sleep"[Title/Abstract])) OR (DIMS[Title/Abstract])) OR (Sleeplessness[Title/Abstract])) OR (insomnia Disorder[Title/Abstract])) OR (Insomnia Disorders[Title/Abstract])) OR (Insomnia[Title/Abstract])) OR (Insomnias[Title/Abstract])) OR (Chronic Insomnia[Title/Abstract])) OR (Insomnia, Chronic[Title/Abstract])) OR (Early Awakening[Title/Abstract])) OR (Awakening, Early[Title/Abstract])) OR (Nonorganic Insomnia[Title/Abstract])) OR (Insomnia, Nonorganic[Title/Abstract])) OR (Primary Insomnia[Title/Abstract])) OR (Insomnia, Primary[Title/Abstract])) OR (Psychophysiological Insomnia[Title/Abstract])) OR (Insomnia, Psychophysiological[Title/Abstract])) OR (Sleep Initiation Dysfunction[Title/Abstract])) OR (Dysfunction, Sleep Initiation[Title/Abstract])) OR (Dysfunctions, Sleep Initiation[Title/Abstract])) OR (Sleep Initiation Dysfunctions[Title/Abstract])) | "Sleep Initiation and Maintenance Disorders"[MeSH Terms] OR ("Sleep Initiation and Maintenance Disorders"[Title/Abstract] OR "Disorders of Initiating and Maintaining Sleep"[Title/Abstract] OR "DIMS"[Title/Abstract] OR "Sleeplessness"[Title/Abstract] OR "insomnia disorder"[Title/Abstract] OR "insomnia disorders"[Title/Abstract] OR "Insomnia"[Title/Abstract] OR "Insomnias"[Title/Abstract] OR "chronic insomnia"[Title/Abstract] OR "insomnia chronic"[Title/Abstract] OR "early awakening"[Title/Abstract] OR "awakening early"[Title/Abstract] OR "nonorganic insomnia"[Title/Abstract] OR (("insomnia s"[All Fields] OR "Sleep Initiation and Maintenance Disorders"[MeSH Terms] OR ("Sleep"[All Fields] AND "Initiation"[All Fields] AND "maintenance"[All Fields] AND "Disorders"[All Fields]) OR "Sleep Initiation and Maintenance Disorders"[All Fields] OR "Insomnia"[All Fields] OR "Insomnias"[All Fields]) AND "Nonorganic"[Title/Abstract]) OR "primary insomnia"[Title/Abstract] OR "insomnia primary"[Title/Abstract] OR "psychophysiological insomnia"[Title/Abstract] OR "insomnia psychophysiological"[Title/Abstract] OR "sleep initiation dysfunction"[Title/Abstract] OR (("dysfunctional"[All Fields] OR "dysfunctionals"[All Fields] OR "dysfunctioning"[All Fields] OR "Dysfunctions"[All Fields] OR "physiopathology"[MeSH Subheading] OR "physiopathology"[All Fields] OR "Dysfunction"[All Fields]) AND "sleep initiation"[Title/Abstract]) OR (("dysfunctional"[All Fields] OR "dysfunctionals"[All Fields] OR "dysfunctioning"[All Fields] OR "Dysfunctions"[All Fields] OR "physiopathology"[MeSH Subheading] OR "physiopathology"[All Fields] OR "Dysfunction"[All Fields]) AND "sleep initiation"[Title/Abstract]) OR ((("Sleep"[MeSH Terms] OR "Sleep"[All Fields] OR "sleeping"[All Fields] OR "sleeps"[All Fields] OR "sleep s"[All Fields]) AND ("initial"[All Fields] OR "initially"[All Fields] OR "initials"[All Fields] OR "initiate"[All Fields] OR "initiated"[All Fields] OR "initiates"[All Fields] OR "initiating"[All Fields] OR "Initiation"[All Fields] OR "initiations"[All Fields] OR "initiator"[All Fields] OR "initiators"[All Fields])) AND "Dysfunctions"[Title/Abstract])) | 45,119 |
| 2 | ((((((((((((((((((((("Sleep Initiation and Maintenance Disorders"[Title/Abstract]) OR ("Disorders of Initiating and Maintaining Sleep"[Title/Abstract])) OR (DIMS[Title/Abstract])) OR (Sleeplessness[Title/Abstract])) OR (insomnia Disorder[Title/Abstract])) OR (Insomnia Disorders[Title/Abstract])) OR (Insomnia[Title/Abstract])) OR (Insomnias[Title/Abstract])) OR (Chronic Insomnia[Title/Abstract])) OR (Insomnia, Chronic[Title/Abstract])) OR (Early Awakening[Title/Abstract])) OR (Awakening, Early[Title/Abstract])) OR (Nonorganic Insomnia[Title/Abstract])) OR (Insomnia, Nonorganic[Title/Abstract])) OR (Primary Insomnia[Title/Abstract])) OR (Insomnia, Primary[Title/Abstract])) OR (Psychophysiological Insomnia[Title/Abstract])) OR (Insomnia, Psychophysiological[Title/Abstract])) OR (Sleep Initiation Dysfunction[Title/Abstract])) OR (Dysfunction, Sleep Initiation[Title/Abstract])) OR (Dysfunctions, Sleep Initiation[Title/Abstract])) OR (Sleep Initiation Dysfunctions[Title/Abstract]) | "Sleep Initiation and Maintenance Disorders"[Title/Abstract] OR "Disorders of Initiating and Maintaining Sleep"[Title/Abstract] OR "DIMS"[Title/Abstract] OR "Sleeplessness"[Title/Abstract] OR "insomnia disorder"[Title/Abstract] OR "insomnia disorders"[Title/Abstract] OR "Insomnia"[Title/Abstract] OR "Insomnias"[Title/Abstract] OR "chronic insomnia"[Title/Abstract] OR "insomnia chronic"[Title/Abstract] OR "early awakening"[Title/Abstract] OR "awakening early"[Title/Abstract] OR "nonorganic insomnia"[Title/Abstract] OR (("insomnia s"[All Fields] OR "Sleep Initiation and Maintenance Disorders"[MeSH Terms] OR ("Sleep"[All Fields] AND "Initiation"[All Fields] AND "maintenance"[All Fields] AND "Disorders"[All Fields]) OR "Sleep Initiation and Maintenance Disorders"[All Fields] OR "Insomnia"[All Fields] OR "Insomnias"[All Fields]) AND "Nonorganic"[Title/Abstract]) OR "primary insomnia"[Title/Abstract] OR "insomnia primary"[Title/Abstract] OR "psychophysiological insomnia"[Title/Abstract] OR "insomnia psychophysiological"[Title/Abstract] OR "sleep initiation dysfunction"[Title/Abstract] OR (("dysfunctional"[All Fields] OR "dysfunctionals"[All Fields] OR "dysfunctioning"[All Fields] OR "Dysfunctions"[All Fields] OR "physiopathology"[MeSH Subheading] OR "physiopathology"[All Fields] OR "Dysfunction"[All Fields]) AND "sleep initiation"[Title/Abstract]) OR (("dysfunctional"[All Fields] OR "dysfunctionals"[All Fields] OR "dysfunctioning"[All Fields] OR "Dysfunctions"[All Fields] OR "physiopathology"[MeSH Subheading] OR "physiopathology"[All Fields] OR "Dysfunction"[All Fields]) AND "sleep initiation"[Title/Abstract]) OR ((("Sleep"[MeSH Terms] OR "Sleep"[All Fields] OR "sleeping"[All Fields] OR "sleeps"[All Fields] OR "sleep s"[All Fields]) AND ("initial"[All Fields] OR "initially"[All Fields] OR "initials"[All Fields] OR "initiate"[All Fields] OR "initiated"[All Fields] OR "initiates"[All Fields] OR "initiating"[All Fields] OR "Initiation"[All Fields] OR "initiations"[All Fields] OR "initiator"[All Fields] OR "initiators"[All Fields])) AND "Dysfunctions"[Title/Abstract]) | 39,898 |
| 1 | "Sleep Initiation and Maintenance Disorders"[Mesh] | "Sleep Initiation and Maintenance Disorders"[MeSH Terms] | 21,200 |

**Table S2**. Search strategy used for Web of Science (WOS) database

| **Search number** | **Search Query** | **Results** |
| --- | --- | --- |
| 1 | TS=("Sleep Initiation and Maintenance Disorders" OR "Disorders of Initiating and Maintaining Sleep" OR DIMS OR Sleeplessness OR "insomnia Disorder" OR "Insomnia Disorders" OR Insomnia OR Insomnias OR "Chronic Insomnia" OR "Insomnia, Chronic" OR "Early Awakening" OR "Awakening, Early" OR "Nonorganic Insomnia" OR "Insomnia, Nonorganic" OR "Primary Insomnia" OR "Insomnia, Primary" OR "Psychophysiological Insomnia" OR "Insomnia, Psychophysiological" OR "Sleep Initiation Dysfunction" OR "Dysfunction, Sleep Initiation" OR "Dysfunctions, Sleep Initiation" OR "Sleep Initiation Dysfunctions") | 129227 |
| 2 | TS=("neurotransmit*" OR "Serotonin" OR "5-HT" OR "5-Hydroxytryptamine" OR "5 Hydroxytryptamine" OR "Enteramine" OR "Hippophaine" OR "Hydroxytryptamine" OR "Dopamine" OR "DA" OR "3,4-Dihydroxyphenethylamine" OR "3,4 Dihydroxyphenethylamine" OR "Hydroxytyramine" OR "Dopamine Hydrochloride" OR "Hydrochloride, Dopamine" OR "Intropin" OR "Norepinephrine" OR "NE" OR "NA" OR "Levarterenol" OR "Levonorepinephrine" OR "Noradrenaline" OR "Arterenol" OR "Norepinephrine Hydrochloride" OR "Norepinephrine Bitartrate" OR "Noradrenaline Bitartrate" OR "Levonor" OR "Levophed" OR "Levophed Bitartrate" OR "Noradrénaline tartrate renaudin" OR "Gamma-aminobutyric acid" OR "GABA" OR "gamma Aminobutyric Acid" OR "4-Aminobutanoic Acid" OR "4 Aminobutanoic Acid" OR "4-Aminobutyric Acid" OR "4 Aminobutyric Acid" OR "Aminalon" OR "Aminalone" OR "gamma-Aminobutyric Acid, Monolithium Salt" OR "gamma Aminobutyric Acid, Monolithium Salt" OR "Lithium GABA" OR "GABA, Lithium" OR "gamma-Aminobutyric Acid, Hydrochloride" OR "Acid, Hydrochloride gamma-Aminobutyric" OR "gamma Aminobutyric Acid, Hydrochloride" OR "Hydrochloride gamma-Aminobutyric Acid" OR "gamma-Aminobutyric Acid, Monosodium Salt" OR "gamma Aminobutyric Acid, Monosodium Salt" OR "Gammalon" OR "Glutamic Acid" OR "Glutamate" OR "Acetylcholine" OR "Miochol" OR "Acetylcholine Bromide" OR "Bromoacetylcholine" OR "Acetylcholine Chloride" OR "Chloroacetylcholine" OR "Acetylcholine Iodide" OR "Acetylcholine Hydroxide" OR "Acetylcholine Fluoride" OR "Acetylcholine Picrate" OR "Acetylcholine L-Tartrate" OR "Acetylcholine L Tartrate" OR "Acetylcholine Perchlorate" OR "Acetilcolina Cusi" OR "Histamine" OR "Peremin" OR "Histamine Dihydrochloride" OR "Ceplene" OR "Histamine Hydrochloride" OR "Orexins" OR "Hypocretin" OR "Orexin" OR "Hypocretins" OR "Orexin-A" OR "Orexin A" OR "Hypocretin-1" OR "Hypocretin 1" OR "Orexin-B" OR "Orexin B" OR "Hypocretin-2" OR "Hypocretin 2" OR "Neuropeptide Y" OR "Neuropeptide Tyrosine" OR "Neuropeptide Y Like Immunoreactive Peptide" OR "Neuropeptide Y-Like Immunoreactive Peptide" OR "hormone*" OR "Corticotropin-releasing hormone" OR "CRH" OR "Corticotropin Releasing Hormone" OR "Corticotropin-Releasing Hormone-41" OR "Corticotropin Releasing Hormone 41" OR "CRF-41" OR "ACTH-Releasing Hormone" OR "ACTH Releasing Hormone" OR "CRF" OR "ACTH-Releasing Factor" OR "ACTH Releasing Factor" OR "Corticoliberin" OR "Corticotropin-Releasing Factor" OR "Corticotropin Releasing Factor" OR "Corticotropin-Releasing Factor-41" OR "Corticotropin Releasing Factor 41" OR "Adrenocorticotropic hormone" OR "ACTH" OR "Corticotropin" OR "Corticotropin (1-39)" OR "Corticotrophin (1-39)" OR "Adrenocorticotrophic Hormone" OR "Adrenocorticotropin" OR "1-39 ACTH" OR "ACTH (1-39)" OR "Corticotrophin" OR "Hydrocortisone" OR "Cortisol" OR "Cortifair" OR "Cortril" OR "Epicortisol" OR "11-Epicortisol" OR "11 Epicortisol" OR "Cortef" OR "Melatonin" OR "Growth hormone" OR "Pituitary Growth Hormone" OR "Somatotropin" OR "Recombinant Pituitary Growth Hormones" OR "Recombinant Growth Hormones" OR "Recombinant Somatotropins" OR "Recombinant Somatotropin" OR "Recombinant Growth Hormone" OR "Prolactin" OR "Pituitary Lactogenic Hormone" OR "Pituitary Mammotropic Hormone" OR "Mammotropin" OR "PRL (Prolactin)" OR "Leptin" OR "Ob Gene Product" OR "Ob Protein" OR "Obese Gene Product" OR "Obese Protein" OR "Ghrelin" OR "Appetite-Regulating Hormone" OR "Appetite Regulating Hormone" OR "Gastric MLTRP" OR "Motilin-Related Peptide" OR "Motilin Related Peptide" OR "GHRL Protein" OR "PpMTLRP" OR "Ghrelin Precursor" OR "Ghrelin-Obestatin Preprohormone" OR "Ghrelin Obestatin Preprohormone" OR "Motilin-Related Peptide Precursor" OR "Motilin Related Peptide Precursor" OR "Ppghrelin" OR "Obestatin" OR "cytokine*" OR "Interleukin-6" OR "Interleukin 6" OR "B Cell Stimulatory Factor-2" OR "B Cell Stimulatory Factor 2" OR "B-Cell Differentiation Factor" OR "B Cell Differentiation Factor" OR "B-Cell Differentiation Factor-2" OR "B Cell Differentiation Factor 2" OR "B-Cell Stimulatory Factor 2" OR "B-Cell Stimulatory Factor-2" OR "BSF-2" OR "Hepatocyte-Stimulating Factor" OR "Hepatocyte Stimulating Factor" OR "Hybridoma Growth Factor" OR "IFN-beta 2" OR "IL-6" OR "IL6" OR "Interferon beta-2" OR "Interferon beta 2" OR "MGI-2" OR "Myeloid Differentiation-Inducing Protein" OR "Myeloid Differentiation Inducing Protein" OR "Plasmacytoma Growth Factor" OR "Interleukin-1beta" OR "Interleukin 1beta" OR "Catabolin" OR "IL-1 beta" OR "Interleukin-1 beta" OR "Interleukin 1 beta" OR "Tumor Necrosis Factor-alpha" OR "Tumor Necrosis Factor alpha" OR "TNF-alpha" OR "Tumor Necrosis Factor" OR "Tumor Necrosis Factor Ligand Superfamily Member 2" OR "TNFalpha" OR "Cachectin-Tumor Necrosis Factor" OR "Cachectin Tumor Necrosis Factor" OR "Cachectin" OR "Interleukin-17" OR "CTLA-8" OR "IL-17" OR "Interleukin 17" OR "Interleukin-17B" OR "Interleukin 17B" OR "IL-17B" OR "Interleukin-17A" OR "Interleukin 17A" OR "CTLA8" OR "Cytotoxic T lymphocyte-Associated Antigen 8" OR "Cytotoxic T lymphocyte Associated Antigen 8" OR "IL-17A" OR "Interleukin-17C" OR "Interleukin 17C" OR "Cytokine CX2" OR "IL-17C" OR "Interleukin-17E" OR "Interleukin 17E" OR "IL-17E" OR "IL 17E" OR "Interleukin-25" OR "Interleukin 25" OR "Interleukin-17F" OR "Interleukin 17F" OR "Cytokine ML-1" OR "Cytokine ML 1" OR "IL-17F" OR "Interleukin-8" OR "Interleukin 8" OR "IL-8" OR "IL8" OR "Monocyte-Derived Neutrophil Chemotactic Factor" OR "Neutrophil Activation Factor" OR "Lymphocyte-Derived Neutrophil-Activating Peptide" OR "Monocyte-Derived Neutrophil-Activating Peptide" OR "Alveolar Macrophage Chemotactic Factor-I" OR "Alveolar Macrophage Chemotactic Factor I" OR "Anionic Neutrophil-Activating Peptide" OR "Anionic Neutrophil Activating Peptide" OR "Chemokine CXCL8" OR "CXCL8 Chemokines" OR "Macrophage-Derived Chemotactic Factor" OR "Neutrophil Chemotactic Factor" OR "CXCL8 Chemokine" OR "Granulocyte Chemotactic Peptide-Interleukin-8" OR "Granulocyte Chemotactic Peptide Interleukin 8" OR "AMCF-I" OR "Interleukin-10" OR "Interleukin 10" OR "CSIF-10" OR "Cytokine Synthesis Inhibitory Factor" OR "IL-10" OR "IL10" OR "Interferon-gamma" OR "Interferon Type II" OR "Immune Interferon" OR "Type II Interferon" OR "gamma-Interferon" OR "Transforming Growth Factor beta" OR "Platelet Transforming Growth Factor" OR "Bone-Derived Transforming Growth Factor" OR "Bone Derived Transforming Growth Factor" OR "TGF-beta" OR "TGFbeta" OR "Milk Growth Factor" OR "C-reactive protein" OR "C Reactive Protein" OR "High Sensitivity C-Reactive Protein" OR "High Sensitivity C Reactive Protein" OR "hs-CRP" OR "hsCRP" OR "Serum Amyloid A Protein" OR "Amyloid A Precursor" OR "Amyloid A Protein-Related Serum Component" OR "Amyloid A Protein Related Serum Component" OR "Amyloid Protein AA Precursor" OR "Amyloid Protein SAA" OR "Amyloid Serum Protein SAA" OR "Amyloid-Related Serum Protein" OR "SAA" OR "Serum A Related Protein" OR "Serum Amyloid A" OR "Serum Amyloid Protein A" OR "Amyloid A Protein" OR "Amyloid Fibril Protein AA" OR "Amyloid Protein AA" OR "neurotrophic*" OR "Brain-derived neurotrophic factor" OR "BDNF" OR "Brain Derived Neurotrophic Factor" OR "Nerve growth factor" OR "NGF" OR "Nerve Growth Factor 1" OR "Nerve Growth Factor beta Subunit" OR "beta-Nerve Growth Factor" OR "beta Nerve Growth Factor" OR "NGF-1beta" OR "NGF 1beta" OR "Nerve Growth Factor alpha Subunit" OR "Nerve Growth Factor gamma Subunit" OR "Glial cell line-derived neurotrophic factor" OR "GDNF" OR "Glial Cell Line Derived Neurotrophic Factor" OR "Glial Cell-Line Derived Neurotrophic Factor" OR "Glial Cell-Derived Neurotrophic Factor" OR "Glial Cell Derived Neurotrophic Factor" OR "Glial-Derived Neurotrophic Factor" OR "Glial Derived Neurotrophic Factor" OR "GDNF Growth Factor" OR "oxidative stress*" OR "Malondialdehyde" OR "MDA" OR "Malonaldehyde" OR "Malonylaldehyde" OR "Malonyldialdehyde" OR "Propanedial" OR "Sodium Malondialdehyde" OR "Superoxide dismutase" OR "SOD" OR "Erythrocuprein" OR "Hemocuprein" OR "Cu-Superoxide Dismutase" OR "Cu Superoxide Dismutase" OR "Fe-Zn Superoxide Dismutase" OR "Fe Zn Superoxide Dismutase" OR "Cobalt Superoxide Dismutase" OR "Mn-Superoxide Dismutase" OR "Mn Superoxide Dismutase" OR "Mn-SOD" OR "Mn SOD" OR "Manganese Superoxide Dismutase" OR "Ag-Zn Superoxide Dismutase" OR "Ag Zn Superoxide Dismutase" OR "Iron Superoxide Dismutase" OR "Fe-Superoxide Dismutase" OR "Fe Superoxide Dismutase" OR "Glutathione" OR "GSH" OR "Nitric oxide" OR "NO" OR "Nitrogen Monoxide" OR "Mononitrogen Monoxide" OR "Endogenous Nitrate Vasodilator" OR "Endothelium-Derived Nitric Oxide" OR "metabolic*" OR "Insulin" OR "Regular Insulin" OR "Soluble Insulin" OR "Iletin" OR "Insulin A Chain" OR "Novolin" OR "Sodium Insulin" OR "Insulin B Chain" OR "Glucose" OR "D-Glucose" OR "D Glucose" OR "Dextrose" OR "Glucose Monohydrate" OR "Anhydrous Dextrose" OR "Triglycerides" OR "Triglyceride" OR "Triacylglycerols" OR "Triacylglycerol" OR "Lipoproteins" OR "Lipoproteins, LDL" OR "LDL" OR "LDL Lipoproteins" OR "beta-Lipoprotein" OR "beta Lipoprotein" OR "Low-Density Lipoprotein" OR "Low Density Lipoprotein" OR "beta-Lipoproteins" OR "beta Lipoproteins" OR "Low-Density Lipoproteins" OR "Low Density Lipoproteins" OR "LDL-1" OR "LDL(1)" OR "LDL1" OR "Low-Density Lipoprotein 1" OR "Low Density Lipoprotein 1" OR "LDL-2" OR "LDL(2)" OR "LDL2" OR "Low-Density Lipoprotein 2" OR "Low Density Lipoprotein 2" OR "Amyloid beta-Peptides" OR "Amyloid beta Peptides" OR "Alzheimer beta-Protein" OR "Alzheimer beta Protein" OR "Alzheimer's ABP" OR "Alzheimer ABP" OR "Alzheimers ABP" OR "Alzheimer's Amyloid Fibril Protein" OR "Amyloid AD-AP" OR "Amyloid AD AP" OR "Amyloid beta-Peptide" OR "Amyloid beta Peptide" OR "Amyloid beta-Protein" OR "Amyloid beta Protein" OR "Amyloid beta-Proteins" OR "Amyloid beta Proteins" OR "Amyloid Protein A4" OR "beta Amyloid" OR "beta-Amyloid Protein" OR "beta Amyloid Protein" OR "tau Proteins" OR "tau Protein" OR "Fatty Acids, Volatile" OR "Volatile Fatty Acids" OR "Short-Chain Fatty Acids" OR "Short-Chain Fatty Acid" OR "Short Chain Fatty Acid" OR "Volatile Fatty Acid" OR "Tryptophan" OR "Levotryptophan" OR "L-Tryptophan" OR "L Tryptophan" OR "Tryptophan Metabolism Alterations" OR "Tryptan" OR "Tryptacin" OR "Trofan" OR "Ardeydorm" OR "Ardeytropin" OR "L-Tryptophan-ratiopharm" OR "L Tryptophan ratiopharm" OR "ratio-Tryptophan" OR "ratio Tryptophan" OR "Lyphan" OR "Naturruhe" OR "PMS-Tryptophan" OR "PMS Tryptophan" OR "Optimax" OR "Kynurenine") | 21169609 |
| 3 | #1 AND #2 | 43382 |

**Table S3.** Search strategy used for Cochrane Libiary database

| Search number | Search | Results |
| --- | --- | --- |
| #1 | ("Sleep Initiation and Maintenance Disorders" OR "Disorders of Initiating and Maintaining Sleep" OR DIMS OR Sleeplessness OR "insomnia Disorder" OR "Insomnia Disorders" OR Insomnia OR Insomnias OR "Chronic Insomnia" OR "Insomnia, Chronic" OR "Early Awakening" OR "Awakening, Early" OR "Nonorganic Insomnia" OR "Insomnia, Nonorganic" OR "Primary Insomnia" OR "Insomnia, Primary" OR "Psychophysiological Insomnia" OR "Insomnia, Psychophysiological" OR "Sleep Initiation Dysfunction" OR "Dysfunction, Sleep Initiation" OR "Dysfunctions, Sleep Initiation" OR "Sleep Initiation Dysfunctions"):ti,ab,kw (Word variations have been searched) | 18942 |
| #2 | neurotransmit* OR "Serotonin" OR "5-HT" OR "5-Hydroxytryptamine" OR "5 Hydroxytryptamine" OR "Enteramine" OR "Hippophaine" OR "Hydroxytryptamine" OR "Dopamine" OR "DA" OR "3,4-Dihydroxyphenethylamine" OR "3,4 Dihydroxyphenethylamine" OR "Hydroxytyramine" OR "Dopamine Hydrochloride" OR "Hydrochloride, Dopamine" OR "Intropin" OR "Norepinephrine" OR "NE" OR "NA" OR "Levarterenol" OR "Levonorepinephrine" OR "Noradrenaline" OR "Arterenol" OR "Norepinephrine Hydrochloride" OR "Norepinephrine Bitartrate" OR "Noradrenaline Bitartrate" OR "Levonor" OR "Levophed" OR "Levophed Bitartrate" OR "Noradrénaline tartrate renaudin" OR "Gamma-aminobutyric acid" OR "GABA" OR "gamma Aminobutyric Acid" OR "4-Aminobutanoic Acid" OR "4 Aminobutanoic Acid" OR "4-Aminobutyric Acid" OR "4 Aminobutyric Acid" OR "Aminalon" OR "Aminalone" OR "gamma-Aminobutyric Acid, Monolithium Salt" OR "gamma Aminobutyric Acid, Monolithium Salt" OR "Lithium GABA" OR "GABA, Lithium" OR "gamma-Aminobutyric Acid, Hydrochloride" OR "Acid, Hydrochloride gamma-Aminobutyric" OR "gamma Aminobutyric Acid, Hydrochloride" OR "Hydrochloride gamma-Aminobutyric Acid" OR "gamma-Aminobutyric Acid, Monosodium Salt" OR "gamma Aminobutyric Acid, Monosodium Salt" OR "Gammalon" OR "Glutamic Acid" OR "Glutamate" OR "Acetylcholine" OR "Miochol" OR "Acetylcholine Bromide" OR "Bromoacetylcholine" OR "Acetylcholine Chloride" OR "Chloroacetylcholine" OR "Acetylcholine Iodide" OR "Acetylcholine Hydroxide" OR "Acetylcholine Fluoride" OR "Acetylcholine Picrate" OR "Acetylcholine L-Tartrate" OR "Acetylcholine L Tartrate" OR "Acetylcholine Perchlorate" OR "Acetilcolina Cusi" OR "Histamine" OR "Peremin" OR "Histamine Dihydrochloride" OR "Ceplene" OR "Histamine Hydrochloride" OR "Orexins" OR "Hypocretin" OR "Orexin" OR "Hypocretins" OR "Orexin-A" OR "Orexin A" OR "Hypocretin-1" OR "Hypocretin 1" OR "Orexin-B" OR "Orexin B" OR "Hypocretin-2" OR "Hypocretin 2" OR "Neuropeptide Y" OR "Neuropeptide Tyrosine" OR "Neuropeptide Y Like Immunoreactive Peptide" OR "Neuropeptide Y-Like Immunoreactive Peptide" | 123982 |
| #3 | hormone* OR "Corticotropin-releasing hormone" OR "CRH" OR "Corticotropin Releasing Hormone" OR "Corticotropin-Releasing Hormone-41" OR "Corticotropin Releasing Hormone 41" OR "CRF-41" OR "ACTH-Releasing Hormone" OR "ACTH Releasing Hormone" OR "CRF" OR "ACTH-Releasing Factor" OR "ACTH Releasing Factor" OR "Corticoliberin" OR "Corticotropin-Releasing Factor" OR "Corticotropin Releasing Factor" OR "Corticotropin-Releasing Factor-41" OR "Corticotropin Releasing Factor 41" OR "Adrenocorticotropic hormone" OR "ACTH" OR "Corticotropin" OR "Corticotropin (1-39)" OR "Corticotrophin (1-39)" OR "Adrenocorticotrophic Hormone" OR "Adrenocorticotropin" OR "1-39 ACTH" OR "ACTH (1-39)" OR "Corticotrophin" OR "Hydrocortisone" OR "Cortisol" OR "Cortifair" OR "Cortril" OR "Epicortisol" OR "11-Epicortisol" OR "11 Epicortisol" OR "Cortef" OR "Melatonin" OR "Growth hormone" OR "Pituitary Growth Hormone" OR "Somatotropin" OR "Recombinant Pituitary Growth Hormones" OR "Recombinant Growth Hormones" OR "Recombinant Somatotropins" OR "Recombinant Somatotropin" OR "Recombinant Growth Hormone" OR "Prolactin" OR "Pituitary Lactogenic Hormone" OR "Pituitary Mammotropic Hormone" OR "Mammotropin" OR "PRL (Prolactin)" OR "Leptin" OR "Ob Gene Product" OR "Ob Protein" OR "Obese Gene Product" OR "Obese Protein" OR "Ghrelin" OR "Appetite-Regulating Hormone" OR "Appetite Regulating Hormone" OR "Gastric MLTRP" OR "Motilin-Related Peptide" OR "Motilin Related Peptide" OR "GHRL Protein" OR "PpMTLRP" OR "Ghrelin Precursor" OR "Ghrelin-Obestatin Preprohormone" OR "Ghrelin Obestatin Preprohormone" OR "Motilin-Related Peptide Precursor" OR "Motilin Related Peptide Precursor" OR "Ppghrelin" OR "Obestatin" | 89730 |
| #4 | cytokine* OR "Interleukin-6" OR "Interleukin 6" OR "B Cell Stimulatory Factor-2" OR "B Cell Stimulatory Factor 2" OR "B-Cell Differentiation Factor" OR "B Cell Differentiation Factor" OR "B-Cell Differentiation Factor-2" OR "B Cell Differentiation Factor 2" OR "B-Cell Stimulatory Factor 2" OR "B-Cell Stimulatory Factor-2" OR "BSF-2" OR "Hepatocyte-Stimulating Factor" OR "Hepatocyte Stimulating Factor" OR "Hybridoma Growth Factor" OR "IFN-beta 2" OR "IL-6" OR "IL6" OR "Interferon beta-2" OR "Interferon beta 2" OR "MGI-2" OR "Myeloid Differentiation-Inducing Protein" OR "Myeloid Differentiation Inducing Protein" OR "Plasmacytoma Growth Factor" OR "Interleukin-1beta" OR "Interleukin 1beta" OR "Catabolin" OR "IL-1 beta" OR "Interleukin-1 beta" OR "Interleukin 1 beta" OR "Tumor Necrosis Factor-alpha" OR "Tumor Necrosis Factor alpha" OR "TNF-alpha" OR "Tumor Necrosis Factor" OR "Tumor Necrosis Factor Ligand Superfamily Member 2" OR "TNFalpha" OR "Cachectin-Tumor Necrosis Factor" OR "Cachectin Tumor Necrosis Factor" OR "Cachectin" OR "Interleukin-17" OR "CTLA-8" OR "IL-17" OR "Interleukin 17" OR "Interleukin-17B" OR "Interleukin 17B" OR "IL-17B" OR "Interleukin-17A" OR "Interleukin 17A" OR "CTLA8" OR "Cytotoxic T lymphocyte-Associated Antigen 8" OR "Cytotoxic T lymphocyte Associated Antigen 8" OR "IL-17A" OR "Interleukin-17C" OR "Interleukin 17C" OR "Cytokine CX2" OR "IL-17C" OR "Interleukin-17E" OR "Interleukin 17E" OR "IL-17E" OR "IL 17E" OR "Interleukin-25" OR "Interleukin 25" OR "Interleukin-17F" OR "Interleukin 17F" OR "Cytokine ML-1" OR "Cytokine ML 1" OR "IL-17F" OR "Interleukin-8" OR "Interleukin 8" OR "IL-8" OR "IL8" OR "Monocyte-Derived Neutrophil Chemotactic Factor" OR "Neutrophil Activation Factor" OR "Lymphocyte-Derived Neutrophil-Activating Peptide" OR "Monocyte-Derived Neutrophil-Activating Peptide" OR "Alveolar Macrophage Chemotactic Factor-I" OR "Alveolar Macrophage Chemotactic Factor I" OR "Anionic Neutrophil-Activating Peptide" OR "Anionic Neutrophil Activating Peptide" OR "Chemokine CXCL8" OR "CXCL8 Chemokines" OR "Macrophage-Derived Chemotactic Factor" OR "Neutrophil Chemotactic Factor" OR "CXCL8 Chemokine" OR "Granulocyte Chemotactic Peptide-Interleukin-8" OR "Granulocyte Chemotactic Peptide Interleukin 8" OR "AMCF-I" OR "Interleukin-10" OR "Interleukin 10" OR "CSIF-10" OR "Cytokine Synthesis Inhibitory Factor" OR "IL-10" OR "IL10" OR "Interferon-gamma" OR "Interferon Type II" OR "Immune Interferon" OR "Type II Interferon" OR "gamma-Interferon" OR "Transforming Growth Factor beta" OR "Platelet Transforming Growth Factor" OR "Bone-Derived Transforming Growth Factor" OR "Bone Derived Transforming Growth Factor" OR "TGF-beta" OR "TGFbeta" OR "Milk Growth Factor" OR "C-reactive protein" OR "C Reactive Protein" OR "High Sensitivity C-Reactive Protein" OR "High Sensitivity C Reactive Protein" OR "hs-CRP" OR "hsCRP" OR "Serum Amyloid A Protein" OR "Amyloid A Precursor" OR "Amyloid A Protein-Related Serum Component" OR "Amyloid A Protein Related Serum Component" OR "Amyloid Protein AA Precursor" OR "Amyloid Protein SAA" OR "Amyloid Serum Protein SAA" OR "Amyloid-Related Serum Protein" OR "SAA" OR "Serum A Related Protein" OR "Serum Amyloid A" OR "Serum Amyloid Protein A" OR "Amyloid A Protein" OR "Amyloid Fibril Protein AA" OR "Amyloid Protein AA" OR neurotrophic* OR "Brain-derived neurotrophic factor" OR "BDNF" OR "Brain Derived Neurotrophic Factor" OR "Nerve growth factor" OR "NGF" OR "Nerve Growth Factor 1" OR "Nerve Growth Factor beta Subunit" OR "beta-Nerve Growth Factor" OR "beta Nerve Growth Factor" OR "NGF-1beta" OR "NGF 1beta" OR "Nerve Growth Factor alpha Subunit" OR "Nerve Growth Factor gamma Subunit" OR "Glial cell line-derived neurotrophic factor" OR "GDNF" OR "Glial Cell Line Derived Neurotrophic Factor" OR "Glial Cell-Line Derived Neurotrophic Factor" OR "Glial Cell-Derived Neurotrophic Factor" OR "Glial Cell Derived Neurotrophic Factor" OR "Glial-Derived Neurotrophic Factor" OR "Glial Derived Neurotrophic Factor" OR "GDNF Growth Factor" OR (oxidative NEXT stress*) OR "Malondialdehyde" OR "MDA" OR "Malonaldehyde" OR "Malonylaldehyde" OR "Malonyldialdehyde" OR "Propanedial" OR "Sodium Malondialdehyde" OR "Superoxide dismutase" OR "SOD" OR "Erythrocuprein" OR "Hemocuprein" OR "Cu-Superoxide Dismutase" OR "Cu Superoxide Dismutase" OR "Fe-Zn Superoxide Dismutase" OR "Fe Zn Superoxide Dismutase" OR "Cobalt Superoxide Dismutase" OR "Mn-Superoxide Dismutase" OR "Mn Superoxide Dismutase" OR "Mn-SOD" OR "Mn SOD" OR "Manganese Superoxide Dismutase" OR "Ag-Zn Superoxide Dismutase" OR "Ag Zn Superoxide Dismutase" OR "Iron Superoxide Dismutase" OR "Fe-Superoxide Dismutase" OR "Fe Superoxide Dismutase" OR "Glutathione" OR "GSH" OR "Nitric oxide" OR "NO" OR "Nitrogen Monoxide" OR "Mononitrogen Monoxide" OR "Endogenous Nitrate Vasodilator" OR "Endothelium-Derived Nitric Oxide" OR metabolic* OR "Insulin" OR "Regular Insulin" OR "Soluble Insulin" OR "Iletin" OR "Insulin A Chain" OR "Novolin" OR "Sodium Insulin" OR "Insulin B Chain" OR "Glucose" OR "D-Glucose" OR "D Glucose" OR "Dextrose" OR "Glucose Monohydrate" OR "Anhydrous Dextrose" OR "Triglycerides" OR "Triglyceride" OR "Triacylglycerols" OR "Triacylglycerol" OR "Lipoproteins" OR "Lipoproteins, LDL" OR "LDL" OR "LDL Lipoproteins" OR "beta-Lipoprotein" OR "beta Lipoprotein" OR "Low-Density Lipoprotein" OR "Low Density Lipoprotein" OR "beta-Lipoproteins" OR "beta Lipoproteins" OR "Low-Density Lipoproteins" OR "Low Density Lipoproteins" OR "LDL-1" OR "LDL(1)" OR "LDL1" OR "Low-Density Lipoprotein 1" OR "Low Density Lipoprotein 1" OR "LDL-2" OR "LDL(2)" OR "LDL2" OR "Low-Density Lipoprotein 2" OR "Low Density Lipoprotein 2" OR "Amyloid beta-Peptides" OR "Amyloid beta Peptides" OR "Alzheimer beta-Protein" OR "Alzheimer beta Protein" OR "Alzheimer's ABP" OR "Alzheimer ABP" OR "Alzheimers ABP" OR "Alzheimer's Amyloid Fibril Protein" OR "Amyloid AD-AP" OR "Amyloid AD AP" OR "Amyloid beta-Peptide" OR "Amyloid beta Peptide" OR "Amyloid beta-Protein" OR "Amyloid beta Protein" OR "Amyloid beta-Proteins" OR "Amyloid beta Proteins" OR "Amyloid Protein A4" OR "beta Amyloid" OR "beta-Amyloid Protein" OR "beta Amyloid Protein" OR "tau Proteins" OR "tau Protein" OR "Fatty Acids, Volatile" OR "Volatile Fatty Acids" OR "Short-Chain Fatty Acids" OR "Short-Chain Fatty Acid" OR "Short Chain Fatty Acid" OR "Volatile Fatty Acid" OR "Tryptophan" OR "Levotryptophan" OR "L-Tryptophan" OR "L Tryptophan" OR "Tryptophan Metabolism Alterations" OR "Tryptan" OR "Tryptacin" OR "Trofan" OR "Ardeydorm" OR "Ardeytropin" OR "L-Tryptophan-ratiopharm" OR "L Tryptophan ratiopharm" OR "ratio-Tryptophan" OR "ratio Tryptophan" OR "Lyphan" OR "Naturruhe" OR "PMS-Tryptophan" OR "PMS Tryptophan" OR "Optimax" OR "Kynurenine" | 955613 |
| #5 | #2 OR #3 OR #4 | 1049758 |
| #6 | #1 AND #5 | 10273 |

**Table S4.** Search strategy used for EMBASE database

| Search number | Query | Results |
| --- | --- | --- |
| #3 | #1 AND #2 | 45102 |
| #2 | 'neurotransmit*':ab,ti OR 'serotonin':ab,ti OR '5-ht':ab,ti OR '5-hydroxytryptamine':ab,ti OR '5 hydroxytryptamine':ab,ti OR 'enteramine':ab,ti OR 'hippophaine':ab,ti OR 'hydroxytryptamine':ab,ti OR 'dopamine':ab,ti OR 'da':ab,ti OR '3,4-dihydroxyphenethylamine':ab,ti OR '3,4 dihydroxyphenethylamine':ab,ti OR 'hydroxytyramine':ab,ti OR 'dopamine hydrochloride':ab,ti OR 'hydrochloride, dopamine':ab,ti OR 'intropin':ab,ti OR 'norepinephrine':ab,ti OR 'ne':ab,ti OR 'na':ab,ti OR 'levarterenol':ab,ti OR 'levonorepinephrine':ab,ti OR 'noradrenaline':ab,ti OR 'arterenol':ab,ti OR 'norepinephrine hydrochloride':ab,ti OR 'norepinephrine bitartrate':ab,ti OR 'noradrenaline bitartrate':ab,ti OR 'levonor':ab,ti OR 'levophed':ab,ti OR 'levophed bitartrate':ab,ti OR 'noradrénaline tartrate renaudin':ab,ti OR 'gamma-aminobutyric acid':ab,ti OR 'gaba':ab,ti OR 'gamma aminobutyric acid':ab,ti OR '4-aminobutanoic acid':ab,ti OR '4 aminobutanoic acid':ab,ti OR '4-aminobutyric acid':ab,ti OR '4 aminobutyric acid':ab,ti OR 'aminalon':ab,ti OR 'aminalone':ab,ti OR 'gamma-aminobutyric acid, monolithium salt':ab,ti OR 'gamma aminobutyric acid, monolithium salt':ab,ti OR 'lithium gaba':ab,ti OR 'gaba, lithium':ab,ti OR 'gamma-aminobutyric acid, hydrochloride':ab,ti OR 'acid, hydrochloride gamma-aminobutyric':ab,ti OR 'gamma aminobutyric acid, hydrochloride':ab,ti OR 'hydrochloride gamma-aminobutyric acid':ab,ti OR 'gamma-aminobutyric acid, monosodium salt':ab,ti OR 'gamma aminobutyric acid, monosodium salt':ab,ti OR 'gammalon':ab,ti OR 'glutamic acid':ab,ti OR 'glutamate':ab,ti OR 'acetylcholine':ab,ti OR 'miochol':ab,ti OR 'acetylcholine bromide':ab,ti OR 'bromoacetylcholine':ab,ti OR 'acetylcholine chloride':ab,ti OR 'chloroacetylcholine':ab,ti OR 'acetylcholine iodide':ab,ti OR 'acetylcholine hydroxide':ab,ti OR 'acetylcholine fluoride':ab,ti OR 'acetylcholine picrate':ab,ti OR 'acetylcholine l-tartrate':ab,ti OR 'acetylcholine l tartrate':ab,ti OR 'acetylcholine perchlorate':ab,ti OR 'acetilcolina cusi':ab,ti OR 'histamine':ab,ti OR 'peremin':ab,ti OR 'histamine dihydrochloride':ab,ti OR 'ceplene':ab,ti OR 'histamine hydrochloride':ab,ti OR 'orexins':ab,ti OR 'hypocretin':ab,ti OR 'orexin':ab,ti OR 'hypocretins':ab,ti OR 'orexin-a':ab,ti OR 'orexin a':ab,ti OR 'hypocretin-1':ab,ti OR 'hypocretin 1':ab,ti OR 'orexin-b':ab,ti OR 'orexin b':ab,ti OR 'hypocretin-2':ab,ti OR 'hypocretin 2':ab,ti OR 'neuropeptide y':ab,ti OR 'neuropeptide tyrosine':ab,ti OR 'neuropeptide y like immunoreactive peptide':ab,ti OR 'neuropeptide y-like immunoreactive peptide':ab,ti OR 'hormone*':ab,ti OR 'corticotropin-releasing hormone':ab,ti OR 'crh':ab,ti OR 'corticotropin releasing hormone':ab,ti OR 'corticotropin-releasing hormone-41':ab,ti OR 'corticotropin releasing hormone 41':ab,ti OR 'crf-41':ab,ti OR 'acth-releasing hormone':ab,ti OR 'acth releasing hormone':ab,ti OR 'crf':ab,ti OR 'acth-releasing factor':ab,ti OR 'acth releasing factor':ab,ti OR 'corticoliberin':ab,ti OR 'corticotropin-releasing factor':ab,ti OR 'corticotropin releasing factor':ab,ti OR 'corticotropin-releasing factor-41':ab,ti OR 'corticotropin releasing factor 41':ab,ti OR 'adrenocorticotropic hormone':ab,ti OR 'acth':ab,ti OR 'corticotropin':ab,ti OR 'corticotropin (1-39)':ab,ti OR 'corticotrophin (1-39)':ab,ti OR 'adrenocorticotrophic hormone':ab,ti OR 'adrenocorticotropin':ab,ti OR '1-39 acth':ab,ti OR 'acth (1-39)':ab,ti OR 'corticotrophin':ab,ti OR 'hydrocortisone':ab,ti OR 'cortisol':ab,ti OR 'cortifair':ab,ti OR 'cortril':ab,ti OR 'epicortisol':ab,ti OR '11-epicortisol':ab,ti OR '11 epicortisol':ab,ti OR 'cortef':ab,ti OR 'melatonin':ab,ti OR 'growth hormone':ab,ti OR 'pituitary growth hormone':ab,ti OR 'somatotropin':ab,ti OR 'recombinant pituitary growth hormones':ab,ti OR 'recombinant growth hormones':ab,ti OR 'recombinant somatotropins':ab,ti OR 'recombinant somatotropin':ab,ti OR 'recombinant growth hormone':ab,ti OR 'prolactin':ab,ti OR 'pituitary lactogenic hormone':ab,ti OR 'pituitary mammotropic hormone':ab,ti OR 'mammotropin':ab,ti OR 'prl (prolactin)':ab,ti OR 'leptin':ab,ti OR 'ob gene product':ab,ti OR 'ob protein':ab,ti OR 'obese gene product':ab,ti OR 'obese protein':ab,ti OR 'ghrelin':ab,ti OR 'appetite-regulating hormone':ab,ti OR 'appetite regulating hormone':ab,ti OR 'gastric mltrp':ab,ti OR 'motilin-related peptide':ab,ti OR 'motilin related peptide':ab,ti OR 'ghrl protein':ab,ti OR 'ppmltrp':ab,ti OR 'ghrelin precursor':ab,ti OR 'ghrelin-obestatin preprohormone':ab,ti OR 'ghrelin obestatin preprohormone':ab,ti OR 'motilin-related peptide precursor':ab,ti OR 'motilin related peptide precursor':ab,ti OR 'ppghrelin':ab,ti OR 'obestatin':ab,ti OR 'cytokine*':ab,ti OR 'interleukin-6':ab,ti OR 'interleukin 6':ab,ti OR 'b cell stimulatory factor-2':ab,ti OR 'b cell stimulatory factor 2':ab,ti OR 'b-cell differentiation factor':ab,ti OR 'b cell differentiation factor':ab,ti OR 'b-cell differentiation factor-2':ab,ti OR 'b cell differentiation factor 2':ab,ti OR 'b-cell stimulatory factor 2':ab,ti OR 'b-cell stimulatory factor-2':ab,ti OR 'bsf-2':ab,ti OR 'hepatocyte-stimulating factor':ab,ti OR 'hepatocyte stimulating factor':ab,ti OR 'hybridoma growth factor':ab,ti OR 'ifn-beta 2':ab,ti OR 'il-6':ab,ti OR 'il6':ab,ti OR 'interferon beta-2':ab,ti OR 'interferon beta 2':ab,ti OR 'mgi-2':ab,ti OR 'myeloid differentiation-inducing protein':ab,ti OR 'myeloid differentiation inducing protein':ab,ti OR 'plasmacytoma growth factor':ab,ti OR 'interleukin-1beta':ab,ti OR 'interleukin 1beta':ab,ti OR 'catabolin':ab,ti OR 'il-1 beta':ab,ti OR 'interleukin-1 beta':ab,ti OR 'interleukin 1 beta':ab,ti OR 'tumor necrosis factor-alpha':ab,ti OR 'tumor necrosis factor alpha':ab,ti OR 'tnf-alpha':ab,ti OR 'tumor necrosis factor':ab,ti OR 'tumor necrosis factor ligand superfamily member 2':ab,ti OR 'tnfalpha':ab,ti OR 'cachectin-tumor necrosis factor':ab,ti OR 'cachectin tumor necrosis factor':ab,ti OR 'cachectin':ab,ti OR 'interleukin-17':ab,ti OR 'ctla-8':ab,ti OR 'il-17':ab,ti OR 'interleukin 17':ab,ti OR 'interleukin-17b':ab,ti OR 'interleukin 17b':ab,ti OR 'il-17b':ab,ti OR 'interleukin-17a':ab,ti OR 'interleukin 17a':ab,ti OR 'ctla8':ab,ti OR 'cytotoxic t lymphocyte-associated antigen 8':ab,ti OR 'cytotoxic t lymphocyte associated antigen 8':ab,ti OR 'il-17a':ab,ti OR 'interleukin-17c':ab,ti OR 'interleukin 17c':ab,ti OR 'cytokine cx2':ab,ti OR 'il-17c':ab,ti OR 'interleukin-17e':ab,ti OR 'interleukin 17e':ab,ti OR 'il-17e':ab,ti OR 'il 17e':ab,ti OR 'interleukin-25':ab,ti OR 'interleukin 25':ab,ti OR 'interleukin-17f':ab,ti OR 'interleukin 17f':ab,ti OR 'cytokine ml-1':ab,ti OR 'cytokine ml 1':ab,ti OR 'il-17f':ab,ti OR 'interleukin-8':ab,ti OR 'interleukin 8':ab,ti OR 'il-8':ab,ti OR 'il8':ab,ti OR 'monocyte-derived neutrophil chemotactic factor':ab,ti OR 'neutrophil activation factor':ab,ti OR 'lymphocyte-derived neutrophil-activating peptide':ab,ti OR 'monocyte-derived neutrophil-activating peptide':ab,ti OR 'alveolar macrophage chemotactic factor-i':ab,ti OR 'alveolar macrophage chemotactic factor i':ab,ti OR 'anionic neutrophil-activating peptide':ab,ti OR 'anionic neutrophil activating peptide':ab,ti OR 'chemokine cxcl8':ab,ti OR 'cxcl8 chemokines':ab,ti OR 'macrophage-derived chemotactic factor':ab,ti OR 'neutrophil chemotactic factor':ab,ti OR 'cxcl8 chemokine':ab,ti OR 'granulocyte chemotactic peptide-interleukin-8':ab,ti OR 'granulocyte chemotactic peptide interleukin 8':ab,ti OR 'amcf-i':ab,ti OR 'interleukin-10':ab,ti OR 'interleukin 10':ab,ti OR 'csif-10':ab,ti OR 'cytokine synthesis inhibitory factor':ab,ti OR 'il-10':ab,ti OR 'il10':ab,ti OR 'interferon-gamma':ab,ti OR 'interferon type ii':ab,ti OR 'immune interferon':ab,ti OR 'type ii interferon':ab,ti OR 'gamma-interferon':ab,ti OR 'transforming growth factor beta':ab,ti OR 'platelet transforming growth factor':ab,ti OR 'bone-derived transforming growth factor':ab,ti OR 'bone derived transforming growth factor':ab,ti OR 'tgf-beta':ab,ti OR 'tgfbeta':ab,ti OR 'milk growth factor':ab,ti OR 'c-reactive protein':ab,ti OR 'c reactive protein':ab,ti OR 'high sensitivity c-reactive protein':ab,ti OR 'high sensitivity c reactive protein':ab,ti OR 'hs-crp':ab,ti OR 'hscrp':ab,ti OR 'serum amyloid a protein':ab,ti OR 'amyloid a precursor':ab,ti OR 'amyloid a protein-related serum component':ab,ti OR 'amyloid a protein related serum component':ab,ti OR 'amyloid protein aa precursor':ab,ti OR 'amyloid protein saa':ab,ti OR 'amyloid serum protein saa':ab,ti OR 'amyloid-related serum protein':ab,ti OR 'saa':ab,ti OR 'serum a related protein':ab,ti OR 'serum amyloid a':ab,ti OR 'serum amyloid protein a':ab,ti OR 'amyloid a protein':ab,ti OR 'amyloid fibril protein aa':ab,ti OR 'amyloid protein aa':ab,ti OR 'neurotrophic*':ab,ti OR 'brain-derived neurotrophic factor':ab,ti OR 'bdnf':ab,ti OR 'brain derived neurotrophic factor':ab,ti OR 'nerve growth factor':ab,ti OR 'ngf':ab,ti OR 'nerve growth factor 1':ab,ti OR 'nerve growth factor beta subunit':ab,ti OR 'beta-nerve growth factor':ab,ti OR 'beta nerve growth factor':ab,ti OR 'ngf-1beta':ab,ti OR 'ngf 1beta':ab,ti OR 'nerve growth factor alpha subunit':ab,ti OR 'nerve growth factor gamma subunit':ab,ti OR 'glial cell line-derived neurotrophic factor':ab,ti OR 'gdnf':ab,ti OR 'glial cell line derived neurotrophic factor':ab,ti OR 'glial cell-line derived neurotrophic factor':ab,ti OR 'glial cell-derived neurotrophic factor':ab,ti OR 'glial cell derived neurotrophic factor':ab,ti OR 'glial-derived neurotrophic factor':ab,ti OR 'glial derived neurotrophic factor':ab,ti OR 'gdnf growth factor':ab,ti OR 'oxidative stress*':ab,ti OR 'malondialdehyde':ab,ti OR 'mda':ab,ti OR 'malonaldehyde':ab,ti OR 'malonylaldehyde':ab,ti OR 'malonyldialdehyde':ab,ti OR 'propanedial':ab,ti OR 'sodium malondialdehyde':ab,ti OR 'superoxide dismutase':ab,ti OR 'sod':ab,ti OR 'erythrocuprein':ab,ti OR 'hemocuprein':ab,ti OR 'cu-superoxide dismutase':ab,ti OR 'cu superoxide dismutase':ab,ti OR 'fe-zn superoxide dismutase':ab,ti OR 'fe zn superoxide dismutase':ab,ti OR 'cobalt superoxide dismutase':ab,ti OR 'mn-superoxide dismutase':ab,ti OR 'mn superoxide dismutase':ab,ti OR 'mn-sod':ab,ti OR 'mn sod':ab,ti OR 'manganese superoxide dismutase':ab,ti OR 'ag-zn superoxide dismutase':ab,ti OR 'ag zn superoxide dismutase':ab,ti OR 'iron superoxide dismutase':ab,ti OR 'fe-superoxide dismutase':ab,ti OR 'fe superoxide dismutase':ab,ti OR 'glutathione':ab,ti OR 'gsh':ab,ti OR 'nitric oxide':ab,ti OR 'no':ab,ti OR 'nitrogen monoxide':ab,ti OR 'mononitrogen monoxide':ab,ti OR 'endogenous nitrate vasodilator':ab,ti OR 'endothelium-derived nitric oxide':ab,ti OR 'metabolic*':ab,ti OR 'insulin':ab,ti OR 'regular insulin':ab,ti OR 'soluble insulin':ab,ti OR 'iletin':ab,ti OR 'insulin a chain':ab,ti OR 'novolin':ab,ti OR 'sodium insulin':ab,ti OR 'insulin b chain':ab,ti OR 'glucose':ab,ti OR 'd-glucose':ab,ti OR 'd glucose':ab,ti OR 'dextrose':ab,ti OR 'glucose monohydrate':ab,ti OR 'anhydrous dextrose':ab,ti OR 'triglycerides':ab,ti OR 'triglyceride':ab,ti OR 'triacylglycerols':ab,ti OR 'triacylglycerol':ab,ti OR 'lipoproteins':ab,ti OR 'lipoproteins, ldl':ab,ti OR 'ldl':ab,ti OR 'ldl lipoproteins':ab,ti OR 'beta-lipoprotein':ab,ti OR 'beta lipoprotein':ab,ti OR 'low-density lipoprotein':ab,ti OR 'low density lipoprotein':ab,ti OR 'beta-lipoproteins':ab,ti OR 'beta lipoproteins':ab,ti OR 'low-density lipoproteins':ab,ti OR 'low density lipoproteins':ab,ti OR 'ldl-1':ab,ti OR 'ldl(1)':ab,ti OR 'ldl1':ab,ti OR 'low-density lipoprotein 1':ab,ti OR 'low density lipoprotein 1':ab,ti OR 'ldl-2':ab,ti OR 'ldl(2)':ab,ti OR 'ldl2':ab,ti OR 'low-density lipoprotein 2':ab,ti OR 'low density lipoprotein 2':ab,ti OR 'amyloid beta-peptides':ab,ti OR 'amyloid beta peptides':ab,ti OR 'alzheimer beta-protein':ab,ti OR 'alzheimer beta protein':ab,ti OR 'alzheimer abp':ab,ti OR 'alzheimers abp':ab,ti OR 'amyloid ad-ap':ab,ti OR 'amyloid ad ap':ab,ti OR 'amyloid beta-peptide':ab,ti OR 'amyloid beta peptide':ab,ti OR 'amyloid beta-protein':ab,ti OR 'amyloid beta protein':ab,ti OR 'amyloid beta-proteins':ab,ti OR 'amyloid beta proteins':ab,ti OR 'amyloid protein a4':ab,ti OR 'beta amyloid':ab,ti OR 'beta-amyloid protein':ab,ti OR 'beta amyloid protein':ab,ti OR 'tau proteins':ab,ti OR 'tau protein':ab,ti OR 'fatty acids, volatile':ab,ti OR 'volatile fatty acids':ab,ti OR 'short-chain fatty acids':ab,ti OR 'short-chain fatty acid':ab,ti OR 'short chain fatty acid':ab,ti OR 'volatile fatty acid':ab,ti OR 'tryptophan':ab,ti OR 'levotryptophan':ab,ti OR 'l-tryptophan':ab,ti OR 'l tryptophan':ab,ti OR 'tryptophan metabolism alterations':ab,ti OR 'tryptan':ab,ti OR 'tryptacin':ab,ti OR 'trofan':ab,ti OR 'ardehydorm':ab,ti OR 'ardehydropin':ab,ti OR 'l-tryptophan-ratiopharm':ab,ti OR 'l tryptophan ratiopharm':ab,ti OR 'ratio-tryptophan':ab,ti OR 'ratio tryptophan':ab,ti OR 'lyphan':ab,ti OR 'naturruhe':ab,ti OR 'pms-tryptophan':ab,ti OR 'pms tryptophan':ab,ti OR 'optimax':ab,ti OR 'kynurenine':ab,ti | 12457262 |
| #1 | 'sleep initiation and maintenance disorders':ab,ti OR 'disorders of initiating and maintaining sleep':ab,ti OR 'dims':ab,ti OR 'sleeplessness':ab,ti OR 'insomnia disorder':ab,ti OR 'insomnia disorders':ab,ti OR 'insomnia':ab,ti OR 'insomnias':ab,ti OR 'chronic insomnia':ab,ti OR 'insomnia, chronic':ab,ti OR 'early awakening':ab,ti OR 'awakening, early':ab,ti OR 'nonorganic insomnia':ab,ti OR 'insomnia, nonorganic':ab,ti OR 'primary insomnia':ab,ti OR 'insomnia, primary':ab,ti OR 'psychophysiological insomnia':ab,ti OR 'insomnia, psychophysiological':ab,ti OR 'sleep initiation dysfunction':ab,ti OR 'dysfunction, sleep initiation':ab,ti OR 'dysfunctions, sleep initiation':ab,ti OR 'dsleep initiation dysfunctions':ab,ti OR 'insomnia'/exp | 120678 |

**Table S5.** Search strategy used for CNKI, Wanfang Data, VIP and CBM database

| Query | Searching Strategies | Results |
| --- | --- | --- |
| China National Knowledge Infrastructure (CNKI) | (SU = 神经递质 OR 5-羟色胺 OR 血清素 OR 多巴胺 OR 去甲肾上腺素 OR γ-氨基丁酸 OR 谷氨酸 OR 乙酰胆碱 OR 组胺 OR 食欲素 OR 神经肽Y OR 激素 OR 促肾上腺皮质激素释放激素 OR 促肾上腺皮质激素 OR 皮质醇 OR 氢化可的松 OR 褪黑素 OR 生长激素 OR 催乳素 OR 瘦素 OR 胃饥饿素 OR 细胞因子 OR 白细胞介素-6 OR 白细胞介素-1β OR 肿瘤坏死因子α OR 白细胞介素-17 OR 白细胞介素-8 OR 白细胞介素-10 OR γ干扰素 OR 转化生长因子β OR C反应蛋白 OR 血清淀粉样蛋白A OR 神经营养因子 OR 脑源性神经营养因子 OR 神经生长因子 OR 胶质细胞源性神经营养因子 OR 氧化应激 OR 丙二醛 OR 超氧化物歧化酶 OR 谷胱甘肽 OR 一氧化氮 OR 代谢指标 OR 胰岛素 OR 葡萄糖 OR 甘油三酯 OR 低密度脂蛋白 OR β-淀粉样蛋白 OR tau蛋白 OR 短链脂肪酸 OR 色氨酸 OR 犬尿氨酸) AND (SU = 失眠 OR 失眠症 OR 不寐 OR 早醒 OR 原发性失眠 OR 慢性失眠 OR 睡眠障碍 OR 睡眠异常 OR 睡眠不足 OR 睡眠困难 OR 入睡困难 OR 入睡和睡眠失调 OR 入睡和睡眠障碍 OR 睡眠起始功能障碍) | 1344 |
| Wanfang Data | 主题:(神经递质 OR 5-羟色胺 OR 血清素 OR 多巴胺 OR 去甲肾上腺素 OR γ-氨基丁酸 OR 谷氨酸 OR 乙酰胆碱 OR 组胺 OR 食欲素 OR 神经肽Y OR 激素 OR 促肾上腺皮质激素释放激素 OR 促肾上腺皮质激素 OR 皮质醇 OR 氢化可的松 OR 褪黑素 OR 生长激素 OR 催乳素 OR 瘦素 OR 胃饥饿素 OR 细胞因子 OR 白细胞介素-6 OR 白细胞介素-1β OR 肿瘤坏死因子α OR 白细胞介素-17 OR 白细胞介素-8 OR 白细胞介素-10 OR γ干扰素 OR 转化生长因子β OR C反应蛋白 OR 血清淀粉样蛋白A OR 神经营养因子 OR 脑源性神经营养因子 OR 神经生长因子 OR 胶质细胞源性神经营养因子 OR 氧化应激 OR 丙二醛 OR 超氧化物歧化酶 OR 谷胱甘肽 OR 一氧化氮 OR 代谢指标 OR 胰岛素 OR 葡萄糖 OR 甘油三酯 OR 低密度脂蛋白 OR β-淀粉样蛋白 OR tau蛋白 OR 短链脂肪酸 OR 色氨酸 OR 犬尿氨酸) and 主题:(失眠 OR 失眠症 OR 不寐 OR 早醒 OR 原发性失眠 OR 慢性失眠 OR 睡眠障碍 OR 睡眠异常 OR 睡眠不足 OR 睡眠困难 OR 入睡困难 OR 入睡和睡眠失调 OR 入睡和睡眠障碍 OR 睡眠起始功能障碍) | 12299 |
| VIP Database for Chinese Technical Periodicals (VIP) | M=(神经递质 OR 5-羟色胺 OR 血清素 OR 多巴胺 OR 去甲肾上腺素 OR γ-氨基丁酸 OR 谷氨酸 OR 乙酰胆碱 OR 组胺 OR 食欲素 OR 神经肽Y OR 激素 OR 促肾上腺皮质激素释放激素 OR 促肾上腺皮质激素 OR 皮质醇 OR 氢化可的松 OR 褪黑素 OR 生长激素 OR 催乳素 OR 瘦素 OR 胃饥饿素 OR 细胞因子 OR 白细胞介素-6 OR 白细胞介素-1β OR 肿瘤坏死因子α OR 白细胞介素-17 OR 白细胞介素-8 OR 白细胞介素-10 OR γ干扰素 OR 转化生长因子β OR C反应蛋白 OR 血清淀粉样蛋白A OR 神经营养因子 OR 脑源性神经营养因子 OR 神经生长因子 OR 胶质细胞源性神经营养因子 OR 氧化应激 OR 丙二醛 OR 超氧化物歧化酶 OR 谷胱甘肽 OR 一氧化氮 OR 代谢指标 OR 胰岛素 OR 葡萄糖 OR 甘油三酯 OR 低密度脂蛋白 OR β-淀粉样蛋白 OR tau蛋白 OR 短链脂肪酸 OR 色氨酸 OR 犬尿氨酸) AND M=(失眠 OR 失眠症 OR 不寐 OR 早醒 OR 原发性失眠 OR 慢性失眠 OR 睡眠障碍 OR 睡眠异常 OR 睡眠不足 OR 睡眠困难 OR 入睡困难 OR 入睡和睡眠失调 OR 入睡和睡眠障碍 OR 睡眠起始功能障碍) | 2266 |
| Chinese Biomedical Literature Database (CBM) | (神经递质 OR 5-羟色胺 OR 血清素 OR 多巴胺 OR 去甲肾上腺素 OR γ-氨基丁酸 OR 谷氨酸 OR 乙酰胆碱 OR 组胺 OR 食欲素 OR 神经肽Y OR 激素 OR 促肾上腺皮质激素释放激素 OR 促肾上腺皮质激素 OR 皮质醇 OR 氢化可的松 OR 褪黑素 OR 生长激素 OR 催乳素 OR 瘦素 OR 胃饥饿素 OR 细胞因子 OR 白细胞介素-6 OR 白细胞介素-1β OR 肿瘤坏死因子α OR 白细胞介素-17 OR 白细胞介素-8 OR 白细胞介素-10 OR γ干扰素 OR 转化生长因子β OR C反应蛋白 OR 血清淀粉样蛋白A OR 神经营养因子 OR 脑源性神经营养因子 OR 神经生长因子 OR 胶质细胞源性神经营养因子 OR 氧化应激 OR 丙二醛 OR 超氧化物歧化酶 OR 谷胱甘肽 OR 一氧化氮 OR 代谢指标 OR 胰岛素 OR 葡萄糖 OR 甘油三酯 OR 低密度脂蛋白 OR β-淀粉样蛋白 OR tau蛋白 OR 短链脂肪酸 OR 色氨酸 OR 犬尿氨酸) AND (失眠 OR 失眠症 OR 不寐 OR 早醒 OR 原发性失眠 OR 慢性失眠 OR 睡眠障碍 OR 睡眠异常 OR 睡眠不足 OR 睡眠困难 OR 入睡困难 OR 入睡和睡眠失调 OR 入睡和睡眠障碍 OR 睡眠起始功能障碍) | 9684 |

**Table S6**. Risk of bias assessment for studies included in the meta-analysis of IL-6

| Std_Eff | Coefficient | Std. err. | t | P>t | [95% conf. | interval] |
| --- | --- | --- | --- | --- | --- | --- |
| slope | -.4152694 | 2.079585 | -0.2 | 0.847 | -5.210801 | 4.380262 |
| bias | 7.689811 | 8.881022 | 0.87 | 0.412 | -12.78986 | 28.16948 |

*Note.* Number of studies = 10; Root MSE = 4.907; Test of H0: no small-study effects.

**Table S7**. Assessment of GRADE criteria (risk of bias, inconsistency, imprecision, and publication bias)

| Outcome | No. of participants (studies) | Risk of bias^1^ | Inconsistency^2^ | Imprecision^3^ | Publication bias^4^ | Other condersations^5^ | SMD  (95% CI) | Certainty of evidence |
| --- | --- | --- | --- | --- | --- | --- | --- | --- |
| Cortisol | 740 (8) | Not serious | Serious | Not serious | Not detected | None | 3.65  (2.35–4.95) | Very low  (⊕◯◯◯) |
| CRH | 610 (7) | Not serious | Serious | Serious | Not detected | None | 0.68  (−1.02–2.37) | Very low  (⊕◯◯◯) |
| TNF-α | 492 (6) | Not serious | Serious | Not serious | Not detected | None | 0.82  (0.27–1.37) | Very low  (⊕◯◯◯) |
| IL-1β | 472 (7) | Not serious | Serious | Serious | Not detected | None | −0.42  (−1.43–0.59) | Very low  (⊕◯◯◯) |
| IL-2 | 255 (3) | Not serious | Not serious | Not serious | Not detected | None | 0.50  (0.19–0.81) | Low  (⊕⊕◯◯) |
| IL-4 | 356 (4) | Not serious | Serious | Serious | Not detected | None | 0.22  (−0.88–1.32) | Very low  (⊕◯◯◯) |
| IL-6 | 1013 (10) | Not serious | Serious | Not serious | Not serious | None | 1.47  (0.76–2.18) | Very low  (⊕◯◯◯) |
| IL-10 | 419 (5) | Not serious | Serious | Serious | Not detected | None | – 0.19  (– 0.66–0.28) | Very low  (⊕◯◯◯) |
| IFN-γ | 422 (5) | Not serious | Not serious | Not serious | Not detected | None | 0.22  (0.02–0.42) | Low  (⊕⊕◯◯) |
| CRP | 30468 (5) | Not serious | Serious | Not serious | Not detected | None | 0.40  (0.08–0.72) | Very low  (⊕◯◯◯) |
| BDNF | 811 (7) | Not serious | Serious | Serious | Not detected | None | −0.89  (−2.20–0.41) | Very low  (⊕◯◯◯) |
| NPY | 220 (3) | Not serious | Serious | Serious | Not detected | None | −0.47  (−1.59– 0.64) | Very low  (⊕◯◯◯) |

*Note.* CI = confidence interval; CRH = corticotropin-releasing hormone; TNF-α = tumor necrosis factor-α; IL-1β = interleukin-1β; IL-2 = interleukin-2; IL-4 = interleukin-4; IL-6 = interleukin-6; IL-10 = interleukin-10; IFN-γ = interferon-γ; CRP = C-reactive protein; BDNF = brain-derived neurotrophic factor; and NPY = neuropeptide Y.

^1^The evidence was not downgraded for risk of bias when the majority of included studies were rated as high quality according to the AHRQ or NOS tool. ^2^The evidence was downgraded by (−1) for inconsistency or heterogeneity, assessed based on differing effect estimates or I² statistics, if I² >50% and no plausible explanation was identified. ^3^The evidence was downgraded by (−1) for imprecision when confidence intervals were wide or crossed the line of no effect. ^4^The evidence was downgraded by (−1) for publication bias, assessed by asymmetry of funnel plots or significant Egger’s test results. ^5^The certainty of evidence was upgraded by (+1) if a dose-response relationship was reported, and downgraded (−1) if the evidence was based on narrative synthesis or meta-analysis including only two studies.
